# Supplementary material for: Valve turning towards on-cycle in cobalt-catalyzed Negishi-type cross-coupling
Source: Nat Commun. 2023 Aug 2;14:4638. doi: 10.1038/s41467-023-40269-y (PMC10397345; doi:10.1038/s41467-023-40269-y)
Supplement: Supplementary file 1 — Supplementary Information [file 41467_2023_40269_MOESM1_ESM.pdf]

Supplementary Information

Valve Turning towards On-Cycle in Cobalt-Catalyzed Negishi-type Cross-coupling

Contents

1 Supplementary Methods 2

1.1 General Information 2

1.2 Titration of Organozinc Reagents Using Iodine[1] 2

1.3 Preparation of organozinc reagents 2

2 Supplementary Discussion 3

2.1 Experimental Procedures for the cobalt catalyzed Negishi-coupling 3

2.2 Procedures for getting monocrystal 4

2.3 General procedures for EPR studies 5

2.4 General procedures for XAFS studies 6

2.5 General procedures for CV 9

2.6 General procedures for *in-situ* IR 10

3 Supplementary Notes 12

3.1 Computational details 12

3.2 Detail descriptions for products 14

3.3 Copies of product NMR Spectra 29

4 Supplementary References 83

1 Supplementary Methods

1.1 General Information

All glassware was oven dried at 110 °C for hours and then flame-dried under vacuum before used. During the raining season, cobalt catalyzed reactions were carried out under an argon atmosphere in glovebox (Vigor). Schlenk tube (10 mL, 22*55mm) was taken from SYNTHWARE GLASS. GC yields were detected by SHIMADZU™ GC-2014 gas chromatography using naphthalene as internal standard. Molecular weights of products were detected by SHIMADZU™ GCMS-QP2010 SE gas chromatography mass spectrometry. All new compounds were characterized by high resolution mass spectra (HRMS) (Bruker UltiMate3000 & Compact). IR spectra was recorded on a Mettler Toledo React IR 15 spectrometer using a diamond comb. The *in-situ* IR studies were performed in a three-necked micro reactor. Unless otherwise noted, materials and solvents were obtained from commercial suppliers (Alfa, Sigma, Adamas, Aladdin, Energy, HEOWNS, Innochem, Bidepharm etc.) and were used without further purification. Thin layer chromatography (TLC) employed glass 0.25 mm silica gel plates. Visualization of spots on TLC plate was accomplished with UV light (254 nm) or staining over phosphomolybdic acid hydrate heating by heat gun. Flash chromatography column was packed with 200-300 mesh silica gel in petroleum (bp. 60-90 °C) and ethyl acetate. X-ray absorption measurements were acquired in transmission mode at beamline TPS (Taiwan Photon Source) 44A at National Synchrotron Radiation Research Center (NSSRC) in Taiwan. A pure Co foil spectrum (edge energy 7709 eV) was acquired simultaneously with each measurement for energy calibration. Multiple scans were taken to reduce the noise. Electron Paramagnetic Resonance (EPR) was carried on Bruker A300. ^{1}H and ^{13}C NMR data were recorded with Bruker Advance III (400 MHz) spectrometer with tetramethylsilane as an internal standard. All chemical shifts (δ) were reported in ppm and coupling constants (*J*) in Hz.

1.2 Titration of Organozinc Reagents Using Iodine[1]

To a solution of crude solid zinc reagent (50 mg) in dry THF (1 mL) was titrated with I_2 solution in THF (1.0 M). The concentration of the active species (in mmol/mg) was calculated accordingly.

1.3 Preparation of organozinc reagents

Method 1 (for compound **3ga-3gi**): Organozinc reagents were prepared via transmetallation.

Anhydrous $ZnCl_2$ solution in THF (1.0 M) was treated with commercially available Grignard reagents (1 equiv.) at 0 °C dropwise. The resulting solution was stirred at 0 °C for 30 min followed by removing THF under high vacuum. The crude organozinc reagents were titrated afterwards.

Method 2 (for **3gj-3gn**): Grignard reagents were prepared via direct magnesium insertion.[2] The reactions were monitored by GC until full conversion. The magnesium reagents in THF were titration by I_2 solution at 0 °C followed by transmetallation ($ZnCl_2$). The resulting zinc reagent was titrated again with I_2 solution.

Method 3 (for **3vo**): To a solution of aryl bromide in THF was cooled down to -78 °C. ^{n}BuLi (1.6 M in hexane) was added dropwise and stirred at the same temperature until full conversion (monitored by GC). Then the lithium reagent was adding dropwise to $ZnCl_2$ (1 equiv.) in THF at -40 °C. After solvent removed, the crude organozinc reagents were titrated by I_2 solution.

2 Supplementary Discussion

2.1 Experimental Procedures for the cobalt catalyzed Negishi-coupling

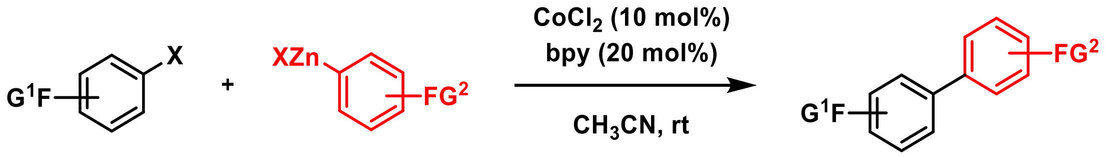

Reaction conditions for Co-catalyzed Negishi cross-coupling (method A): A 10 mL schlenk tube was equipped with a magnetic stir bar. Electrophiles (0.2 mmol, bromides, chlorides or NHPI ester), $CoCl_2$ (0.02 mmol, 2.6 mg), 2,2'-bipyridine (0.04 mmol, 6.3 mg) were added to the schlenk tube. Then MeCN (0.5 mL) was added to schlenk tube, stirred for seconds. Corresponding solid organozinc reagents were added and the remaining MeCN (0.5 mL) was added along the wall of tube. The reaction was monitored by TLC and GC-MS to identify the reaction time. And the reaction was stopped when electrophiles were fully converted. The reaction was quenched by saturated NH_4Cl aqueous solution and extracted with EtOAc three times. The combined organic layer was dried over anhydrous Na_2SO_4 and was evaporated in vacuum. The desired products were obtained in the corresponding yields after purification by flash chromatography on 200-300 mesh silica gel.

Reaction conditions for Co-catalyzed Negishi cross-coupling (method B): 3ib, 3mb, 3a4b, 3a5a: The experimental procedure was like method A in the absence of 2,2'-bipyridine.

Supplementary Table 1 Optimization of reaction conditions

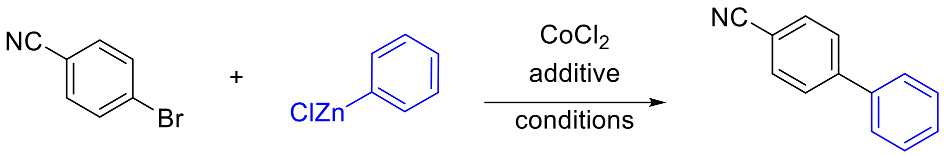

1a, 1 equiv **2a**, 1.5 equiv **3aa**

Entry	Solvent	Catalyst (x mol%)	Ligand (x mol%)	**3aa,** Yield (%)[b]
1	THF	$CoCl_2$ (10)	-	trace
2	CH_3CN	$CoCl_2$ (10)	-	62
3	DMF	$CoCl_2$ (10)	-	12
4	toluene	$CoCl_2$ (10)	-	trace
5	CH_2Cl_2	$CoCl_2$ (10)	-	13
6	CH_3CN	$CoCl_2$ (10)	TMEDA (20)	61
7	CH_3CN	$CoCl_2$ (10)	TMCD (20)	55
8[a]	CH_3CN	$CoCl_2$ (10)	bpy (20)	72 (71)[c]
9	CH_3CN	$CoCl_2$ (5)	bpy (20)	52
10	CH_3CN	$CoCl_2$ (15)	bpy (20)	62
11	CH_3CN	$CoCl_2$ (20)	bpy (20)	60
12	CH_3CN	$CoBr_2$ (10)	-	61
13	CH_3CN	-	-	n.d.

[a] Reaction conditions: PhZnCl (0.30 mmol), **1a** (0.20 mmol), $CoCl_2$ (10 mol%), bpy (20 mol%), and CH_3CN (1.0 mL); react in a Schlenk tube at 20 °C. [b] Yields were determined by GC analysis with naphthalene as the internal standard at r.t. [c] Isolated yield.

When 2,5-dibromothiophene **1e** reacted with *p*-fluorophenylzinc reagent **2d** under the optimized conditions, followed by iodolysis, iodo-thiophene 3ed was formed in 12% GC yield

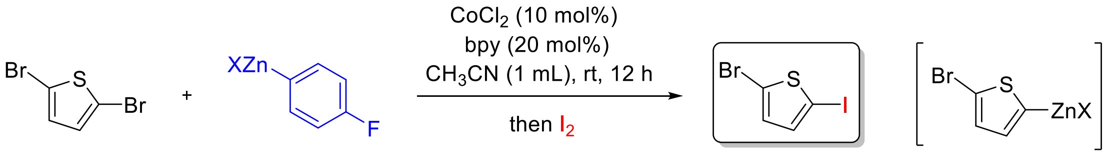

1e **2d** **3ed** (12%[c])

Scheme S1 The experiment of zincation of electrophile; 1e (0.2 mmol), 2d (0.3 mmol).

2.2 Procedures for getting monocrystal

$CoCl_2$ and bpy (2 equiv.) were added to MeCN to afford red solid (represented as $CoCl_2bpy_2$). After filtration, the solid was dissolved in methanol. The crystal was generated by evaporation of MeOH solvent. Its CCDC number is 2040561.

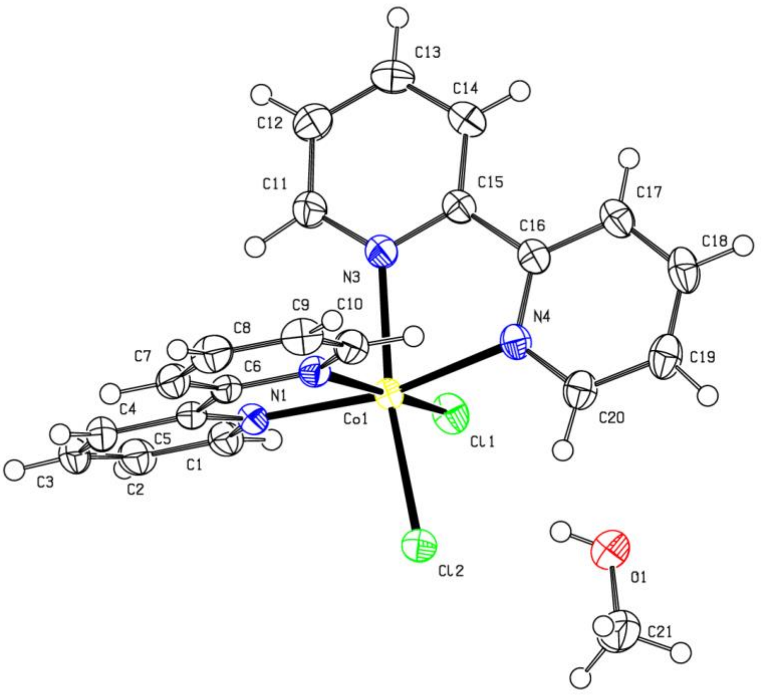

Supplementary Figure 1 Crystallographic referee. Yellow represent Cobalt, blue represent Nitrogen, green represent Chloride, red represent Oxygen, white represent Carbon, smaller white represent Hydrogen.

2.3 General procedures for EPR studies

Electron Paramagnetic Resonance (EPR) was carried out on Bruker A300. $CoCl_2$ (0.05 mmol), bpy (0.1 mmol) and MeCN (0.5 mL) were added to NMR tube in glovebox. **PhZnCl** (0.5 mmol) was dissolved in MeCN (0.5 mL) and added to the catalyst under -50 °C. The temperature was warmed up to -30 °C for 10 min. And then put NMR tube to liquid nitrogen before test. The same sample was warmed up to -20 °C, -10 °C, 0 °C, and room temperature before recording with the machine. Co(I) and Co(III) are EPR silent.We fitted the Co(II) signals. And one of the result was showed. (g_x=2.266, g_y=2.209, g_z=2.014; A_x=3.34059, A_y=13.045, A_z=104.195)

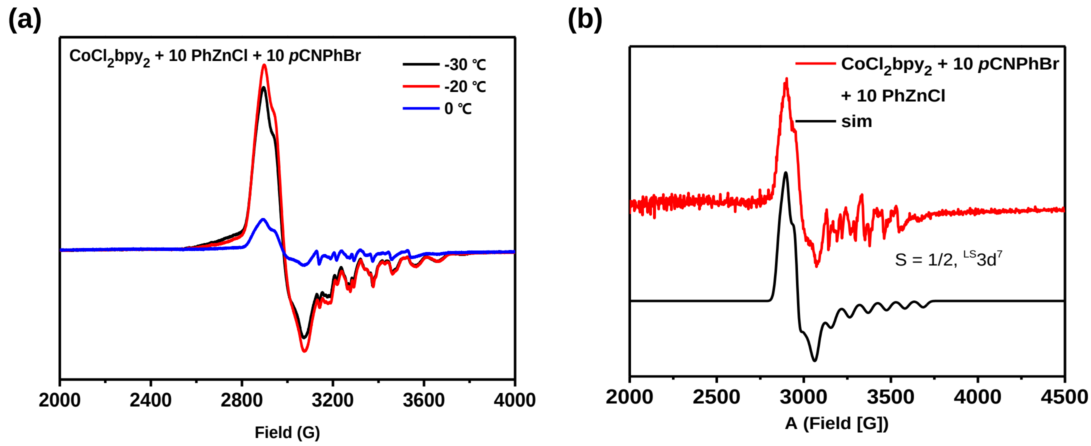

Supplementary Figure 2 CW X-band EPR experiments

2.4 General procedures for XAFS studies

The edge energy of the X-Ray absorption near edge structure (XANES) spectrum was determined from the inflection point (the first zero point of second derivative) of the edge. The data procedures were carried out using the Athena software package using standard methods. Standard procedures based on Artemis software (Demeter 0.9.26) were used to extract the extended X-ray absorption fine structure (EXAFS) data. The coordination parameters were obtained by the least square fit in R-space of the nearest neighbor, k^2-weighted Fourier transform data.

The BP86 function and unclosed shell were used for initial DFT geometry optimization in ORCA 4.0, with Co using CP(PPP) basis set and other atoms using def2-TZVP. TightSCF convergence criteria and the SlowConv criterion were employed. The optimized structures were then used for TDDFT calculations with 100 roots, MaxDim of 900, and DoQuad True. TDDFT calculations were performed with BP86 over the entire valence manifold for both spin-up (OrbWin = 0) and spin-down (OrbWin = 1) transitions. ZORA calculation was applied. The calculated energies and intensities were broadened using the line-broadening script within ORCA with half-widths of 1.5 eV to account for core-hole lifetime and instrument broadening. Finally, the calculated pre-edge energies were shifted up by 17.2 eV to compare with experimental spectra. [2-4]

Fourier Transform (FT) Magnitude of Co(0) species in THF and Co foil were displayed in article **Figure 2A-III**. We add EXAFS fitting results here to prove that formed Co(0) has lower coordination number and shorter Co-Co bond length in average, which all indicates the formation of small Co(0) nanoparticles (**Supplementary Table 2**).

Supplementary Table 2 EXAFS fitting results**.**

	Path	amp	CN	R	DW	R-factor
Co foil	Co-Co	0.79	12	2.495	0.0064	0.00124
$CoBr_2$ + 5PhZnCl	Co-Co	0.79	7 (1)	2.442	0.0150	0.01234

We first investigated the mechanistic pathway of Co-catalyzed Negishi cross-coupling under CH_3CN (**Supplementary Figure 3**). The data revealed that Co(I) species in acetonitrile could be stabilized at -30 °C. But the Co(I) species may undergo a disproportionation reaction leading to cobalt nanoparticles upon the temperature increasing. The reaction rate of oxidative addition may be faster than disproportionation using the electron-deficient electrophile. So the cross-coupling

undergoes under MeCN. However, the reaction rate of oxidative addition is too slow to compete with disproportionation via the electron rich electrophile. While the addition of bpy can improve the yield, we therefore explore the detailed mechanistic investigations of Co-catalyzed C_{sp2}-C_{sp2} cross-coupling under bpy and MeCN.

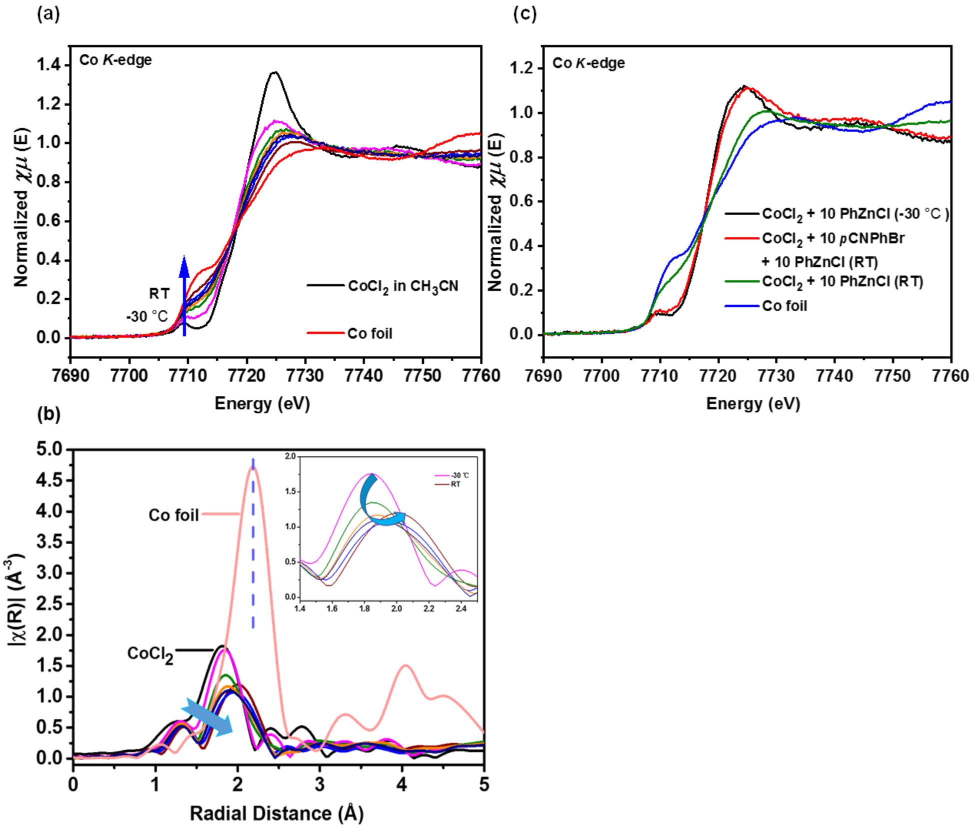

Supplementary Figure 3 (**a**) Normalized Co *K*-edge XANES spectra of $CoCl_2$ and **PhZnCl (**10 equiv.) in CH_3CN from -30 °C to rt; (**b**) FT-XAFS data of the $CoCl_2$ in MeCN and the reduction of $CoCl_2$ using PhZnCl (temperature from -30 °C to rt); (**c**) $CoCl_2$ and **PhZnCl** (10 equiv.) at -30 °C; $CoCl_2$ with compound **1a** (10 equiv.) and **PhZnCl (**10 equiv.) at rt.

Supplementary Table 3 The fitting results of $CoCl_2bpy_2$ solid

	Co-N	Co-Cl	Co-C1	Co-C2
N	4	2	4	4
amp	1	1	1	1
ΔE	-0.21(6)			
R	2.13(1)	2.38(1)	2.81(3)	3.02(2)
σ^2	0.00962	0.00596	0.00717	0.00097

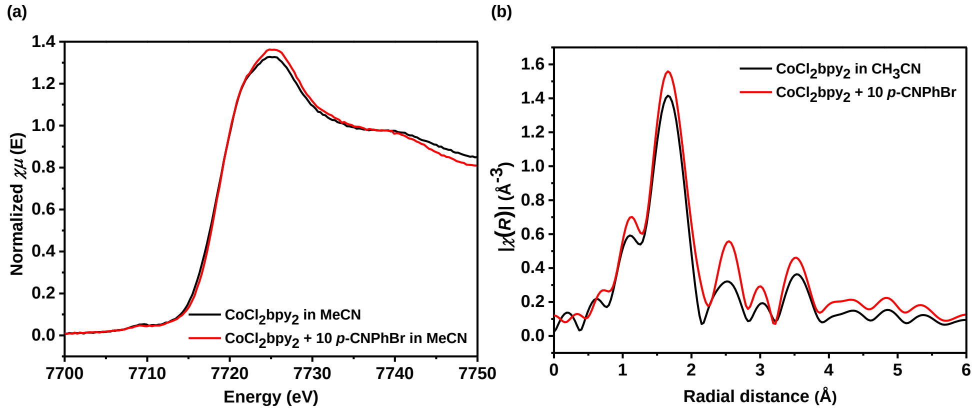

Supplementary Figure 4 The XANES and FT-XAFS of $CoCl_2bpy_2$ in CH_3CN and $CoCl_2bpy_2$ with **1** (10 equiv.) in MeCN.

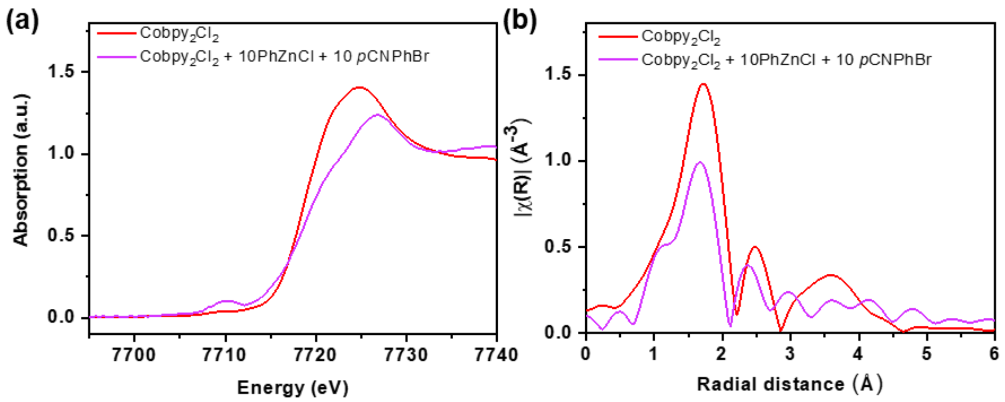

Supplementary Figure 5 (a) Normalized Co K-edge XANES spectra of $CoCl_2bpy_2$, 1a (10 equiv.) and PhZnCl (10 equiv.) in CH_3CN. (b) FT-XAFS data of $CoCl_2bpy_2$, 4-bromobenzonitrile (10 equiv.) and PhZnCl (10 equiv.).

Supplementary Table 4 The fitting results of $CoCl_2bpy_2$ and PhZnCl (10 equiv.) in MeCN

Sample	Path	CN	σ^2	ΔE (eV)	R (Å)	R-factor	FT-range (Å)	R-range (Å)
$Cobpy_2Cl_2$	Co-N	4	0.00574	2.5	2.147	0.00114	2.3-9.5	1.25-2.9
	Co-Cl	2	0.00661		2.427			
	Co-C	4	0.00345		3.036			
$Cobpy_2Cl_2$+ 10PhZnCl	Co-N	2	0.00868	2.5	2.070	0.00489	2.3-9.5	1.25-2.75
	Co-Cl	1	0.00758		2.295			
	Co-C	2	0.00266		2.931			

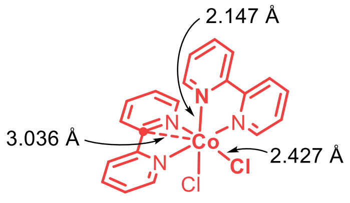

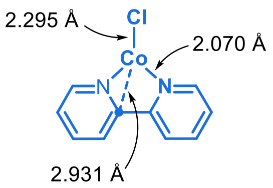

We have tried to add Co(I) fits with two bipy. The structure Cobpy$_2$Cl does not match (Supplementary Figure 5).

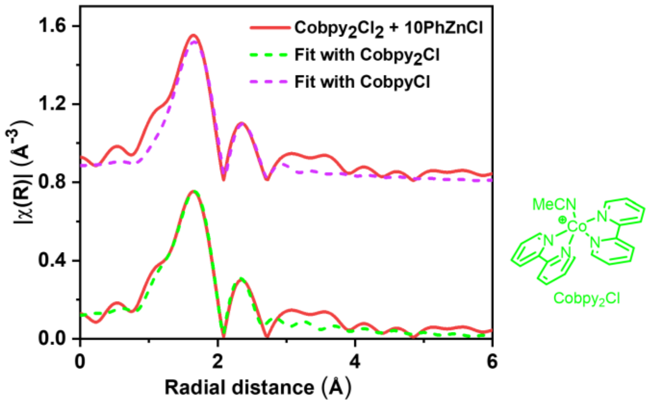

Supplementary Figure 6 EXAFS Fit of $CoCl_2bpy_2$ and PhZnCl (10 equiv.) in MeCN.

The R-factor get slightly better, and the low R parts was better fitted. However, the debye-waller factor of the first shell Co-N is incredibly large, which makes the fitting unlikely to be trusted.

Supplementary Table 5 The fitting results compared with Cobpy$_2$Cl and CobpyCl

Fitting model	Path	CN	σ^2	ΔE (eV)	R (Å)	R-factor
	Co-N	4	0.02360		2.071	
Cobpy$_2$Cl	Co-Cl	1	0.00456	1.0	2.272	0.00403
	Co-C	4	0.01417		2.917	
	Co-N	2	0.00868		2.070	
CobpyCl	Co-Cl	1	0.00758	2.5	2.295	0.00489
	Co-C	2	0.00266		2.931	

2.5 General procedures for CV

Cyclic voltammograms were recorded with a CorrTest® CS2350H bipotentiostat at room temperature. In a three-necked reactor, $^{n}Bu_4NPF_6$ (0.5 mmol in 6 mL MeCN) was used as the supporting electrolyte. 4-bromobenzonitrile (**1a**, 0.05 mmol, blue line) and 4-iodo-*N*,*N*-dimethylaniline (**1d**, 0.05 mmol, red line) was added respectively. The scan range is between -3.0 V to 0.5 V (negative). The scan rate was 50 mV/s. All potentials were referenced against the Ag/AgCl reference electrode.

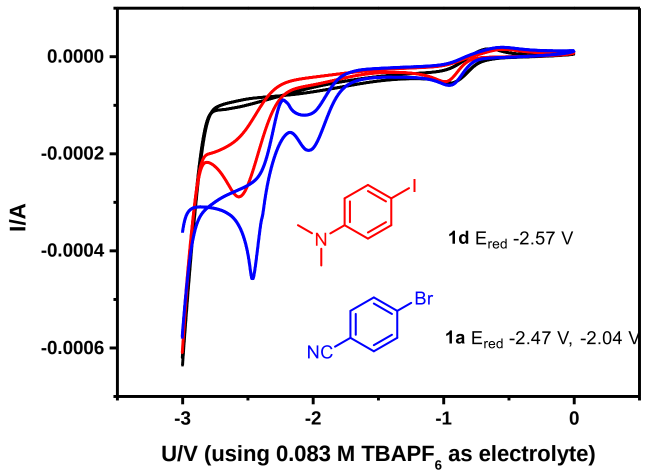

Supplementary Figure 7 Reductive potential of 1a and 1d using cyclic voltammetry (CV)

2.6 General procedures for *in-situ* IR

In a three-necked reactor, we used cooling bath to keep the reaction temperature at 0 °C. $CoCl_2$ (0.2 M in MeCN), bpy (0.4 M in MeCN), 4-bromobenzonitrile (**1a**, 1 M in MeCN), **PhZnCl** (0.4 M) in MeCN were added in sequence at 0 °C. The yield was identified by GC using naphthalene as internal standard after IR tests (every 10 seconds for one scan). The initial rates were the k value of the slope (normally within 200s). When we change the concentration of **1a** in $CoCl_2$ and bpy system at zero degree, the initial rates showed zero order. This means oxidative addition is not the determine step of the reaction.

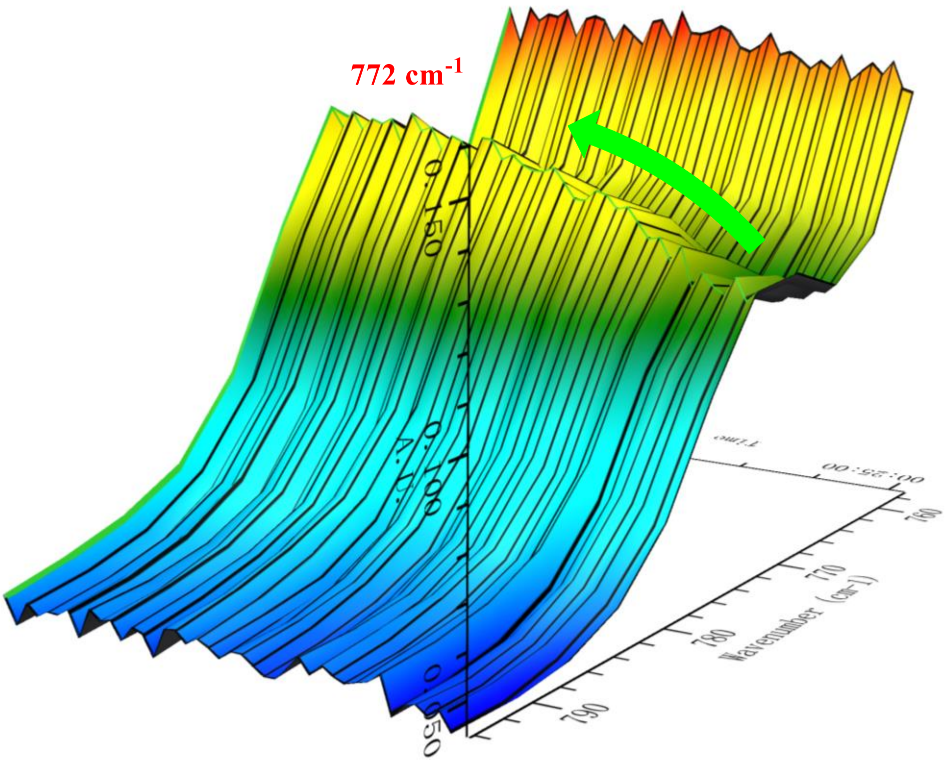

Supplementary Figure 8 Local 3D *in-situ* IR spectrum (794 cm^{-1} to 762cm^{-1}). Reaction conditions: 1 (2 mmol), $CoCl_2$ (0.2 mmol), bpy (0.4 mmol), MeCN (10 mL) at zero degree.

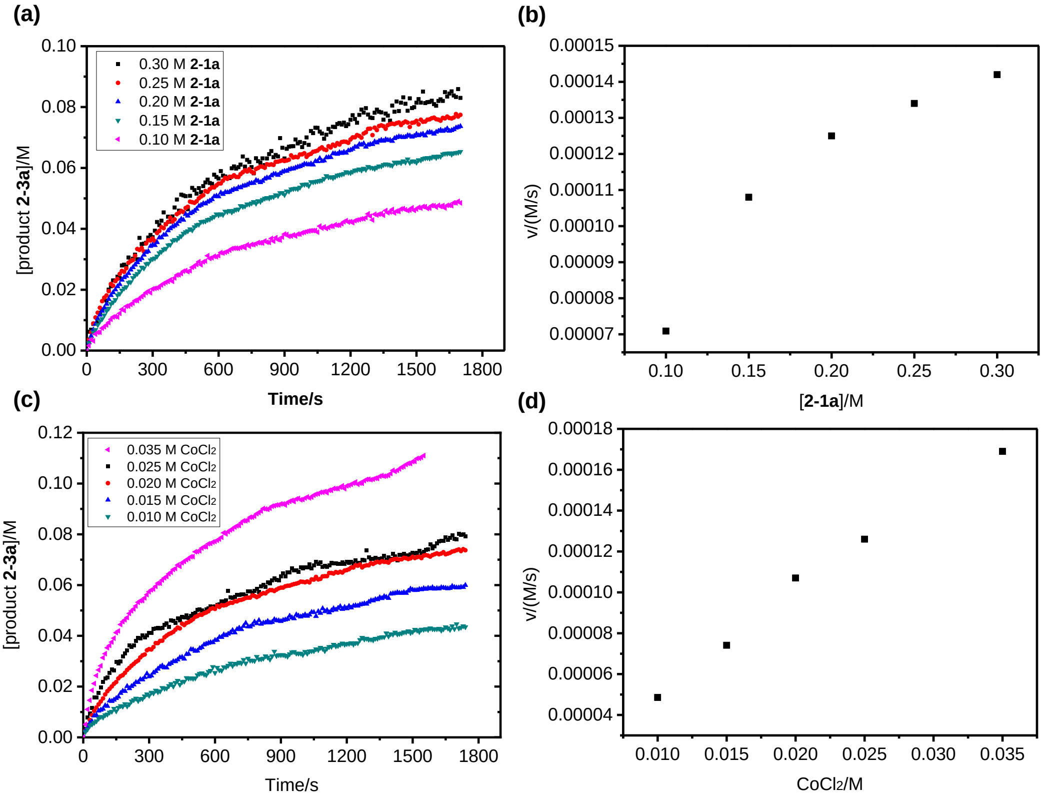

Supplementary Figure 9 Initial rates of electrophile **1a** and $CoCl_2$ (method B).

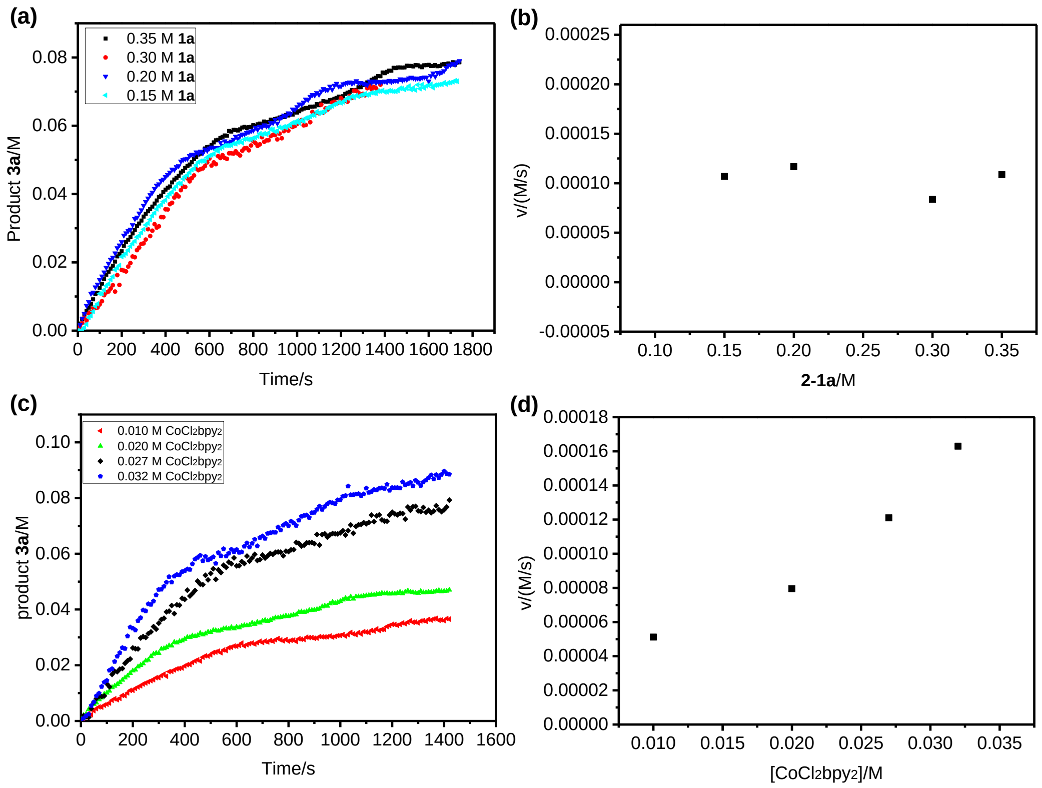

Supplementary Figure 10 Initial rates of electrophile **1a** and $CoCl_2bpy_2$ (method A).

3 Supplementary Notes

3.1 Computational details

Computational methods: All DFT calculations were carried out using the Gaussian 09 series of programs[5]. Geometries of intermediates and transition states were optimized using dispersion-corrected (U)B3LYP-D3 functional[6-7] with a standard def2-SVP[8] basis set in the gas phase. Vibrational frequency calculations were performed for all stationary points to confirm if each optimized structure is a local minimum or a transition state structure. All optimized transition state structures have only one imaginary (negative) frequency, and all minima (reactants, products, and intermediates) have no imaginary frequencies. The (U)M06 functional[9] with a standard def2-TZVP[5] basis set was used for single-point energy calculations in solution. Solvation energy corrections were calculated in acetonitrile as solvent with the SMD continuum solvation model[10] based on the gas-phase optimized geometries. The 3D images of structures were prepared using CYLView[11].

The Gibbs free energy profile of singlet Co(I)-catalyzed cross-coupling reaction

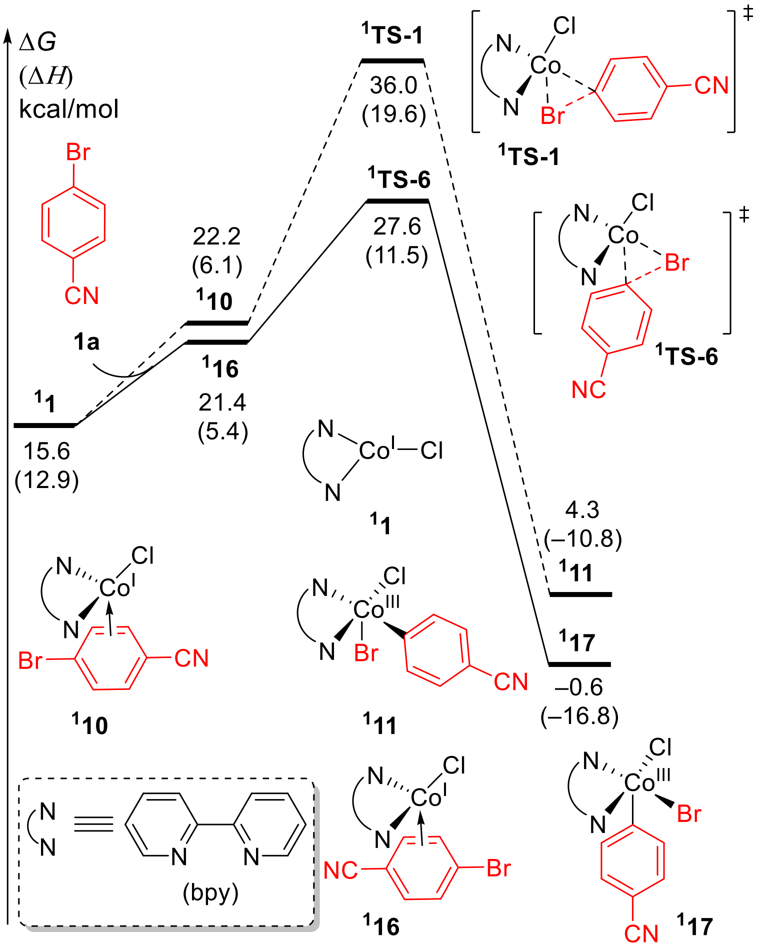

Supplementary Figure 11. The Gibbs free energy profile of singlet Co(I)-catalyzed cross-coupling reaction. Calculations were performed at the M06/def2-TZVP/SMD(acetonitrile)//B3LYP-D3/def2-SVP level of theory.

The Gibbs free energy profile of singlet Co(I)-catalyzed cross-coupling reaction is shown in Supplementary Figure 9. Our computational results show that the singlet Co(I) complex ^{1}**1** is highly unstable compared with the triplet structure ^{3}**1**. It is also noteworthy that the activation free energy of C-Br oxidative addition transition state ^{1}**TS-1** is 23.1 kcal/mol higher than that of triplet transition state ^{3}**TS-1**. Therefore, the singlet Co(I)-catalyzed cross-coupling pathway was ruled out.

Different conformations considered for C–Br oxidative addition process

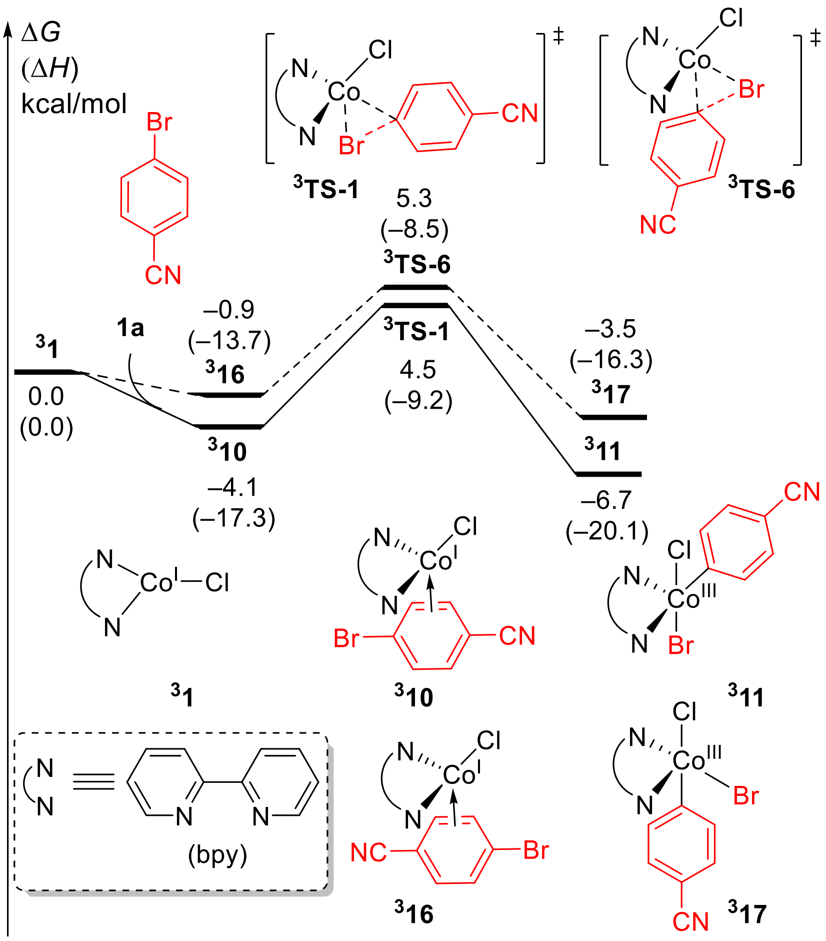

Supplementary Figure 12. Different conformations of transition states and intermediates considered for Co(I)-catalyzed C(Aryl)-Br oxidative addition. Calculations were performed at the (U)M06/def2-TZVP/SMD(acetonitrile)//B3LYP-D3/def2-SVP level of theory.

3.2 Detail descriptions for products

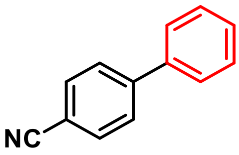

3aa

[1,1'-biphenyl]-4-carbonitrile (3aa[12]): light yellow solid was obtained with 71% isolated yield (method A, 0.2 mmol scale, 25.4 mg). ^{1}H NMR (400 MHz, Chloroform-*d*) δ 7.68 (q, *J* = 8.4 Hz, 4H), 7.61 – 7.54 (m, 2H), 7.51 – 7.37 (m, 3H). ^{13}C NMR (101 MHz, Chloroform-*d*) δ 145.51, 139.00, 132.47, 129.01, 128.56, 127.60, 127.11, 118.86, 110.74.

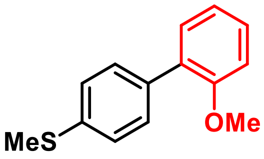

3bb

(2'-methoxy-[1,1'-biphenyl]-4-yl)(methyl)sulfane (3bb[13]): white solid was obtained with 63% isolated yield (method A, 0.2 mmol scale, 29.0 mg). ^{1}H NMR (400 MHz, Chloroform-*d*) δ 7.52 – 7.44 (m, 2H), 7.35 – 7.27 (m, 4H), 7.06 – 6.95 (m, 2H), 3.81 (s, 3H), 2.51 (s, 3H). ^{13}C NMR (101 MHz, Chloroform-*d*) δ 156.38, 136.94, 135.28, 130.62, 129.96, 129.89, 128.57, 127.11, 126.91, 126.21, 120.83, 111.14, 55.50, 15.87.

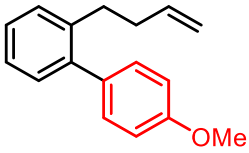

3cc

2-(but-3-en-1-yl)-4'-methoxy-1,1'-biphenyl (3cc[14]): white liquid was obtained with 36% isolated yield (method A, 0.2 mmol scale, 17.2 mg). ^{1}H NMR (400 MHz, Chloroform-*d*) δ 7.30 – 7.26 (m, 2H), 7.24 – 7.19 (m, 4H), 6.98 – 6.90 (m, 2H), 5.78 – 5.66 (m, 1H), 4.99 – 4.83 (m, 2H), 3.85 (s, 3H), 2.76 – 2.59 (m, 2H), 2.25 – 2.16 (m, 2H). ^{13}C NMR (101 MHz, Chloroform-*d*) δ 158.54, 141.57, 139.46, 138.22, 134.28, 130.30, 130.26, 129.22, 127.13, 125.80, 114.69, 113.49, 55.30, 35.20, 32.62.

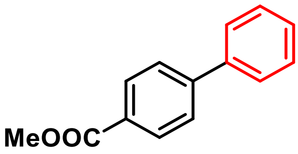

3fa

Methyl [1,1'-biphenyl]-4-carboxylate (3fa[15]): white solid was obtained with 73% isolated yield (method A, 0.2 mmol scale, 31.0 mg). ^{1}H NMR (400 MHz, Chloroform-*d*) δ 8.13 – 8.07 (m, 2H), 7.68 – 7.64 (m, 2H), 7.64 – 7.60 (m, 2H), 7.49 – 7.43 (m, 2H), 7.42 – 7.36 (m, 1H), 3.94 (s, 3H). ^{13}C NMR (101 MHz, Chloroform-*d*) δ 166.99, 145.59, 139.95, 130.06, 128.89, 128.82, 128.11, 127.24, 127.02, 52.12.

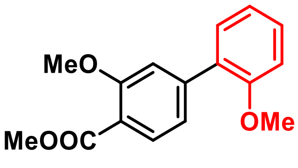

3gb

Methyl 2',3-dimethoxy-[1,1'-biphenyl]-4-carboxylate (3gb): white solid was obtained with 93% isolated yield (method A, 0.2 mmol scale, 50.6 mg). ^{1}H NMR (400 MHz, Chloroform-*d*) δ 7.86 (d, *J* = 8.0 Hz, 1H), 7.35 (m, 2H), 7.18 – 7.11 (m, 2H), 7.04 (td, *J* = 7.2, 0.8 Hz, 1H), 7.00 (d, *J* = 8.4 Hz, 1H), 3.93 (s, 3H), 3.90 (s, 3H), 3.81 (s, 3H). ^{13}C NMR (101 MHz, Chloroform-*d*) δ 166.52,

158.79, 144.16, 131.31, 130.55, 129.49, 129.38, 121.36, 120.82, 117.97, 113.37, 111.28, 55.96, 55.52, 51.91. HRMS (ESI) calcd for $C_{16}H_{16}O_4$ [M+H]+: 273.1121 found: 273.1119.

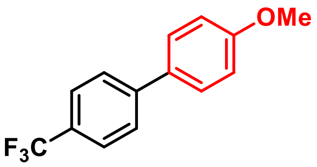

3hc

4-methoxy-4'-(trifluoromethyl)-1,1'-biphenyl (3hc[16]): white solid was obtained with 81% isolated yield (method A, 0.2 mmol scale, 40.8 mg). ^{1}H NMR (400 MHz, Chloroform-*d*) δ 7.64 (m, 4H), 7.53 (d, *J* = 8.8 Hz, 2H), 6.99 (d, *J* = 8.8 Hz, 2H), 3.84 (s, 3H). ^{13}C NMR (101 MHz, Chloroform-*d*) δ 159.80, 144.23, 132.10, 129.10, 128.78, 128.46, 128.31, 128.13, 126.82, 125.70, 125.66, 125.62, 125.58, 123.01, 114.38, 55.31. ^{19}F NMR (377 MHz, Chloroform-*d*) δ -62.25.

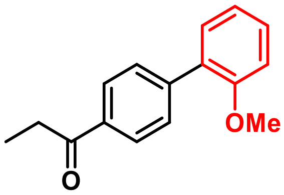

3ib

1-(2'-methoxy-[1,1'-biphenyl]-4-yl)propan-1-one (3ib[17]): white solid was obtained with 77% isolated yield (method A, 0.2 mmol scale, 37.0 mg). ^{1}H NMR (400 MHz, Chloroform-*d*) δ 8.07 – 7.93 (m, 2H), 7.67 – 7.59 (m, 2H), 7.42 – 7.28 (m, 2H), 7.12 – 6.96 (m, 2H), 3.81 (s, 3H), 3.03 (q, *J* = 7.2 Hz, 2H), 1.24 (t, *J* = 7.2 Hz, 3H). ^{13}C NMR (101 MHz, Chloroform-*d*) δ 200.52, 156.35, 143.25, 135.15, 130.65, 129.63, 129.37, 127.68, 120.87, 111.20, 55.47, 31.72, 8.28.

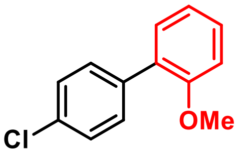

3jb

4'-chloro-2-methoxy-1,1'-biphenyl (3jb[18]): white solid was obtained with 60% isolated yield (method A, 0.2 mmol scale, 26.2 mg). ^{1}H NMR (400 MHz, Chloroform-*d*) δ 7.49 – 7.43 (m, 2H), 7.39 – 7.30 (m, 3H), 7.28 (dd, *J* = 7.6, 1.6 Hz, 1H), 7.02 (td, *J* = 7.2, 0.8 Hz, 1H), 6.98 (d, *J* = 8.4 Hz, 1H), 3.80 (s, 3H).^{13}C NMR (101 MHz, Chloroform-*d*) δ 156.29, 136.88, 132.81, 130.81, 130.62, 129.35, 128.95, 128.12, 120.86, 111.19, 55.49.

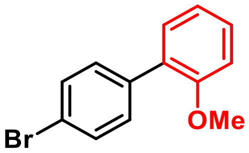

3kb, X= I

4'-bromo-2-methoxy-1,1'-biphenyl (3kb): white solid was obtained with 55% isolated yield

(method A, 0.2 mmol scale, 28.8 mg). ^{1}H NMR (400 MHz, Chloroform-*d*) δ 7.55 – 7.50 (m, 2H), 7.43 – 7.37 (m, 2H), 7.36 – 7.31 (m, 1H), 7.30 – 7.26 (m, 1H), 7.02 (s, 1H), 6.99 (s, 1H), 3.81 (s, 3H).^{13}C NMR (101 MHz, Chloroform-*d*) δ 156.24, 137.36, 131.17, 131.07, 130.56, 129.35, 128.99, 121.04, 120.88, 111.19, 55.50. HRMS (ESI) calcd for $C_{13}H_{11}BrO$ [M+H]+: 263.0066, 265.0046 found: 263.0068, 265.0051.

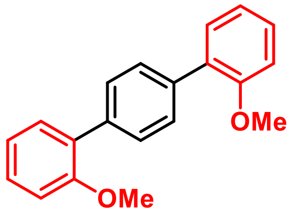

3lb

2,2''-dimethoxy-1,1':4',1''-terphenyl (3lb[18]**)**: white solid was obtained with 33% isolated yield (24% mono-substituted cross-coupling, method A, 0.2 mmol scale, 19.2 mg 3lb, 12.6 mg 3kb). ^{1}H NMR (400 MHz, Chloroform-*d*) δ 7.59 (s, 4H), 7.39 (dd, *J* = 7.2, 1.2 Hz, 2H), 7.33 (m, 2H), 7.08 – 6.97 (m, 4H), 3.83 (s, 6H). ^{13}C NMR (101 MHz, Chloroform-*d*) δ 156.50, 137.00, 130.88, 130.42, 129.13, 128.50, 120.79, 111.10, 55.49.

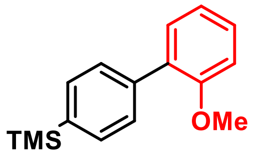

3mb

(2'-methoxy-[1,1'-biphenyl]-4-yl)trimethylsilane (3mb[19]**)**: white liquid was obtained with 57% isolated yield (method A, 0.2 mmol scale, 29.2 mg). ^{1}H NMR (400 MHz, Chloroform-*d*) δ 7.61 – 7.48 (m, 4H), 7.35 – 7.28 (m, 2H), 7.07 – 6.94 (m, 2H), 3.81 (s, 3H), 0.29 (s, 9H). ^{13}C NMR (101 MHz, Chloroform-*d*) δ 156.42, 138.88, 138.71, 133.04, 130.85, 130.52, 128.78, 128.61, 120.78, 111.05, 55.48, -1.07.

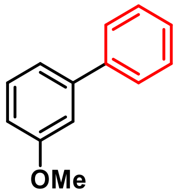

3na

3-methoxy-1,1'-biphenyl (3na[20]**)**: white solid was obtained with 53% isolated yield (method A, 0.2 mmol scale, 19.5 mg). ^{1}H NMR (400 MHz, Chloroform-*d*) δ 7.61 – 7.56 (m, 2H), 7.46 – 7.40 (m, 2H), 7.38 – 7.31 (m, 2H), 7.18 (m, 1H), 7.14 – 7.11 (m, 1H), 6.89 (dd, *J* = 8.0, 2.4 Hz, 1H) , 3.87 (s, 3H).^{13}C NMR (101 MHz, Chloroform-*d*) δ 159.87, 142.74, 141.06, 129.73, 128.71, 127.39, 127.17, 119.66, 55.27.

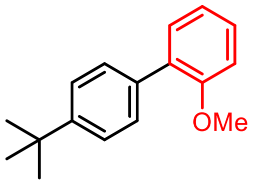

3ob

4'-(tert-butyl)-2-methoxy-1,1'-biphenyl (3ob): white solid was obtained with 65% isolated yield (method A, 0.2 mmol scale, 31.2 mg). ^{1}H NMR (400 MHz, Chloroform-*d*) δ 7.52 – 7.41 (m, 4H), 7.34 – 7.28 (m, 2H), 7.04 – 6.94 (m, 2H), 3.81 (s, 3H), 1.36 (s, 9H).^{13}C NMR (101 MHz, Chloroform-*d*) δ 156.44, 149.62, 135.44, 130.84, 130.44, 129.09, 128.31, 124.94, 120.75, 111.03, 55.47, 34.49, 31.37. HRMS (ESI) calcd for $C_{17}H_{20}O$ [M+H]+: 241.1587 found: 241.1583.

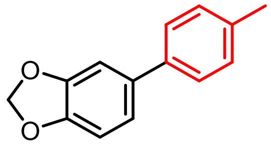

3pe

5-(p-tolyl)benzo[d][1,3]dioxole (3pe[20]): white liquid was obtained with 59% isolated yield (method A, 0.2 mmol scale, 25.0 mg). ^{1}H NMR (400 MHz, Chloroform-*d*) δ 7.41 (d, *J* = 8.0 Hz, 2H), 7.21 (d, *J* = 7.6 Hz, 2H), 7.09 – 7.00 (m, 2H), 6.86 (d, *J* = 8.0 Hz, 1H), 5.97 (s, 2H), 2.37 (s, 3H).^{13}C NMR (101 MHz, Chloroform-*d*) δ 147.99, 146.75, 138.00, 136.63, 135.50, 129.41, 126.68, 120.31, 108.49, 107.49, 101.04, 21.03.

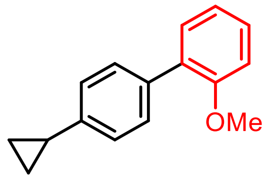

3qb

4'-cyclopropyl-2-methoxy-1,1'-biphenyl (3qb): white liquid was obtained with 65% isolated yield (method A, 0.2 mmol scale, 29.1 mg). ^{1}H NMR (400 MHz, Chloroform-*d*) δ 7.43 (d, *J* = 8.0 Hz, 2H), 7.33 – 7.26 (m, 2H), 7.11 (d, *J* = 8.0 Hz, 2H), 7.01 (td, *J* = 7.6, 0.8 Hz, 1H), 6.97 (d, *J* = 8.8 Hz, 1H), 3.80 (s, 3H), 1.92 (tt, *J* = 8.5, 5.1 Hz, 1H), 1.02 – 0.89 (m, 2H), 0.78 – 0.62 (m, 2H). ^{13}C NMR (101 MHz, Chloroform-*d*) δ 156.39, 142.69, 135.48, 130.71, 130.52, 129.36, 128.31, 120.73, 111.05, 55.47, 15.17, 9.26. HRMS (ESI) calcd for $C_{16}H_{16}O$ [M+H]+: 225.1274 found: 225.1268.

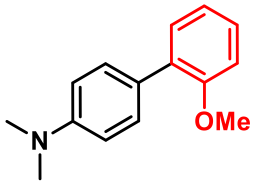

3rb

2'-methoxy-N,N-dimethyl-[1,1'-biphenyl]-4-amine (3rb[18]): white solid was obtained with 56% isolated yield from iodine, 0% from bromide (method A, 0.2 mmol scale, 25.4 mg). ^{1}H NMR (400

MHz, Chloroform-*d*) δ 7.48 – 7.42 (m, 2H), 7.31 (dd, *J* = 7.6, 2.0 Hz, 1H), 7.27 – 7.22 (m, 1H), 7.00 (td, *J* = 7.6, 1.2 Hz, 1H), 6.95 (dd, *J* = 8.4, 1.2 Hz, 1H), 6.81 – 6.76 (m, 2H), 3.80 (s, 3H), 2.97 (s, 6H).^{13}C NMR (101 MHz, Chloroform-*d*) δ 156.44, 149.54, 130.76, 130.42, 130.14, 127.51, 126.47, 120.73, 112.14, 111.06, 55.46, 40.58.

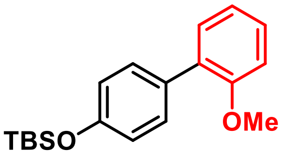

3sb

tert-butyl((2'-methoxy-[1,1'-biphenyl]-4-yl)oxy)dimethylsilane (3sb): white solid was obtained with 59% isolated yield from iodine, 46% from bromide (method A, 0.2 mmol scale, 37.1 mg from iodine, 20.9 mg from bromide). ^{1}H NMR (400 MHz, Chloroform-*d*) δ 7.45 – 7.34 (m, 2H), 7.32 – 7.24 (m, 2H), 7.00 (td, *J* = 7.6, 0.8 Hz, 1H), 6.96 (d, *J* = 8.0 Hz, 1H), 6.90 – 6.81 (m, 2H), 3.80 (s, 3H), 1.00 (s, 9H), 0.23 (s, 6H).^{13}C NMR (101 MHz, Chloroform-*d*) δ 156.34, 154.67, 131.29, 130.69, 130.49, 130.33, 128.08, 120.75, 119.45, 111.12, 55.47, 25.67, 18.17, -4.38. HRMS (ESI) calcd for $C_{19}H_{26}O_2Si$ [M+H]+: 315.1775 found: 315.1773.

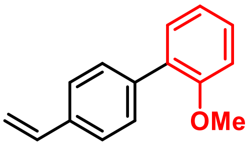

3tb

2-methoxy-4'-vinyl-1,1'-biphenyl (3tb): white solid was obtained with 62% isolated yield (method A, 0.2 mmol scale, 26.1 mg). ^{1}H NMR (400 MHz, Chloroform-*d*) δ 7.54 – 7.43 (m, 4H), 7.35 – 7.29 (m, 2H), 7.05 – 7.00 (m, 1H), 6.98 (d, J = 8.8 Hz, 1H), 6.75 (dd, *J* = 17.6, 11.2 Hz, 1H), 5.78 (d, *J* = 17.6 Hz, 1H), 5.25 (d, *J* = 11.2 Hz, 1H), 3.81 (s, 3H). ^{13}C NMR (101 MHz, Chloroform-*d*) δ 156.44, 138.00, 136.61, 136.17, 130.67, 130.24, 129.64, 128.63, 125.84, 120.81, 113.63, 111.17, 55.50. HRMS (ESI) calcd for $C_{15}H_{14}O$ [M+H]+: 211.1117 found: 211.1123.

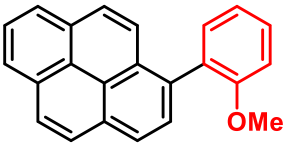

3ub

1-(2-methoxyphenyl)pyrene (3ub): white solid was obtained with 85% isolated yield (method A, 0.2 mmol scale, 52.4 mg). ^{1}H NMR (400 MHz, Chloroform-*d*) δ 8.20 (d, *J* = 8.0 Hz, 1H), 8.17 – 8.09 (m, 2H), 8.09 – 8.02 (m, 2H), 7.99 – 7.92 (m, 3H), 7.85 (d, *J* = 9.2 Hz, 1H), 7.49 – 7.43 (m, 1H), 7.40 (dd, *J* = 7.2, 1.6 Hz, 1H), 7.13 (td, *J* = 7.6, 1.2 Hz, 1H), 7.08 (dd, *J* = 8.4, 0.8 Hz, 1H), 3.68 (s, 3H).^{13}C NMR (101 MHz, Chloroform-*d*) δ 157.25, 134.28, 132.37, 131.35, 130.97, 130.60, 129.83, 129.18, 129.06, 128.03, 127.43, 127.20, 127.00, 125.85, 125.79, 124.91, 124.82, 124.75, 124.69, 124.46, 120.59, 111.08, 55.53. HRMS (ESI) calcd for $C_{23}H_{16}O$ [M+H]+: 309.1274 found: 309.1274.

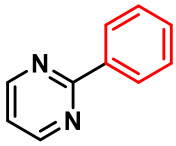

3va

2-phenylpyrimidine (3va[21]): white solid was obtained with 89% isolated yield (method A, 0.2 mmol scale, 27.8 mg). ^{1}H NMR (400 MHz, Chloroform-*d*) δ 8.81 (d, *J* = 4.8 Hz, 2H), 8.47 – 8.40 (m, 2H), 7.54 – 7.45 (m, 3H), 7.18 (t, *J* = 4.8 Hz, 1H). ^{13}C NMR (101 MHz, Chloroform-*d*) δ 164.68, 157.21, 137.48, 130.75, 128.58, 128.08, 119.06.

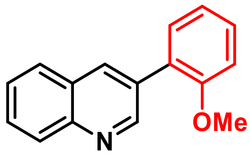

3wb

3-phenylquinoline (3wb[22]): white liquid was obtained with 60% isolated yield (method A, 0.2 mmol scale, 28.2 mg). ^{1}H NMR (400 MHz, Chloroform-*d*) δ 9.12 (s, 1H), 8.24 (s, 1H), 8.13 (d, *J* = 8.4 Hz, 1H), 7.83 (d, *J* = 8.2 Hz, 1H), 7.75 – 7.65 (t, *J* = 7.2 Hz, 1H), 7.60 – 7.46 (t, *J* = 7.2 Hz, 1H), 7.45 – 7.35 (m, 2H), 7.09 (t, *J* = 7.2 Hz, 1H), 7.02 (d, *J* = 8.4 Hz, 1H), 3.82 (s, 3H).^{13}C NMR (101 MHz, Chloroform-*d*) δ 156.61, 151.93, 146.74, 135.41, 131.59, 130.86, 129.56, 129.10, 129.00, 127.87, 126.99, 126.50, 121.06, 111.19, 55.43.

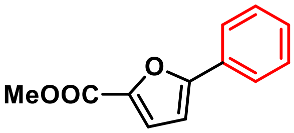

3xa

methyl 5-phenylfuran-2-carboxylate (3xa): white solid was obtained with 80% isolated yield (method A, 0.2 mmol scale, 32.3 mg). ^{1}H NMR (400 MHz, Chloroform-*d*) δ 7.86 – 7.71 (m, 2H), 7.45 – 7.38 (m, 2H), 7.37 – 7.31 (m, 1H), 7.25 (d, *J* = 3.6 Hz, 1H), 6.74 (d, *J* = 3.6 Hz, 1H), 3.91 (s, 3H).^{13}C NMR (101 MHz, Chloroform-*d*) δ 159.19, 157.52, 143.49, 129.41, 128.90, 128.77, 124.78, 120.03, 106.81, 51.83. HRMS (ESI) calcd for $C_{12}H_{10}O_3$ [M+H]+: 203.0703 found: 203.0701.

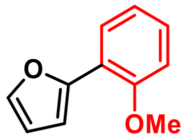

3yb

2-(2-methoxyphenyl)furan (3yb[23]): white liquid was obtained with 70% isolated yield (method A, 0.2 mmol scale, 24.4 mg). ^{1}H NMR (400 MHz, Chloroform-*d*) δ 7.85 (dd, *J* = 8.0, 2.0 Hz, 1H), 7.46 (dd, *J* = 2.0, 0.8 Hz, 1H), 7.27 – 7.20 (m, 1H), 7.02 (td, *J* = 7.6, 1.2 Hz, 1H), 6.98 – 6.92 (m, 1H), 6.49 (dd, *J* = 3.2, 1.6 Hz, 1H), 3.93 (s, 3H).^{13}C NMR (101 MHz, Chloroform-*d*) δ 155.23, 150.20, 141.06, 127.97, 125.91, 120.67, 119.82, 111.60, 110.89, 109.77, 55.32.

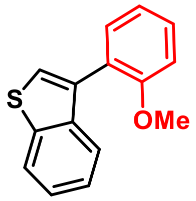

3zb

3-(2-methoxyphenyl)benzo[b]thiophene (3zb[18]): white liquid was obtained with 74% isolated yield (method A, 0.2 mmol scale, 35.5 mg). ^{1}H NMR (400 MHz, Chloroform-*d*) δ 7.91 – 7.85 (m, 1H), 7.64 – 7.59 (m, 1H), 7.41 (s, 1H), 7.42 – 7.33 (m, 1H), 7.36 – 7.27 (m, 1H), 7.09 – 6.98 (m, 1H), 3.75 (s, 2H).^{13}C NMR (101 MHz, Chloroform-*d*) δ 157.10, 139.79, 138.64, 134.23, 131.44, 129.09, 124.66, 124.55, 124.03, 123.85, 123.55, 122.58, 120.57, 111.12, 55.42.

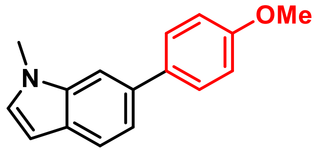

3a1c

6-(4-methoxyphenyl)-1-methyl-1H-indole (3a1c): white solid was obtained with 44% isolated yield (method A, 0.2 mmol scale, 20.9 mg). ^{1}H NMR (400 MHz, Chloroform-*d*) δ 7.78 (s, 1H), 7.57 (d, J = 8.4 Hz, 2H), 7.45 – 7.33 (m, 2H), 7.06 (d, J = 3.2 Hz, 1H), 6.98 (d, J = 8.8 Hz, 2H), 6.51 (d, J = 2.8 Hz, 1H), 3.83 (s, 1H), 3.79 (s, 3H).^{13}C NMR (101 MHz, Chloroform-*d*) δ 158.37, 135.90, 135.22, 132.46, 129.37, 128.90, 128.28, 121.14, 118.85, 114.04, 109.34, 101.11, 55.32, 32.89. HRMS (ESI) calcd for $C_{16}H_{15}NO$ [M+H]+: 238.1226 found: 238.1220.

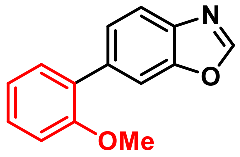

3a2b

6-(2-methoxyphenyl)benzo[d]oxazole (3a2b): white solid was obtained with 56% isolated yield (method A, 0.2 mmol scale, 25.2 mg). ^{1}H NMR (400 MHz, Chloroform-*d*) δ 8.11 (s, 1H), 7.81 (d, J = 8.0 Hz, 1H), 7.7 (d, J = 1.2 Hz, 1H), 7.53 (dd, J = 8.0, 1.2 Hz, 1H), 7.36 (m, 2H), 7.06 (td, J = 7.6, 1.2 Hz, 1H), 7.03 – 7.00 (m, 1H), 3.83 (s, 3H).^{13}C NMR (101 MHz, Chloroform-*d*) δ 156.30, 152.70, 149.89, 138.90, 136.40, 131.09, 129.86, 128.97, 126.46, 120.88, 119.70, 111.96, 111.18, 55.51. HRMS (ESI) calcd for $C_{14}H_{11}NO_2$ [M+H]+: 226.0863 found: 226.0859.

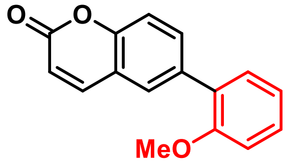

3a3b

6-(2-methoxyphenyl)-2H-chromen-2-one (3a3b): white solid was obtained with 40% isolated yield (method A, 0.2 mmol scale, 20.2 mg).^{1}H NMR (400 MHz, Chloroform-*d*) δ 7.73 (d, J = 9.6

Hz, 1H), 7.69 (dd, J = 8.4, 2.0 Hz, 1H), 7.63 (d, J = 2.0 Hz, 1H), 7.36 (m, 2H), 7.31 (dd, J = 7.2, 1.6 Hz, 1H), 7.09 – 6.95 (m, 2H), 6.43 (d, J = 9.2 Hz, 1H), 3.82 (s, 3H).^{13}C NMR (101 MHz, Chloroform-d) δ 160.88, 156.20, 152.90, 143.68, 134.90, 133.25, 130.56, 129.19, 128.54, 128.46, 120.90, 118.39, 116.42, 116.29, 111.15, 55.44. HRMS (ESI) calcd for $C_{16}H_{12}O_3$ [M+H]+: 253.0859 found: 253.0852.

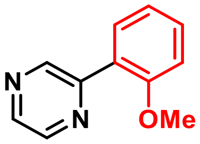

3a4b

2-(2-methoxyphenyl)pyrazine (3a4b[24]): white solid was obtained with 91% isolated yield (method A, 0.2 mmol scale, 33.9 mg). ^{1}H NMR (400 MHz, Chloroform-d) δ 9.15 (d, J = 1.6 Hz, 1H), 8.66 – 8.62 (m, 1H), 8.45 (d, J = 2.4 Hz, 1H), 7.82 (dd, J = 7.6, 2.0 Hz, 1H), 7.43 (m, 1H), 7.11 (td, J = 7.6, 1.2 Hz, 1H), 7.03 (d, J = 8.0 Hz, 1H), 3.89 (s, 3H).^{13}C NMR (101 MHz, Chloroform-d) δ 156.96, 151.84, 146.41, 144.03, 142.00, 131.09, 130.96, 125.65, 121.17, 111.26, 55.48.

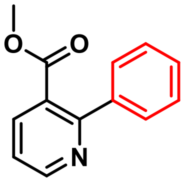

3a5a

methyl 2-phenylnicotinate (3a5a): white solid was obtained with 99% isolated yield (method A, 0.2 mmol scale, 42.2 mg). ^{1}H NMR (400 MHz, Chloroform-d) δ 8.77 (dd, J = 4.8, 1.6 Hz, 1H), 8.10 (dd, J = 7.6, 1.6 Hz, 1H), 7.58 – 7.51 (m, 2H), 7.47 – 7.39 (m, 3H), 7.33 (dd, J = 8.0, 4.8 Hz, 1H), 3.69 (s, 3H). ^{13}C NMR (101 MHz, Chloroform-d) δ 168.49, 158.69, 151.24, 139.89, 137.81, 128.65, 128.40, 128.10, 126.87, 121.49, 52.30. HRMS (ESI) calcd for $C_{13}H_{11}NO_2$ [M+H]+: 214.0863 found: 214.0855.

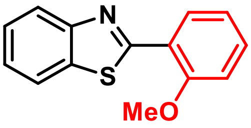

3a6b

2-(2-methoxyphenyl)benzo[d]thiazole (3a6b[25]): white liquid was obtained with 70% isolated yield (method A, 0.2 mmol scale, 33.8 mg). ^{1}H NMR (400 MHz, Chloroform-d) δ 8.53 (dd, J = 8.0, 1.6 Hz, 1H), 8.09 (d, J = 8.0 Hz, 1H), 7.92 (d, J = 7.6 Hz, 1H), 7.47 (m, 2H), 7.36 (t, J = 7.2 Hz, 1H), 7.13 (t, J = 7.6 Hz, 1H), 7.05 (d, J = 8.4 Hz, 1H), 4.04 (s, 3H). ^{13}C NMR (101 MHz, Chloroform-d) δ 163.10, 157.17, 152.09, 136.06, 131.73, 129.48, 125.85, 124.54, 122.72, 122.21, 121.16, 121.11, 111.61, 55.65.

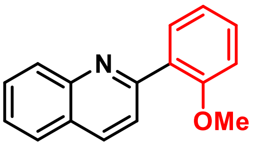

3a7b

2-(2-methoxyphenyl)quinoline (3a7b[26]): white liquid was obtained with 34% isolated yield (method A, 0.2 mmol scale, 16.0 mg). ^{1}H NMR (400 MHz, Chloroform-*d*) δ 8.17 (d, *J* = 8.8 Hz, 1H), 8.12 (d, *J* = 8.8 Hz, 1H), 7.91 – 7.83 (m, 2H), 7.83 – 7.79 (m, 1H), 7.69 (m, 1H), 7.54 – 7.47 (m, 1H), 7.44 – 7.38 (m, 1H), 7.13 (td, *J* = 7.6, 0.8 Hz, 1H), 7.02 (d, *J* = 8.4 Hz, 1H), 3.84 (s, 3H). ^{13}C NMR (101 MHz, Chloroform-*d*) δ 157.08, 157.04, 148.20, 135.03, 131.39, 130.26, 129.58, 129.52, 129.15, 127.32, 126.95, 126.10, 123.38, 121.18, 111.30, 55.54.

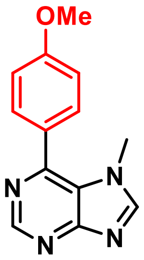

3a8c

6-(4-methoxyphenyl)-7-methyl-7H-purine (3a8c): white solid was obtained with 73% isolated yield (method A, 0.2 mmol scale, 35.1 mg). ^{1}H NMR (400 MHz, DMSO-d_6) δ 8.78 (s, 1H), 8.75 – 8.68 (d, *J* = 8.8 Hz, 2H), 8.45 (s, 1H), 7.05 – 6.98 (d, *J* = 8.8 Hz, 2H), 3.73 (s, 6H). ^{13}C NMR (101 MHz, DMSO-d_6) δ 162.05, 152.94, 152.63, 152.15, 147.05, 131.56, 130.03, 128.37, 114.55, 55.83, 30.07. HRMS (ESI) calcd for $C_{13}H_{14}N_4O$ [M+H]+: 241.1084 found: 241.1080.

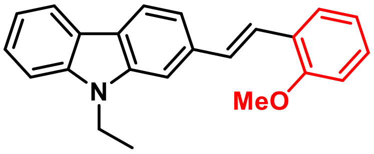

3a9b

(E)-9-ethyl-2-(2-methoxystyryl)-9H-carbazole (3a9b): white solid was obtained with 80% isolated yield (method A, 0.2 mmol scale, 52.4 mg). ^{1}H NMR (400 MHz, Chloroform-*d*) δ 8.24 (d, *J* = 1.6 Hz, 1H), 8.12 (d, *J* = 7.6 Hz, 1H), 7.66 (m, 2H), 7.52 (d, *J* = 16.8 Hz, 1H), 7.48 – 7.41 (m, 1H), 7.39 – 7.27 (m, 3H), 7.26 – 7.18 (m, 2H), 6.98 (t, *J* = 7.2 Hz, 1H), 6.89 (d, *J* = 7.6 Hz, 1H), 4.30 (q, *J* = 7.2 Hz, 2H), 3.89 (s, 3H), 1.39 (t, *J* = 7.2 Hz, 3H). ^{13}C NMR (101 MHz, Chloroform-*d*) δ 156.62, 140.24, 139.54, 130.05, 129.11, 127.96, 126.99, 125.99, 125.65, 124.58, 123.17, 122.96, 120.72, 120.55, 120.47, 118.86, 118.65, 110.84, 108.52, 108.48, 55.47, 37.51, 13.78. HRMS (ESI) calcd for $C_{23}H_{21}NO$ [M+H]+: 328.1696 found: 328.1689.

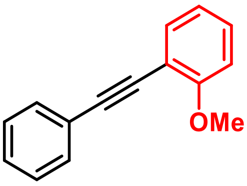

3a10b

1-methoxy-2-(phenylethynyl)benzene (3a10b[27]): white solid was obtained with 40% isolated yield (method A, 0.2 mmol scale, 16.7 mg). ^{1}H NMR (400 MHz, Chloroform-*d*) δ 7.60 – 7.54 (m, 2H), 7.50 (d, *J* = 7.2 Hz, 2H), 7.39 – 7.30 (m, 4H), 6.98 – 6.87 (m, 2H), 3.92 (s, 3H). ^{13}C NMR (101

MHz, Chloroform-*d*) δ 159.86, 133.55, 131.64, 129.74, 128.21, 128.08, 123.50, 120.45, 112.37, 110.63, 93.39, 85.66, 55.81.

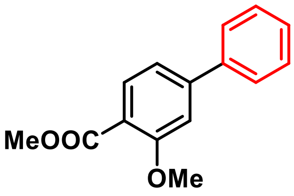

3ga

methyl 3-methoxy-[1,1'-biphenyl]-4-carboxylate (3ga): white solid was obtained with 87% isolated yield (method A, 0.2 mmol scale, 42.1 mg). ^{1}H NMR (400 MHz, Chloroform-*d*) δ 7.89 (d, *J* = 8.0 Hz, 1H), 7.62 – 7.57 (m, 2H), 7.48 – 7.42 (m, 2H), 7.42 – 7.36 (m, 1H), 7.19 (dd, *J* = 8.0, 1.6 Hz, 1H), 7.16 (d, *J* = 1.6 Hz, 1H), 3.97 (s, 3H), 3.90 (s, 3H).^{13}C NMR (101 MHz, Chloroform-*d*) δ 166.42, 159.50, 146.68, 140.10, 132.22, 128.82, 128.17, 127.19, 118.93, 118.40, 110.75, 55.99, 51.95. HRMS (ESI) calcd for $C_{15}H_{14}O_3$ [M+H]+: 243.1016 found: 243.1014.

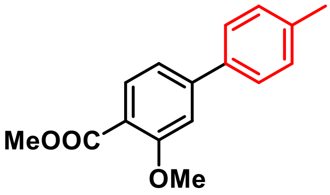

3gf

methyl 3-methoxy-4'-methyl-[1,1'-biphenyl]-4-carboxylate (3gf): white solid was obtained with 84% isolated yield (method A, 0.2 mmol scale, 43.0 mg). ^{1}H NMR (400 MHz, Chloroform-*d*) δ 7.87 (d, *J* = 8.0 Hz, 1H), 7.50 (d, *J* = 8.4 Hz, 2H), 7.26 (d, *J* = 8.0 Hz, 2H), 7.18 (dd, *J* = 8.0, 1.6 Hz, 1H), 7.15 (d, *J* = 1.6 Hz, 1H), 3.96 (s, 3H), 3.90 (s, 3H), 2.40 (s, 3H). ^{13}C NMR (101 MHz, Chloroform-*d*) δ 166.44, 159.53, 146.64, 138.15, 137.18, 132.21, 129.54, 127.01, 118.71, 118.07, 110.49, 55.97, 51.91, 21.09. HRMS (ESI) calcd for $C_{16}H_{16}O_3$ [M+H]+: 257.1172 found: 257.1170.

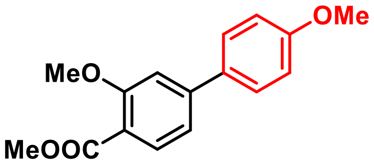

3gc

methyl 3,4'-dimethoxy-[1,1'-biphenyl]-4-carboxylate (3gc): white solid was obtained with 99% isolated yield (method A, 0.2 mmol scale, 53.9 mg). ^{1}H NMR (400 MHz, Chloroform-*d*) δ 7.87 (d, *J* = 8.0 Hz, 1H), 7.61 – 7.46 (m, 2H), 7.15 (dd, *J* = 8.0, 1.6 Hz, 1H), 7.12 (d, *J* = 1.6 Hz, 1H), 7.03 – 6.93 (m, 2H), 3.97 (s, 3H), 3.90 (s, 3H), 3.85 (s, 3H).^{13}C NMR (101 MHz, Chloroform-*d*) δ 166.41, 159.77, 159.56, 146.28, 132.41, 132.26, 128.26, 118.39, 117.63, 114.20, 110.12, 55.93, 55.27, 51.89. HRMS (ESI) calcd for $C_{16}H_{16}O_4$ [M+H]+: 273.1121 found: 273.1119.

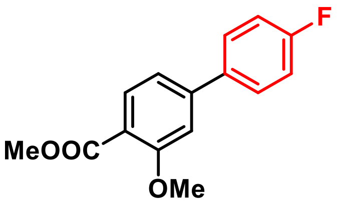

3gd

methyl 4'-fluoro-3-methoxy-[1,1'-biphenyl]-4-carboxylate (3gd): white solid was obtained with 85% isolated yield (method A, 0.2 mmol scale, 44.2 mg). ^{1}H NMR (400 MHz, Chloroform-*d*) δ 7.88 (d, *J* = 8.0 Hz, 1H), 7.60 – 7.52 (m, 2H), 7.18 – 7.09 (m, 4H), 3.97 (s, 3H), 3.91 (s, 3H). ^{13}C NMR (101 MHz, Chloroform-*d*) δ 166.36, 164.10, 161.64, 159.52, 145.64, 136.22, 136.19, 132.31, 128.90, 128.82, 118.78, 118.40, 115.88, 115.66, 110.55, 56.01, 52.28, 52.01. ^{19}F NMR (377 MHz, Chloroform-*d*) δ -114.97. HRMS (ESI) calcd for $C_{15}H_{13}FO_3$ [M+H]+: 261.0922 found: 261.0919.

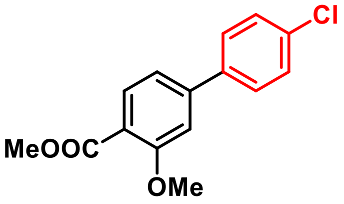

3gg

methyl 4'-chloro-3-methoxy-[1,1'-biphenyl]-4-carboxylate (3gg): white solid was obtained with 84% isolated yield (method A, 0.2 mmol scale, 46.4 mg). ^{1}H NMR (400 MHz, Chloroform-*d*) δ 7.88 (d, *J* = 8.0 Hz, 2H), 7.55 – 7.49 (m, 2H), 7.45 – 7.39 (m, 2H), 7.17 – 7.13 (m, 1H), 7.11 (d, *J* = 1.6 Hz, 1H), 3.97 (s, 3H), 3.91 (s, 3H). ^{13}C NMR (101 MHz, Chloroform-*d*) δ 166.33, 159.54, 145.38, 138.53, 134.34, 132.36, 129.02, 128.47, 118.78, 118.74, 110.53, 56.05, 52.06. HRMS (ESI) calcd for $C_{15}H_{13}ClO_3$ [M+H]+: 277.0622 found: 277.0626.

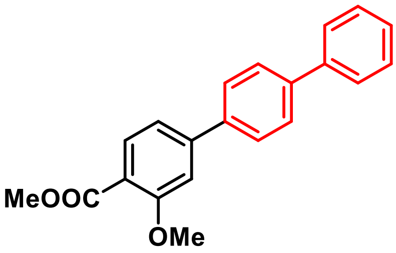

3gh

methyl 3-methoxy-[1,1':4',1''-terphenyl]-4-carboxylate (3gh): white solid was obtained with 63% isolated yield (method A, 0.2 mmol scale, 40.1 mg). ^{1}H NMR (400 MHz, Chloroform-*d*) δ 7.90 (d, *J* = 8.0 Hz, 1H), 7.68 (s, 4H), 7.65 – 7.59 (m, 2H), 7.46 (t, *J* = 7.5 Hz, 2H), 7.40 – 7.33 (m, 1H), 7.27 – 7.11 (m, 2H), 3.98 (s, 3H), 3.91 (s, 3H). ^{13}C NMR (101 MHz, Chloroform-*d*) δ 166.42, 159.57, 146.15, 141.06, 140.31, 138.91, 132.30, 128.82, 127.58, 127.54, 126.99, 118.82, 118.46, 110.57, 56.03, 51.98. HRMS (ESI) calcd for $C_{21}H_{18}O_3$ [M+H]+: 319.1329 found: 319.1326.

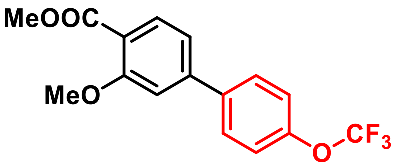

3gi

methyl 3-methoxy-4'-(trifluoromethoxy)-[1,1'-biphenyl]-4-carboxylate (3gi): white solid was obtained with 78% isolated yield (method A, 0.2 mmol scale, 50.9 mg). ^{1}H NMR (400 MHz, Chloroform-*d*) δ 7.89 (d, *J* = 8.0 Hz, 1H), 7.61 (d, *J* = 8.7 Hz, 2H), 7.31 (d, *J* = 8.1 Hz, 2H), 7.18 – 7.11 (m, 1H), 3.97 (s, 3H), 3.91 (s, 3H).^{13}C NMR (101 MHz, Chloroform-d) δ 166.35, 159.54, 149.22, 145.21, 138.86, 132.35, 128.64, 121.28, 120.42 (q, $J_{C\text{-}F}$ = 255.8 Hz), 118.91, 110.72, 56.03, 52.04. ^{19}F NMR (377 MHz, Chloroform-*d*) δ -57.80. HRMS (ESI) calcd for $C_{16}H_{13}F_3O_4$ [M+H]+: 327.0839 found: 327.0834.

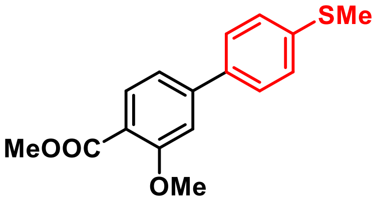

3gj

methyl 3-methoxy-4'-(methylthio)-[1,1'-biphenyl]-4-carboxylate (3gj): white solid was obtained with 88% isolated yield (method A, 0.2 mmol scale, 50.7 mg). ^{1}H NMR (400 MHz, Chloroform-*d*) δ 7.88 (d, *J* = 8.0 Hz, 1H), 7.53 (d, *J* = 8.4 Hz, 2H), 7.32 (d, *J* = 8.4 Hz, 2H), 7.17 (dd, *J* = 8.0, 1.2 Hz, 1H), 7.13 (d, *J* = 0.8 Hz, 1H), 3.97 (s, 3H), 3.90 (s, 3H), 2.52 (s, 3H). ^{13}C NMR (101 MHz, Chloroform-*d*) δ 166.36, 159.56, 145.96, 139.02, 136.59, 132.31, 127.47, 126.54, 118.54, 118.21, 110.26, 55.99, 51.97, 15.53. HRMS (ESI) calcd for $C_{16}H_{16}O_3S$ [M+H]+:289.0893 found: 289.0888.

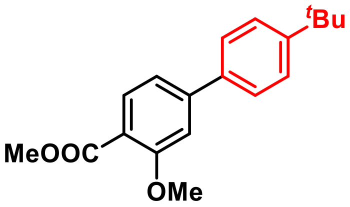

3gk

methyl 4'-(tert-butyl)-3-methoxy-[1,1'-biphenyl]-4-carboxylate (3gk): white solid was obtained with 98% isolated yield (method A, 0.2 mmol scale, 58.4 mg). ^{1}H NMR (400 MHz, Chloroform-*d*) δ 7.88 (d, *J* = 7.6 Hz, 1H), 7.57 – 7.53 (m, 1H), 7.51 – 7.46 (m, 2H), 7.22 – 7.14 (m, 2H), 3.95 (s, 3H), 3.90 (s, 3H), 1.36 (s, 9H). ^{13}C NMR (101 MHz, Chloroform-*d*) δ 166.46, 159.51, 151.38, 146.58, 137.19, 132.21, 126.86, 125.80, 118.77, 118.10, 110.57, 55.96, 51.93, 34.56, 31.24. HRMS (ESI) calcd for $C_{19}H_{22}O_3$ [M+H]+:299.1642 found: 299.1638.

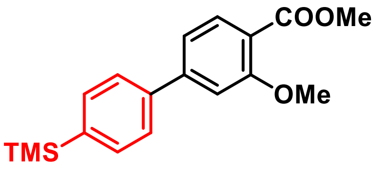

3gl

methyl 3-methoxy-4'-(trimethylsilyl)-[1,1'-biphenyl]-4-carboxylate (3gl): white liquid was obtained with 88% isolated yield (method A, 0.2 mmol scale, 55.3 mg). ^{1}H NMR (400 MHz, Chloroform-*d*) δ 7.89 (d, *J* = 8.0 Hz, 1H), 7.66 – 7.57 (m, 4H), 7.20 (dd, *J* = 8.0, 1.6 Hz, 1H), 7.17 (d, *J* = 1.6 Hz, 1H), 3.97 (s, 3H), 3.90 (s, 3H), 0.31 (s, 9H).^{13}C NMR (101 MHz, Chloroform-*d*) δ 166.46, 159.51, 146.68, 140.61, 140.46, 133.87, 133.61, 132.25, 126.51, 124.90, 118.94, 118.42, 110.74, 56.00, 52.01, -1.19. HRMS (ESI) calcd for $C_{18}H_{22}O_3Si$ [M+H]+: 315.1411 found: 315.1406.

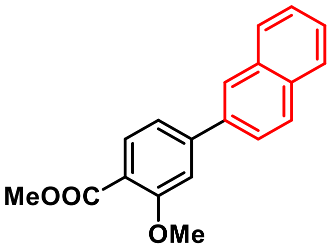

3gm

methyl 2-methoxy-4-(naphthalen-2-yl)benzoate (3gm): white solid was obtained with 84% isolated yield (method A, 0.2 mmol scale, 49.1 mg). ^{1}H NMR (400 MHz, Chloroform-*d*) δ 8.09 – 7.99 (m, 1H), 7.95 – 7.81 (m, 4H), 7.71 (dd, *J* = 8.4, 2.0 Hz, 1H), 7.56 – 7.44 (m, 2H), 7.34 – 7.25 (m, 2H), 4.00 (s, 3H), 3.91 (s, 3H).^{13}C NMR (101 MHz, Chloroform-*d*) δ 166.42, 159.58, 146.58, 137.35, 133.40, 132.93, 132.30, 128.56, 128.22, 127.61, 126.48, 126.46, 126.37, 126.19, 125.15, 119.19, 118.43, 110.93, 56.06, 51.97. HRMS (ESI) calcd for $C_{19}H_{16}O_3$ [M+H]+:292.1172 found: 293.1169.

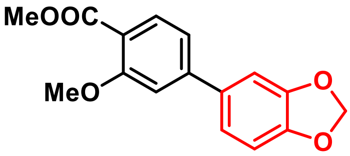

3gn

methyl 4-(benzo[d][1,3]dioxol-5-yl)-2-methoxybenzoate (3gn): white solid was obtained with 82% isolated yield (method A, 0.2 mmol scale, 16.9 mg). ^{1}H NMR (400 MHz, Chloroform-*d*) δ 7.89 (d, *J* = 8.0 Hz, 1H), 7.16 – 7.09 (m, 4H), 6.94 – 6.90 (m, 1H), 6.04 (s, 2H), 3.99 (s, 3H), 3.93 (s, 3H).^{13}C NMR (101 MHz, Chloroform-*d*) δ 166.38, 159.53, 148.17, 147.78, 146.35, 134.32, 132.25, 120.92, 118.60, 118.00, 110.38, 108.59, 107.56, 101.29, 55.97, 51.93. HRMS (ESI) calcd for $C_{16}H_{14}O_5$ [M+H]+:287.0914 found: 287.0909.

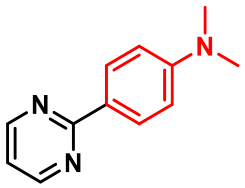

3vo

N,N-dimethyl-4-(pyrimidin-2-yl)aniline (3vo): white solid was obtained with 86% isolated yield (method A, 0.2 mmol scale, 34.3 mg). ^{1}H NMR (400 MHz, Chloroform-*d*) δ 8.69 (d, *J* = 4.8 Hz, 2H), 8.43 – 8.26 (m, 2H), 7.01 (t, *J* = 4.8 Hz, 1H), 6.91 – 6.66 (m, 2H), 3.04 (s, 6H).^{13}C NMR (101 MHz, Chloroform-*d*) δ 164.94, 156.97, 152.11, 129.32, 125.06, 117.37, 111.56, 40.17. HRMS (ESI) calcd for $C_{12}H_{13}N_3$ [M+H]+:200.1182 found: 200.1181.

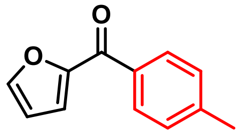

3a11f

furan-2-yl(p-tolyl)methanone (3a11f[28]): white liquid was obtained with 85% isolated yield (method A, 0.2 mmol scale, 31.6 mg). ^{1}H NMR (400 MHz, Chloroform-*d*) δ 7.89 (d, *J* = 8.0 Hz, 2H), 7.70 (d, *J* = 1.6 Hz, 1H), 7.30 (d, *J* = 8.0 Hz, 2H), 7.22 (d, *J* = 3.6 Hz, 1H), 6.59 (d, *J* = 3.2 Hz, 1H), 2.44 (s, 3H).^{13}C NMR (101 MHz, Chloroform-*d*) δ 182.26, 152.32, 146.84, 143.36, 134.49, 129.39, 129.06, 120.18, 112.07, 21.60.

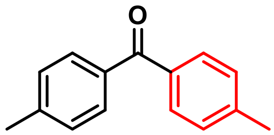

3a12f

di-p-tolylmethanone (3a12f[29]): white liquid was obtained with 84% isolated yield (method A, 0.2 mmol scale, 35.3 mg). ^{1}H NMR (400 MHz, Chloroform-*d*) δ 7.70 (d, *J* = 8.0 Hz, 4H), 7.27 (d, *J* = 8.4 Hz, 4H), 2.44 (s, 6H).^{13}C NMR (101 MHz, Chloroform-*d*) δ 196.31, 142.92, 135.14, 130.16, 128.87, 21.61.

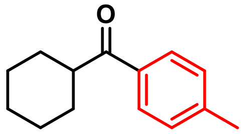

3a13f

cyclohexyl(p-tolyl)methanone (3a13f[30]): white liquid was obtained with 88% isolated yield (method A, 0.2 mmol scale, 35.6 mg). ^{1}H NMR (400 MHz, Chloroform-*d*) δ 7.85 (d, *J* = 8.0 Hz, 2H), 7.25 (d, *J* = 8.4 Hz, 2H), 3.31 – 3.18 (m, 1H), 2.40 (s, 3H), 1.86 (t, *J* = 13.6 Hz, 4H), 1.74 (d, *J* = 14.4 Hz, 1H), 1.56 – 1.23 (m, 5H).^{13}C NMR (101 MHz, Chloroform-*d*) δ 203.54, 143.41, 133.69, 129.21, 128.34, 45.44, 29.42, 25.92, 25.83, 21.55.

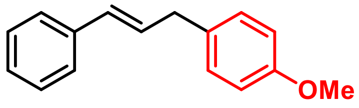

3a14c

1-cinnamyl-4-methoxybenzene (3a14c[31]): white liquid was obtained with 41% isolated yield (method A, 0.2 mmol scale, 18.4 mg). ^{1}H NMR (400 MHz, Chloroform-*d*) δ 7.35 (d, *J* = 7.2 Hz, 2H), 7.29 (t, *J* = 7.6 Hz, 2H), 7.22 – 7.12 (m, 3H), 6.85 (d, *J* = 8.8 Hz, 2H), 6.46 – 6.39 (m, 1H), 6.34 (m, 1H), 3.79 (s, 3H), 3.49 (d, *J* = 6.4 Hz, 2H).^{13}C NMR (101 MHz, Chloroform-*d*) δ 158.00, 137.48, 132.14, 130.68, 129.64, 129.58, 128.46, 127.02, 126.07, 113.85, 55.26, 38.42.

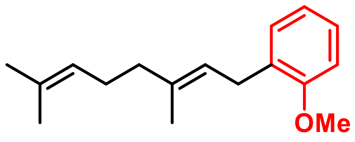

3a15b

(E)-1-(3,7-dimethylocta-2,6-dien-1-yl)-4-methoxybenzene (3a15b): white liquid was obtained with 35% isolated yield (method A, 0.2 mmol scale, 17.1 mg). ^{1}H NMR (400 MHz, Chloroform-*d*) δ 7.16 (m, 2H), 6.92 – 6.81 (m, 2H), 5.32 (m, 1H), 5.11 (m, 1H), 3.82 (s, 1H), 3.33 (d, *J* = 7.2 Hz, 2H), 2.16 – 2.07 (m, 2H), 2.08 – 1.96 (m, 2H), 1.76 – 1.66 (m, 6H), 1.63 – 1.54 (m, 3H). ^{13}C NMR (101 MHz, Chloroform-*d*) δ 157.24, 136.13, 131.36, 129.99, 129.17, 126.77, 124.37, 122.29, 120.36, 55.27, 39.77, 28.09, 26.61, 25.70, 17.68, 15.98, 15.94. HRMS (ESI) calcd for $C_{17}H_{24}O$ [M+H]+: 245.1900 found: 245.1893.

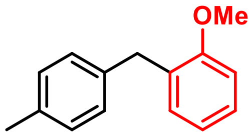

3a16b

1-methoxy-2-(4-methylbenzyl)benzene (3a16b[32]): white solid was obtained with 56% isolated yield (method A, 0.2 mmol scale, 23.8 mg). ^{1}H NMR (400 MHz, Chloroform-*d*) δ 7.21 – 7.15 (m, 1H), 7.13 – 7.02 (m, 2H), 6.89 – 6.82 (m, 2H), 3.93 (s, 2H), 3.81 (s, 3H), 2.30 (s, 3H).^{13}C NMR (101 MHz, Chloroform-*d*) δ 157.23, 137.84, 135.16, 130.18, 129.84, 128.92, 128.81, 127.26, 120.39, 110.26, 55.29, 35.32, 21.01.

3.3 Copies of product NMR Spectra

^{1}H NMR

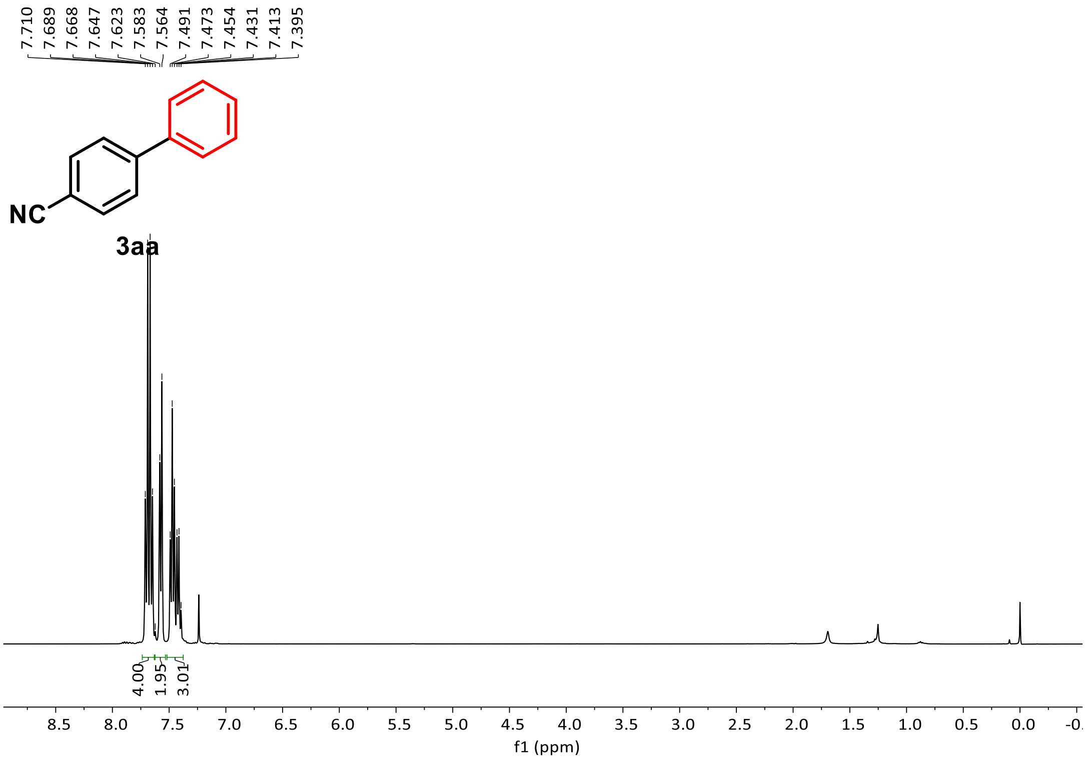

Supplementary Figure 13. ^{1}H NMR of compound **3aa** (400 MHZ, r.t., $CDCl_3$)

^{13}C NMR

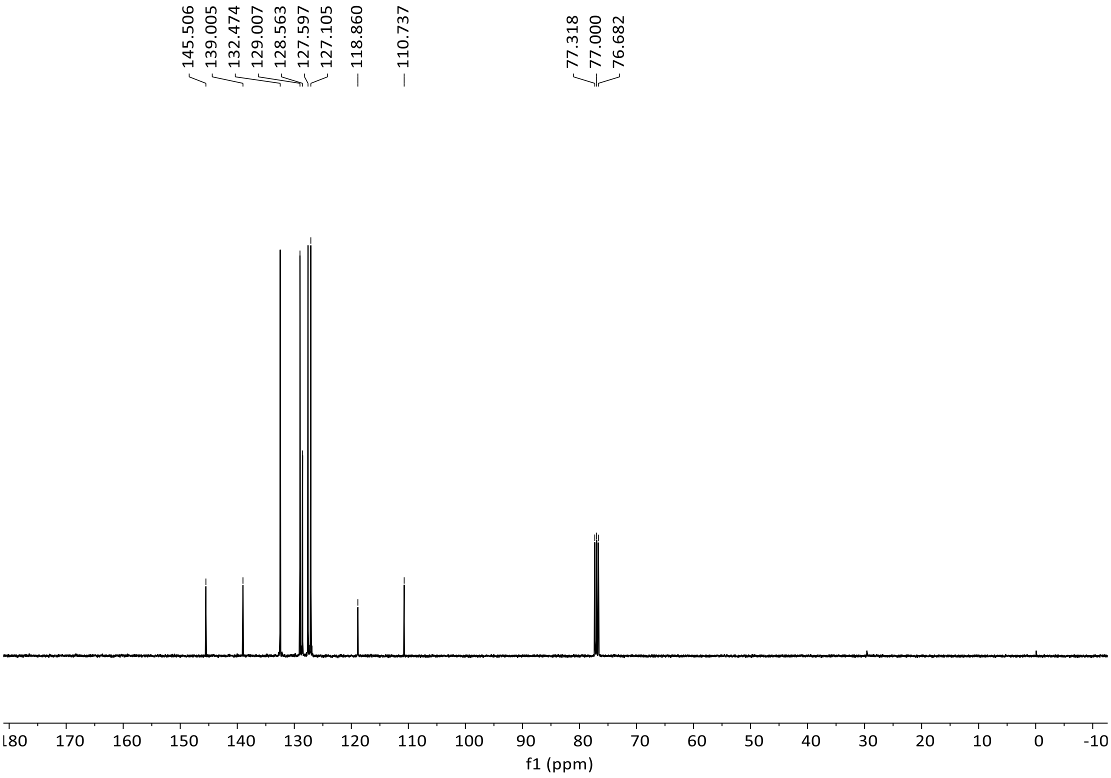

Supplementary Figure 14. ^{13}C NMR of compound **3aa** (101 MHZ, r.t., $CDCl_3$)

^{1}H NMR

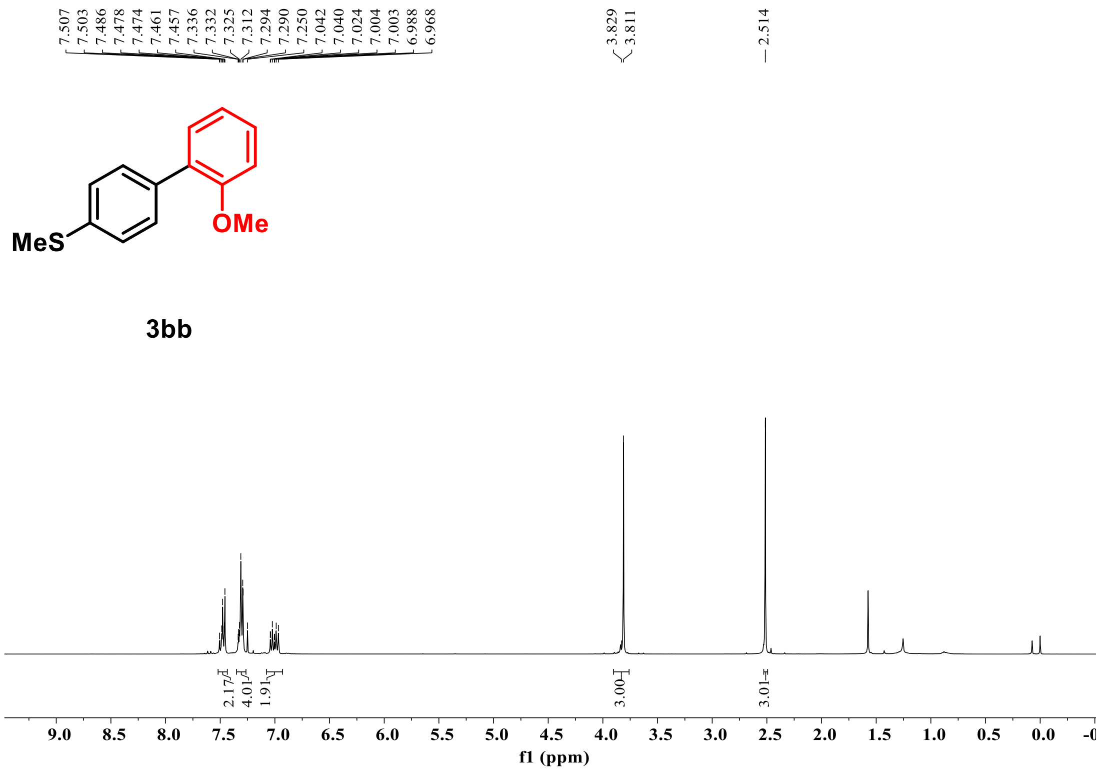

Supplementary Figure 15. ¹H NMR of compound **3bb** (400 MHZ, r.t., $CDCl_3$)

^{13}C NMR

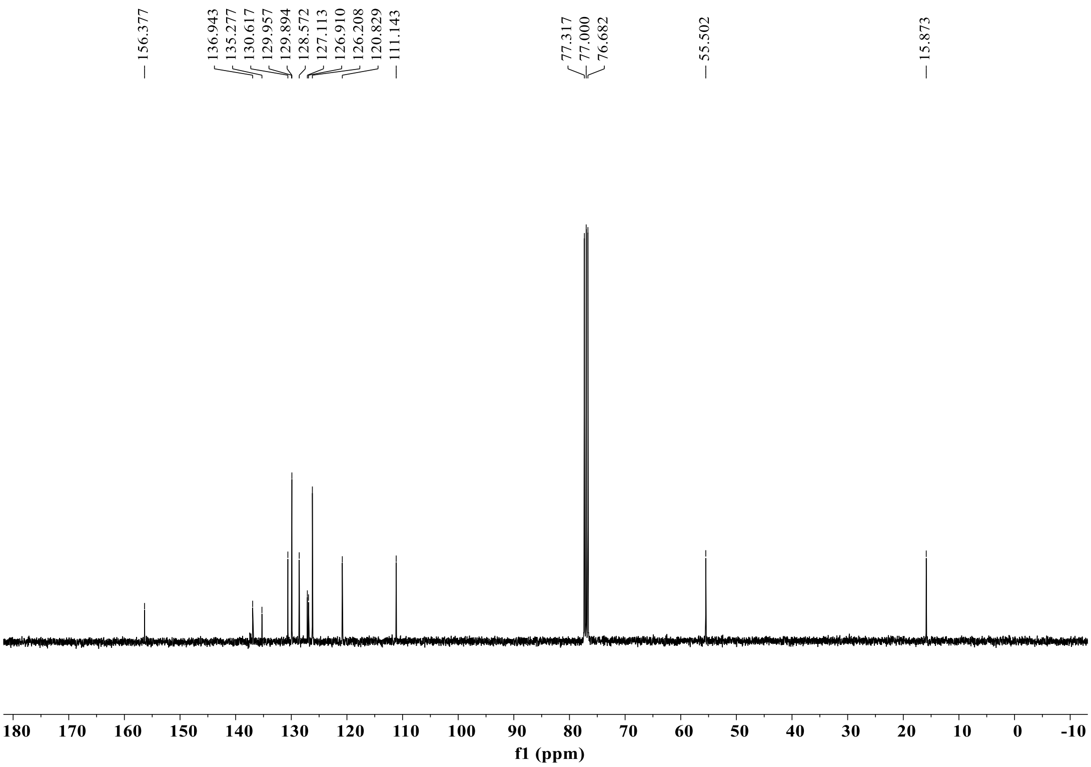

Supplementary Figure 16. ^{13}C NMR of compound **3bb** (101 MHZ, r.t., $CDCl_3$)

^{1}H NMR

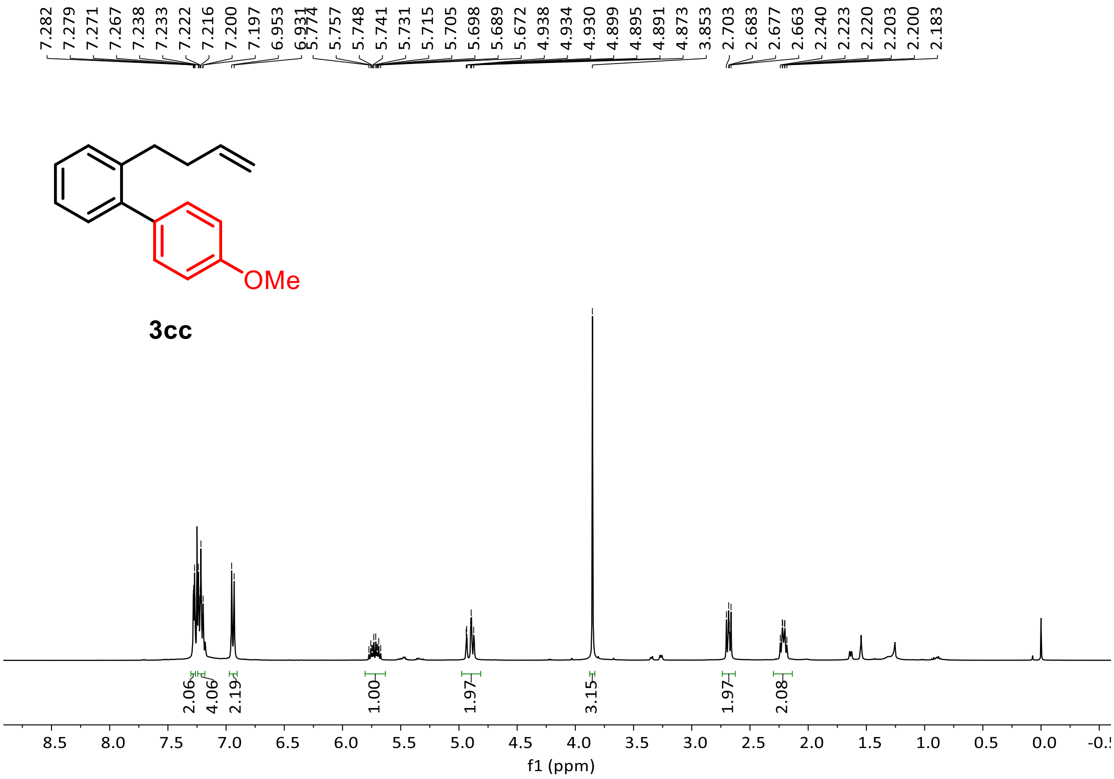

Supplementary Figure 17. ^{1}H NMR of compound **3cc** (400 MHZ, r.t., $CDCl_3$)

^{13}C NMR

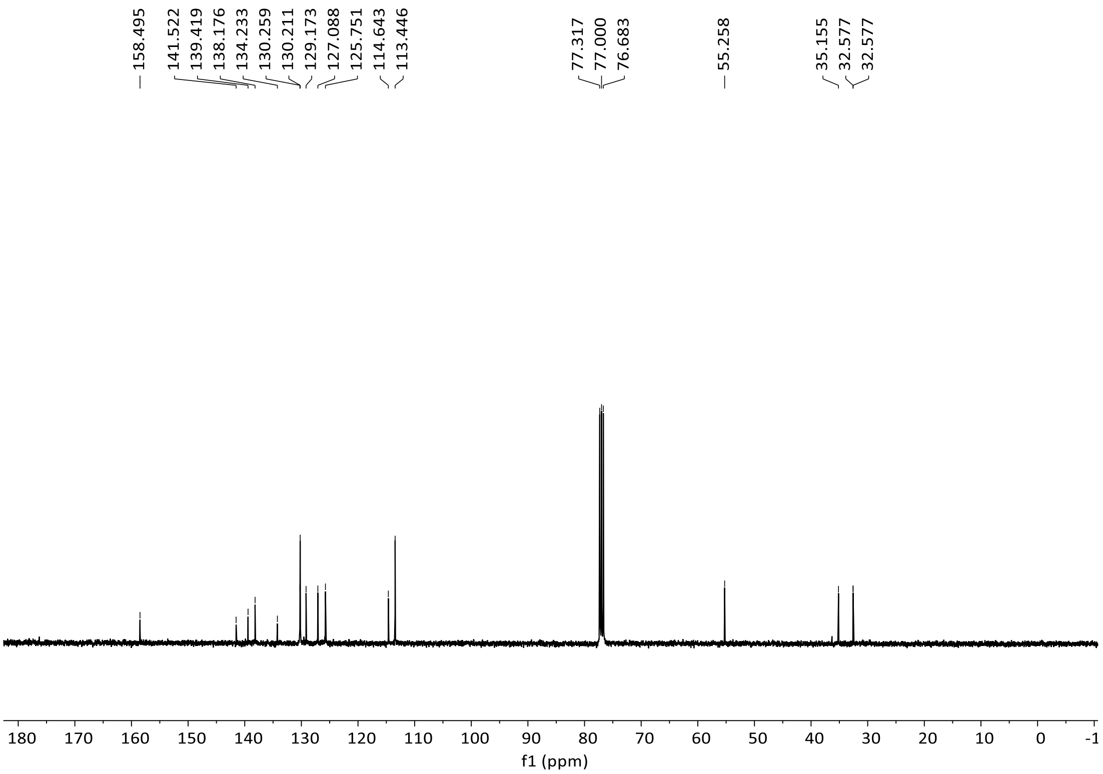

Supplementary Figure 18. ^{13}C NMR of compound **3cc** (101 MHZ, r.t., $CDCl_3$)

^{1}H NMR

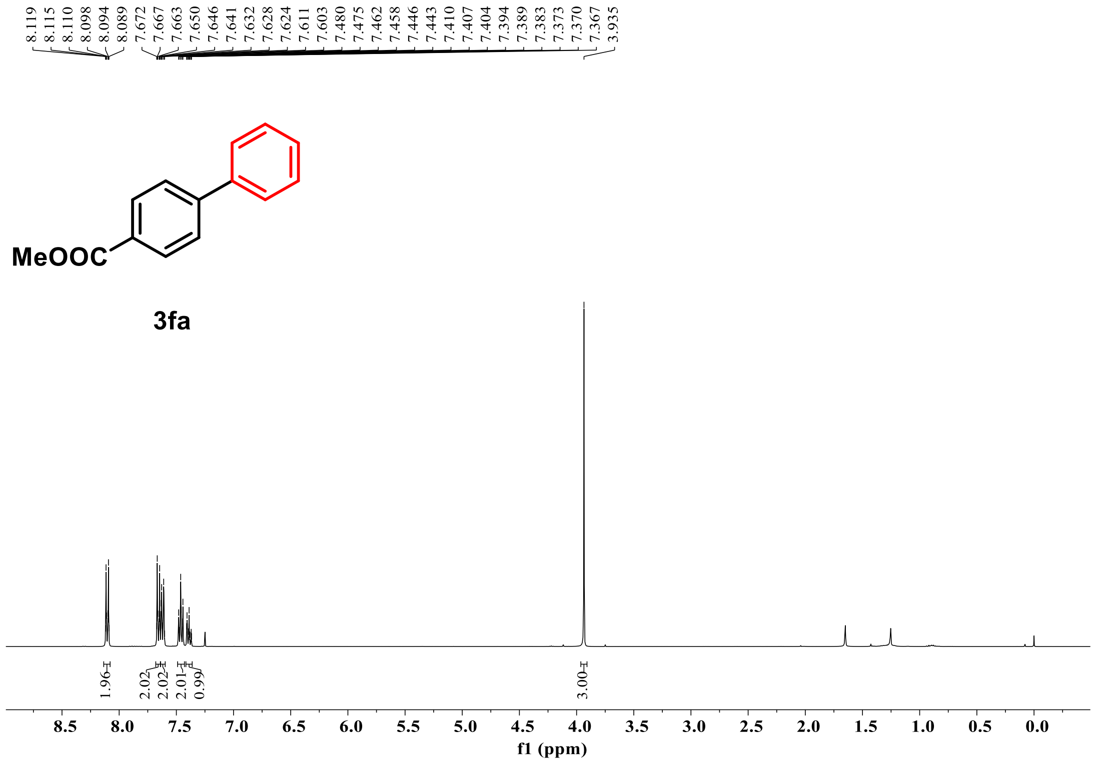

Supplementary Figure 19. ^{1}H NMR of compound **3fa** (400 MHZ, r.t., $CDCl_3$)

^{13}C NMR

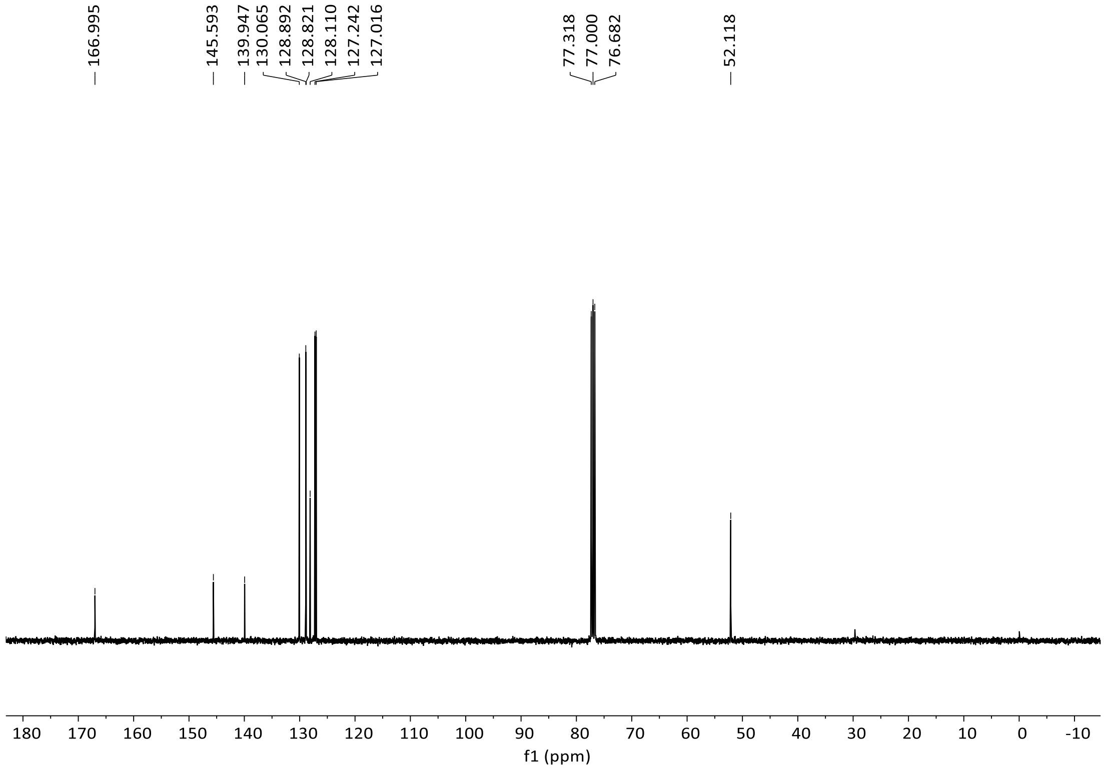

Supplementary Figure 20. ^{13}C NMR of compound **3fa** (101 MHZ, r.t., $CDCl_3$)

^{1}HNMR

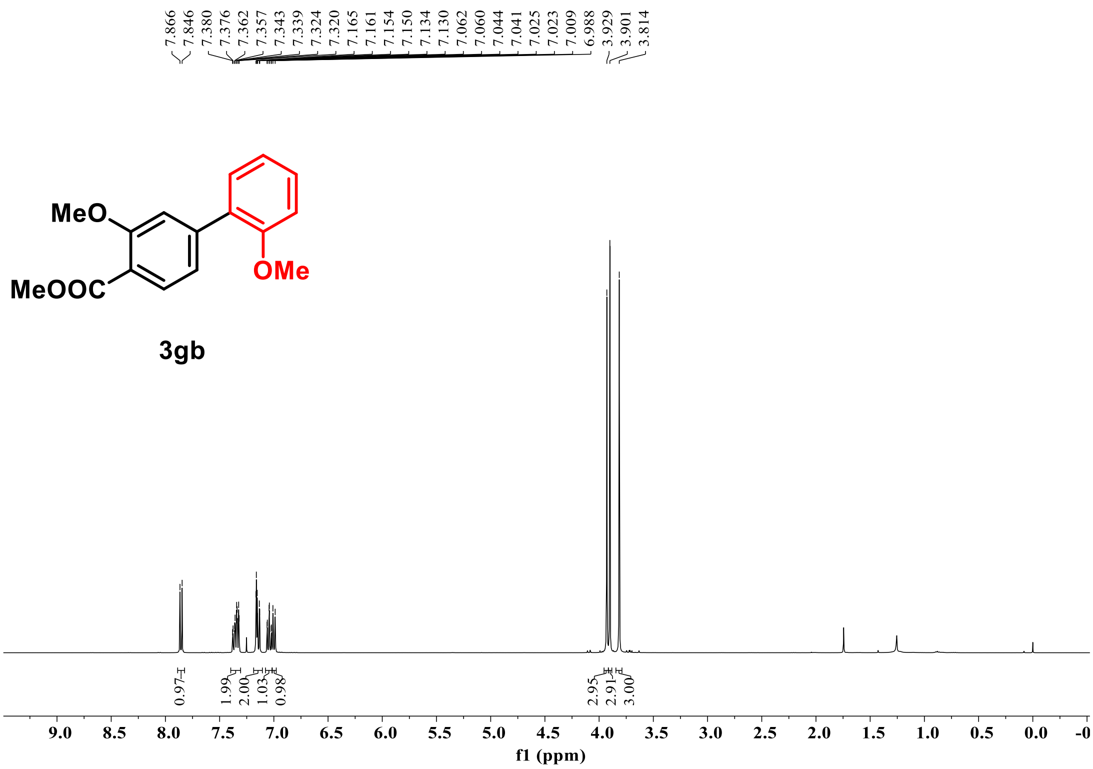

Supplementary Figure 21. ^{1}H NMR of compound **3gb** (400 MHZ, r.t., $CDCl_3$)

^{13}C NMR

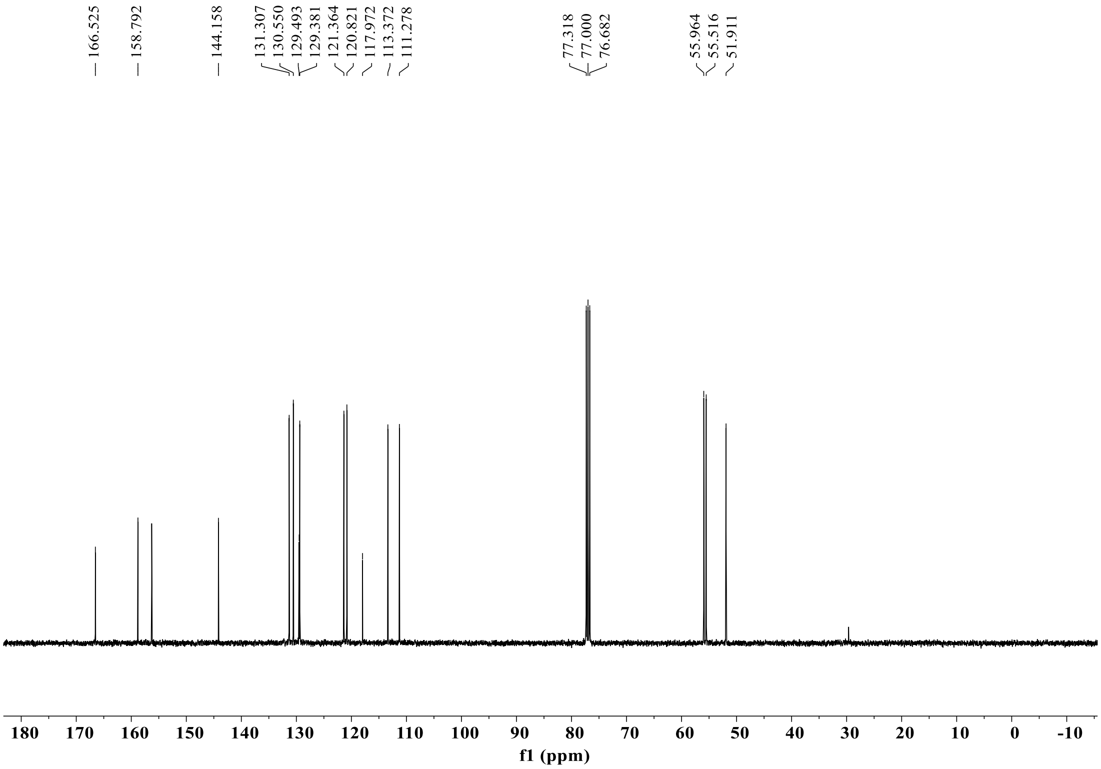

Supplementary Figure 22. ^{13}C NMR of compound **3gb** (101 MHZ, r.t., $CDCl_3$)

^{1}H NMR

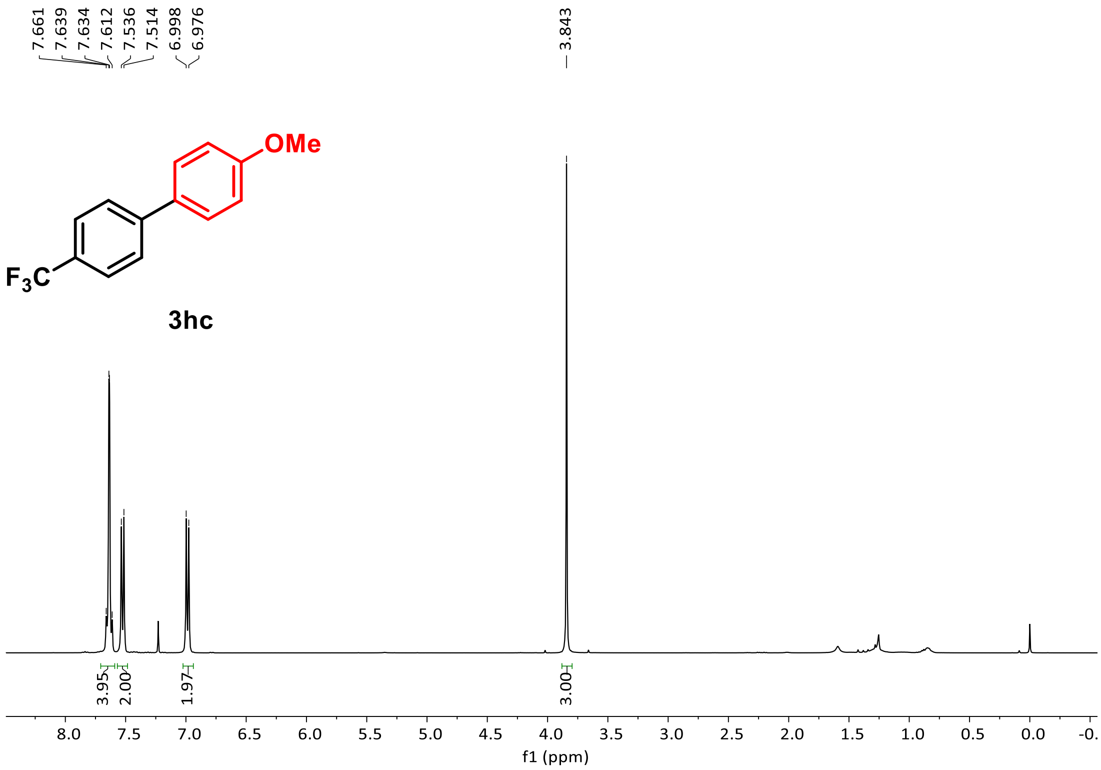

Supplementary Figure 23. ^{1}H NMR of compound **3hc** (400 MHZ, r.t., $CDCl_3$)

^{13}C NMR

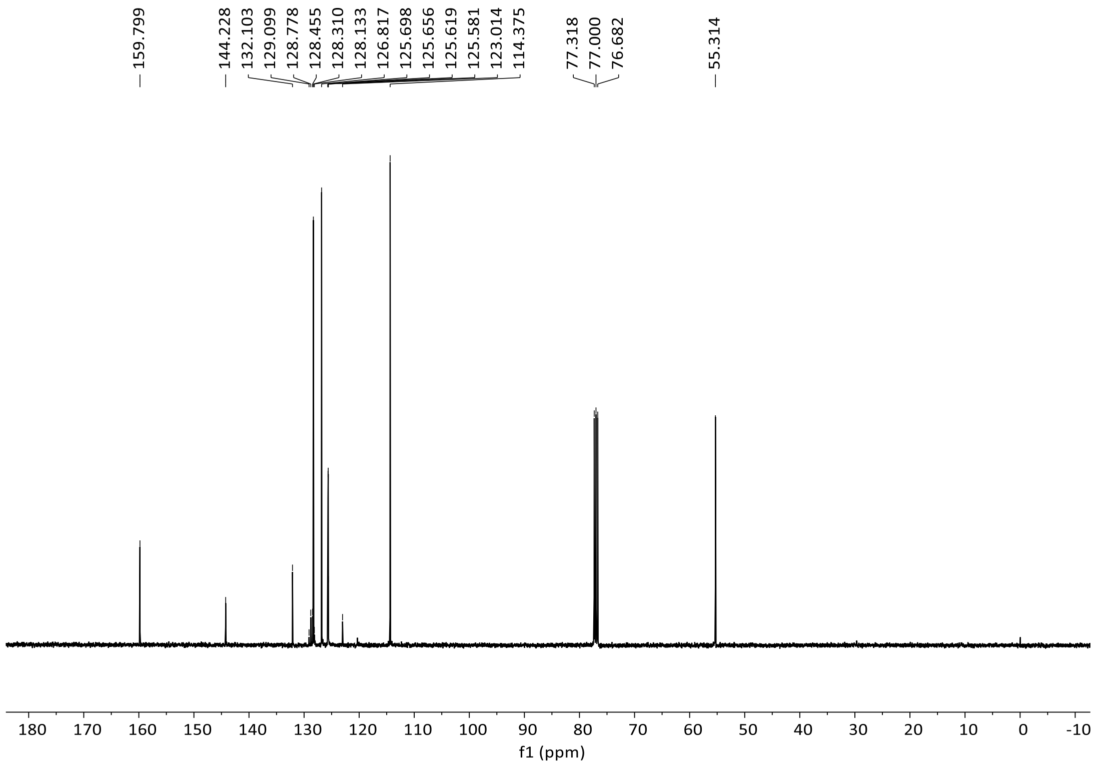

Supplementary Figure 24. ^{13}C NMR of compound **3hc** (101 MHZ, r.t., $CDCl_3$)

^{19}F NMR

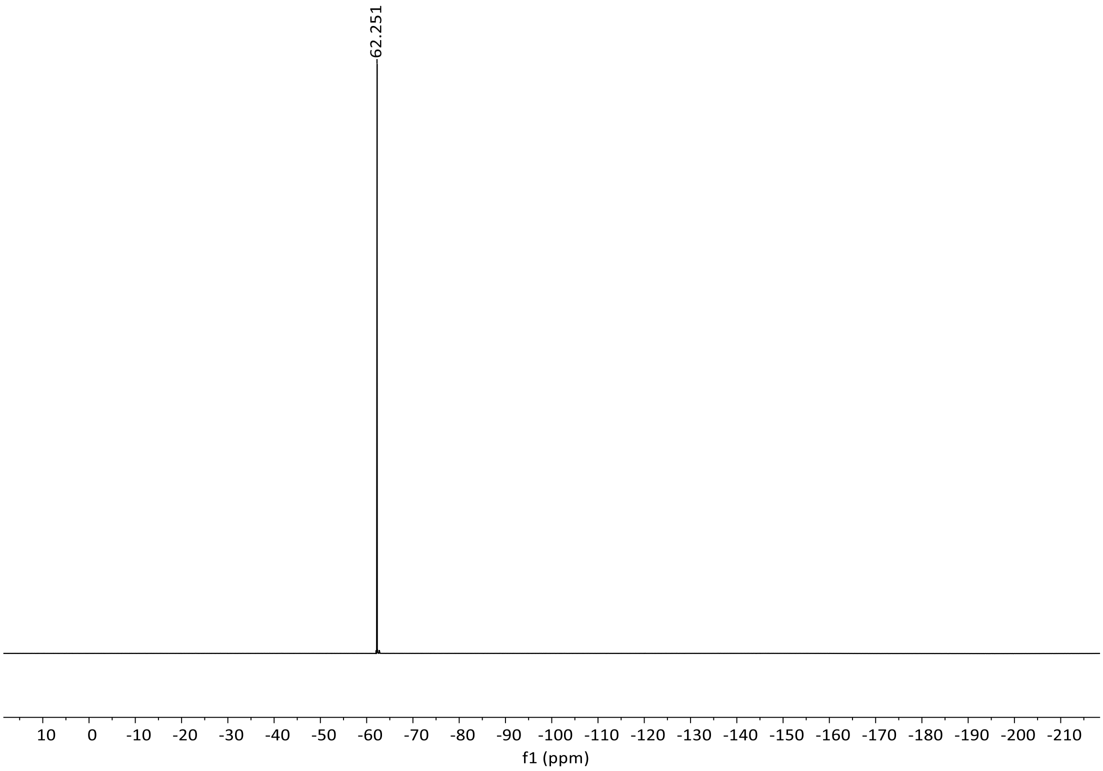

Supplementary Figure 25. ^{1}F NMR of compound **3hc** (377 MHz, r.t., $CDCl_3$)

^{1}H NMR

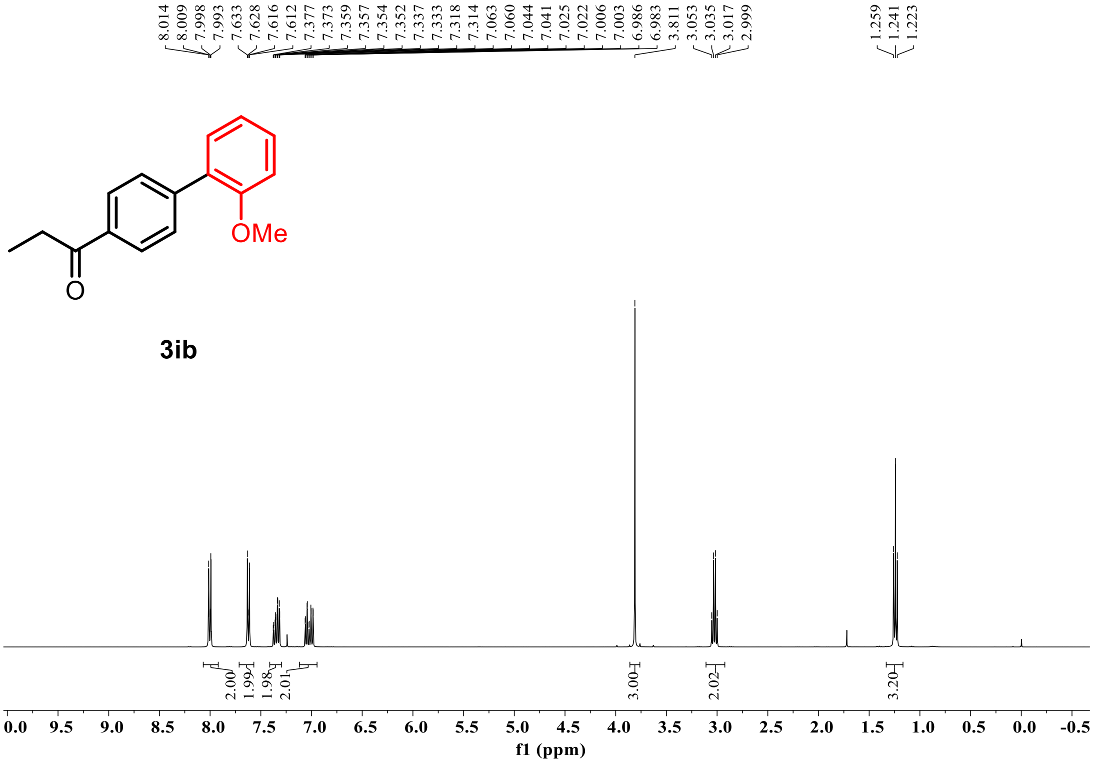

Supplementary Figure 26. ^{1}H NMR of compound **3ib** (400 MHZ, r.t., $CDCl_3$)

^{13}C NMR

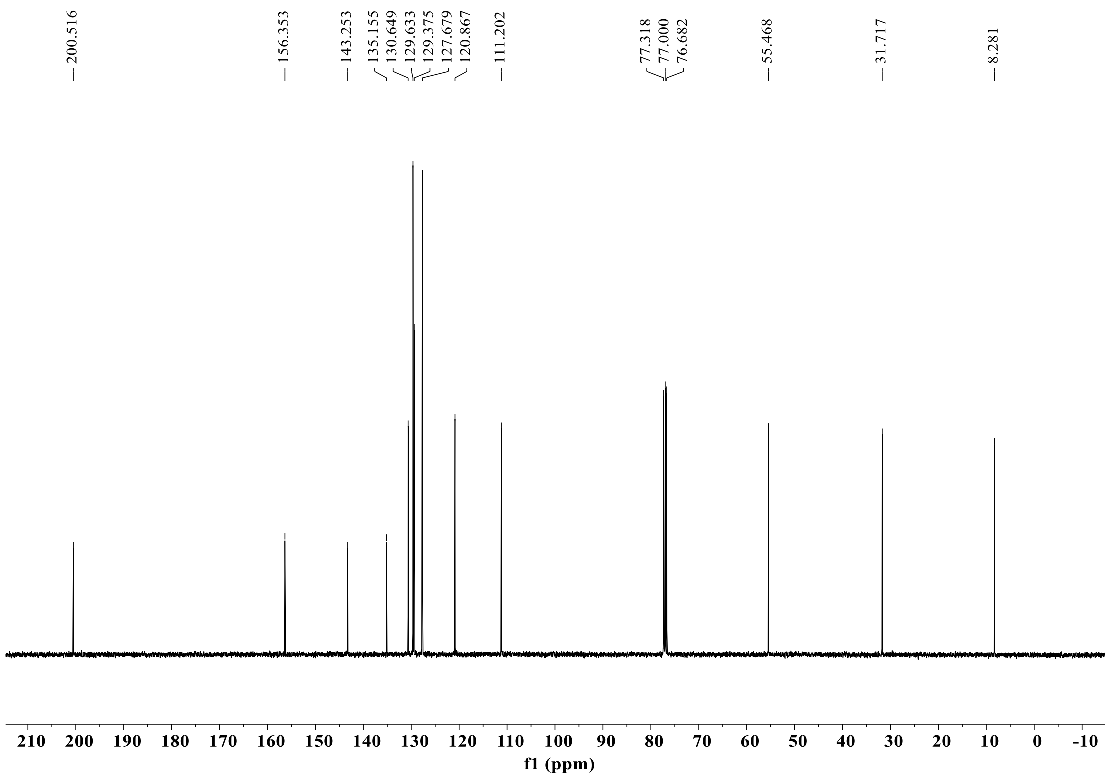

Supplementary Figure 27. ^{13}C NMR of compound **3ib** (101 MHZ, r.t., $CDCl_3$)

^{1}H NMR

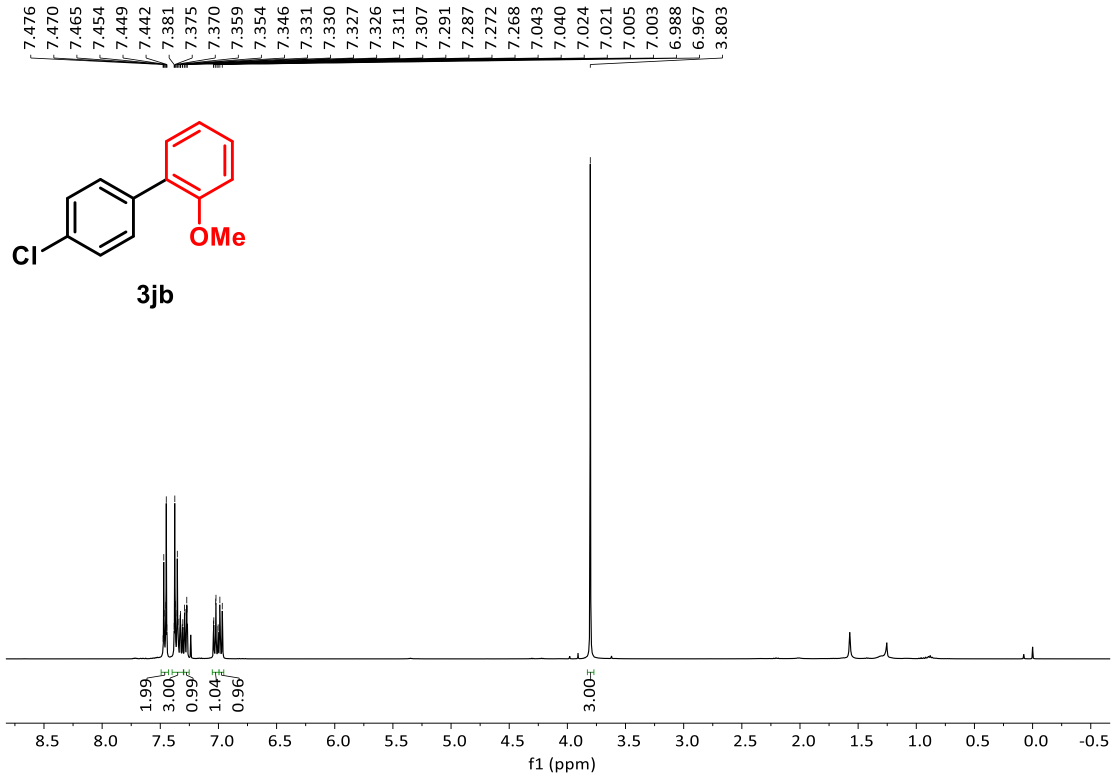

Supplementary Figure 28. ^{1}H NMR of compound **3jb** (400 MHZ, r.t., $CDCl_3$)

^{13}C NMR

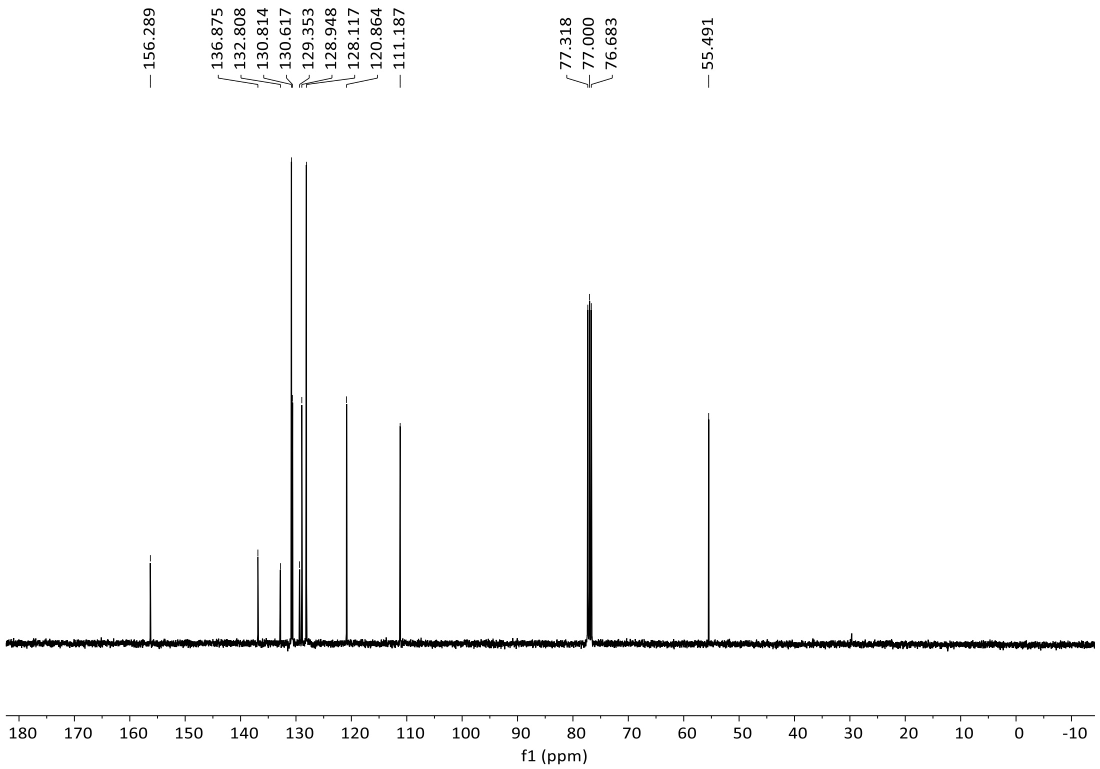

Supplementary Figure 29. ^{13}C NMR of compound **3jb** (101 MHZ, r.t., $CDCl_3$)

^{1}H NMR

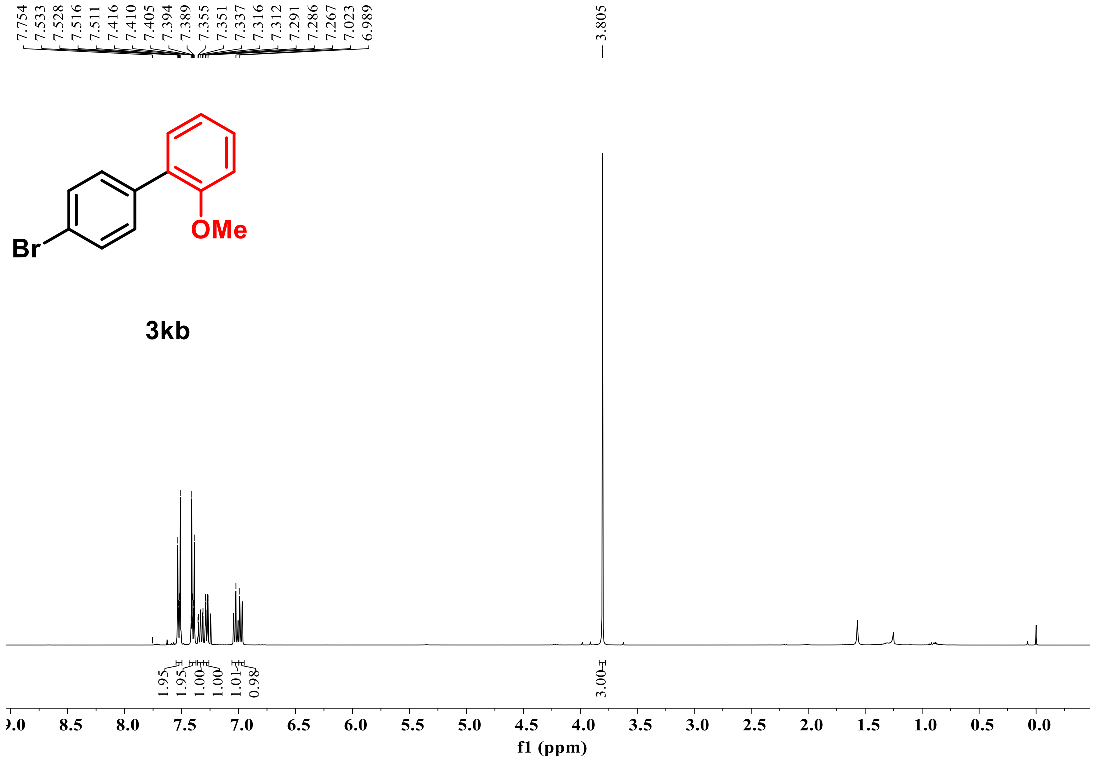

Supplementary Figure 30. ^{1}H NMR of compound **3kb** (400 MHZ, r.t., $CDCl_3$)

^{13}C NMR

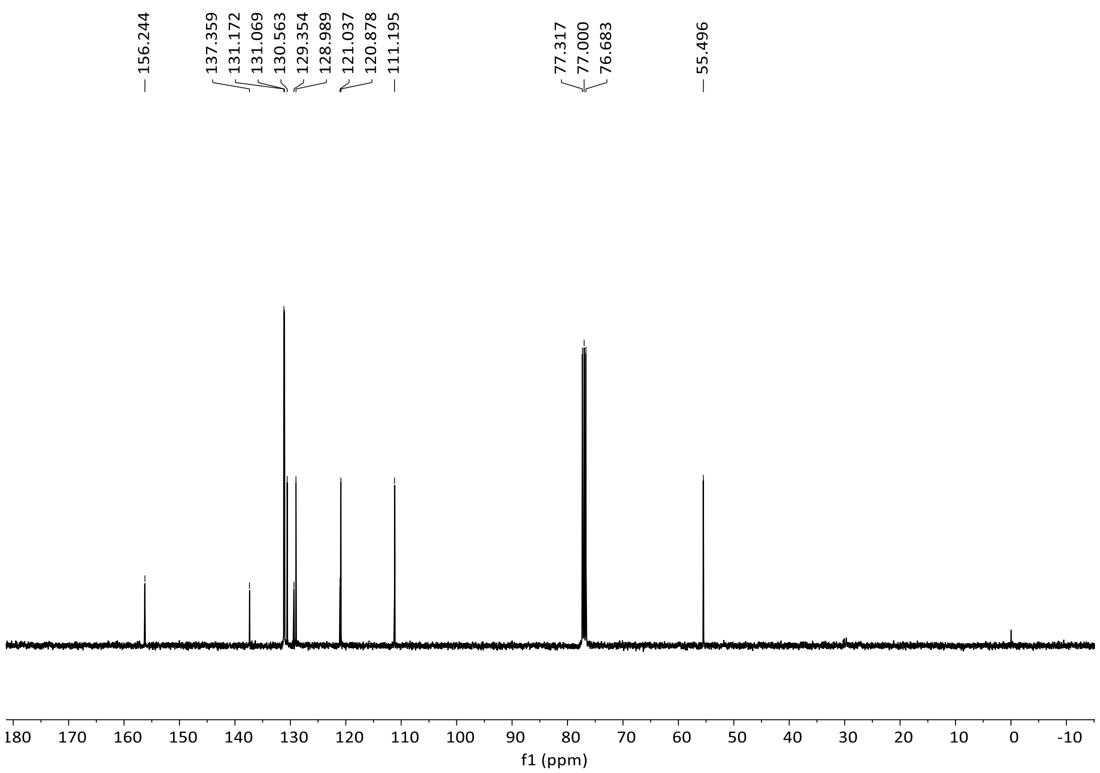

Supplementary Figure 31. ^{13}C NMR of compound **3kb** (101 MHZ, r.t., $CDCl_3$)

^{1}H NMR

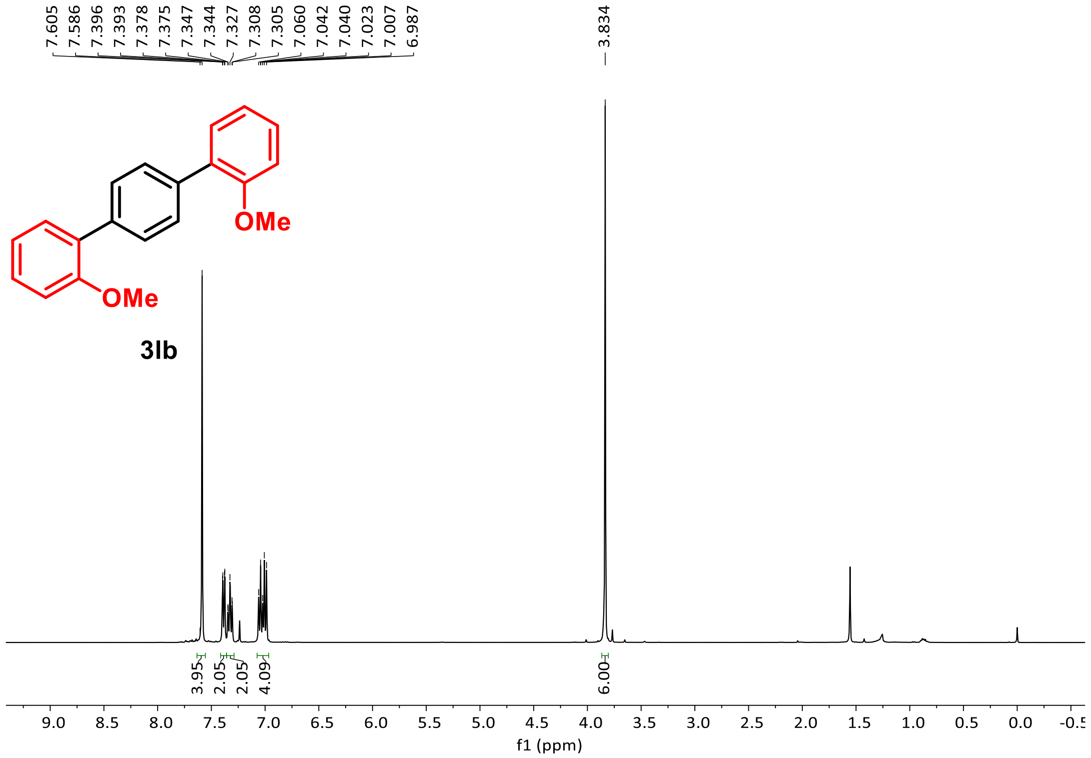

Supplementary Figure 32. ^{1}H NMR of compound **3lb** (400 MHZ, r.t., $CDCl_3$)

^{13}C NMR

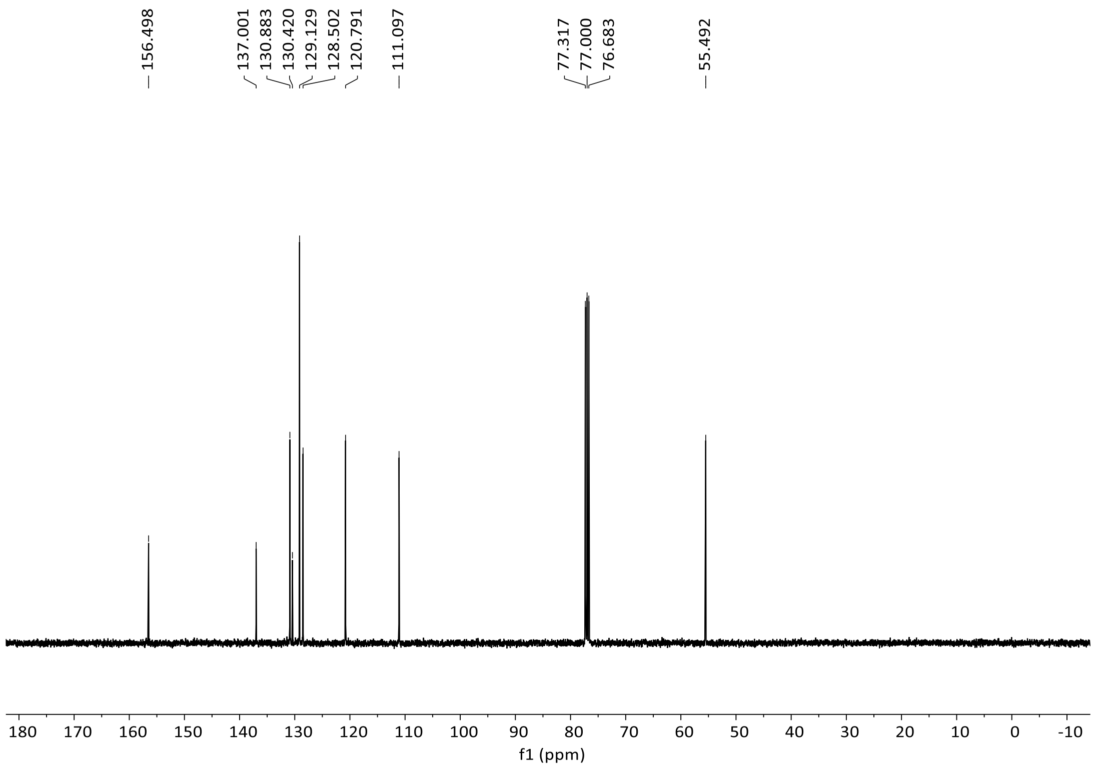

Supplementary Figure 33. ^{13}C NMR of compound **3lb** (101 MHZ, r.t., $CDCl_3$)

^{1}H NMR

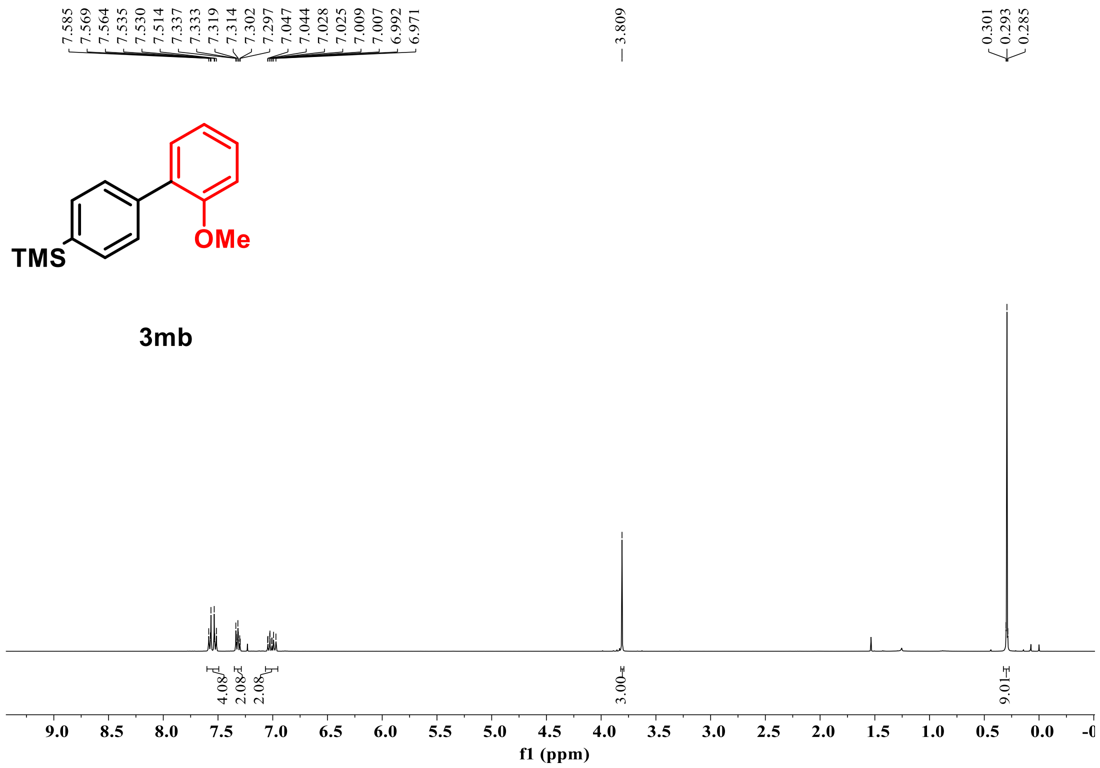

Supplementary Figure 34. ^{1}H NMR of compound **3mb** (400 MHZ, r.t., $CDCl_3$)

^{13}C NMR

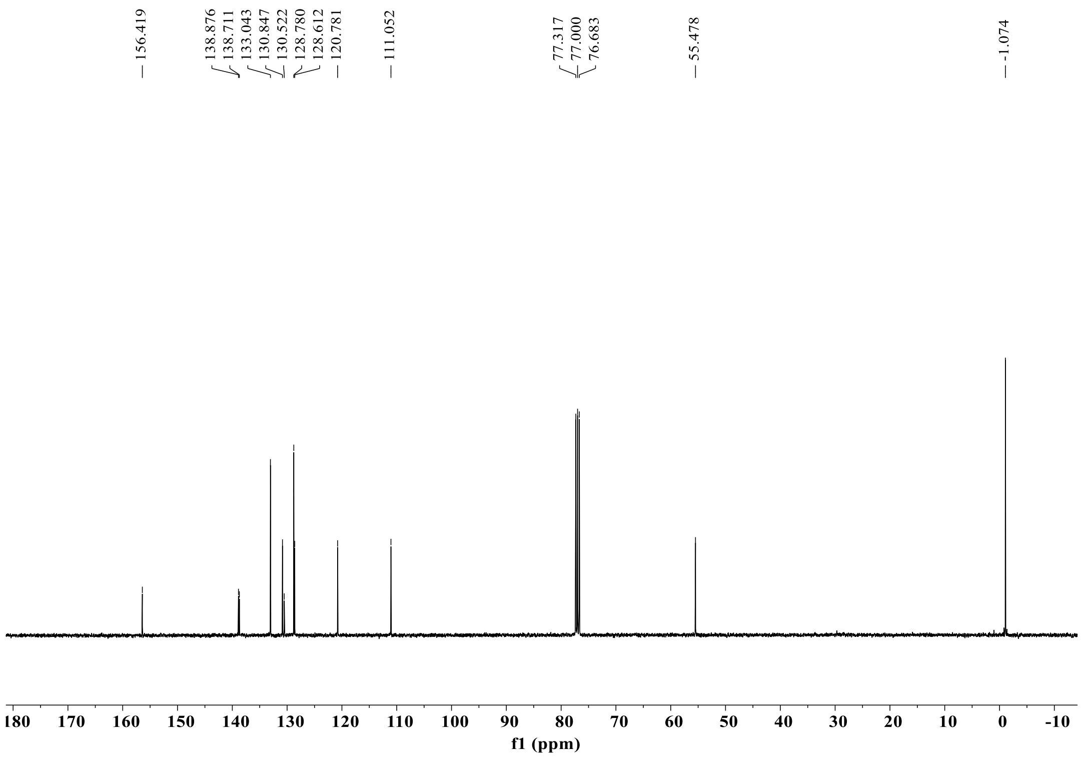

Supplementary Figure 25. ^{13}C NMR of compound **3mb** (101 MHZ, r.t., $CDCl_3$)

^{1}H NMR

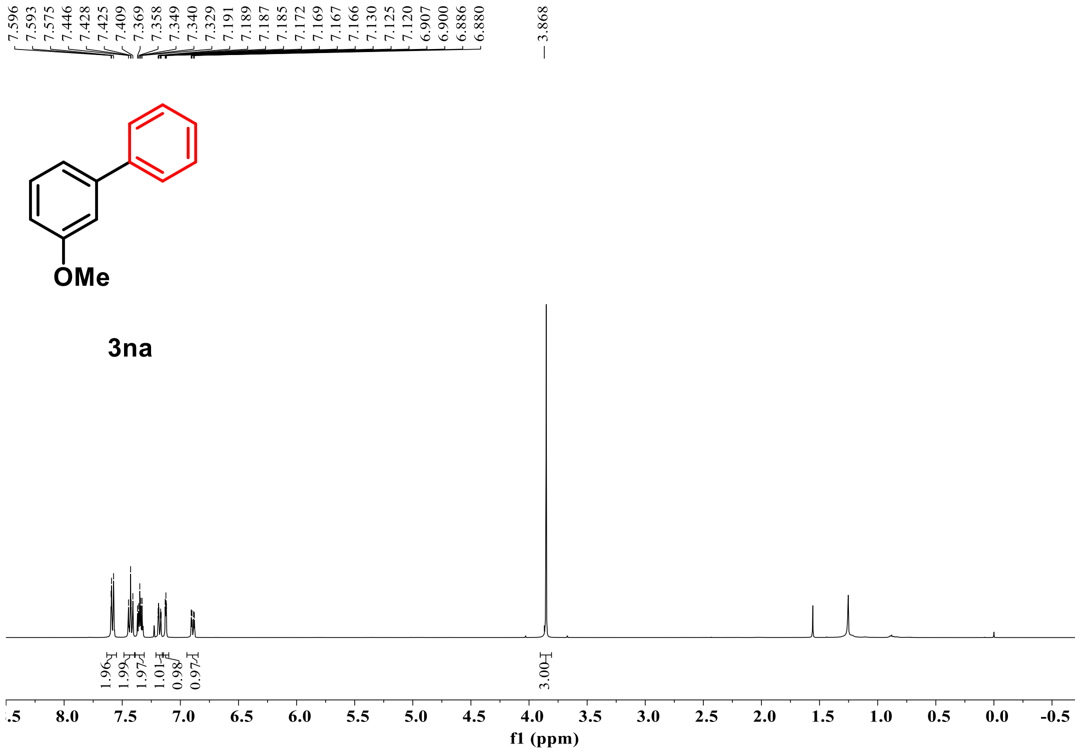

Supplementary Figure 36. ^{1}H NMR of compound **3na** (400 MHZ, r.t., $CDCl_3$)

^{13}C NMR

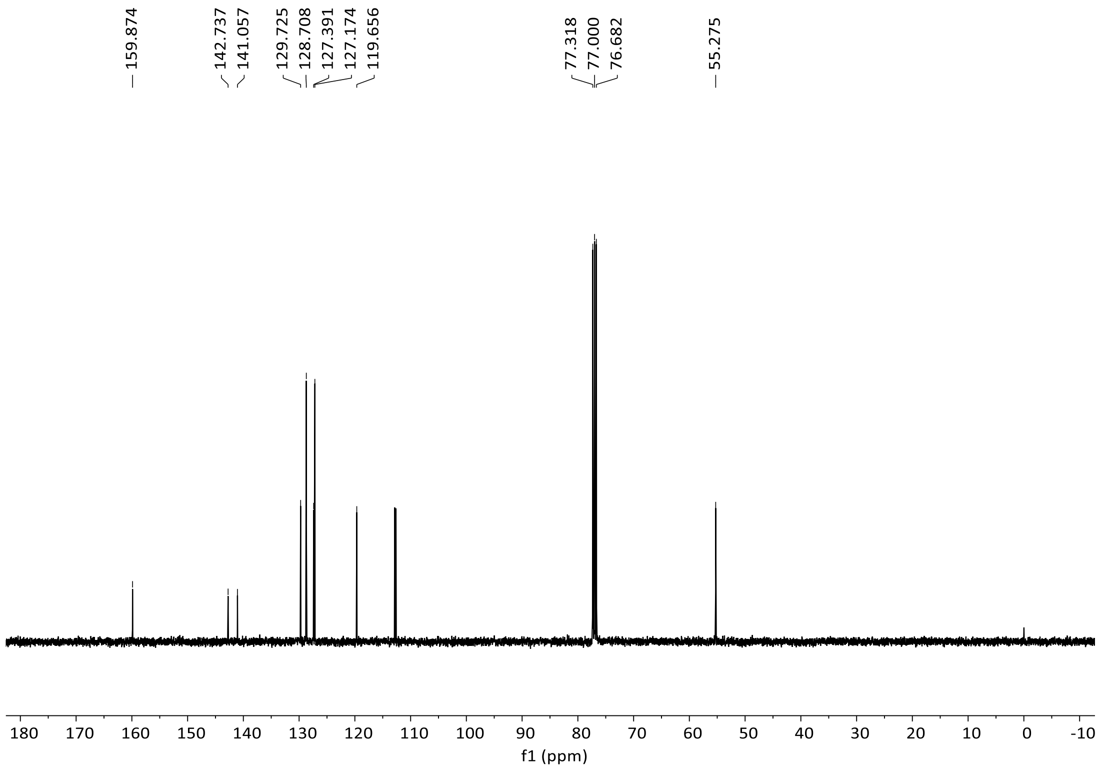

Supplementary Figure 37. ^{13}C NMR of compound **3na** (101 MHZ, r.t., $CDCl_3$)

^{1}H NMR

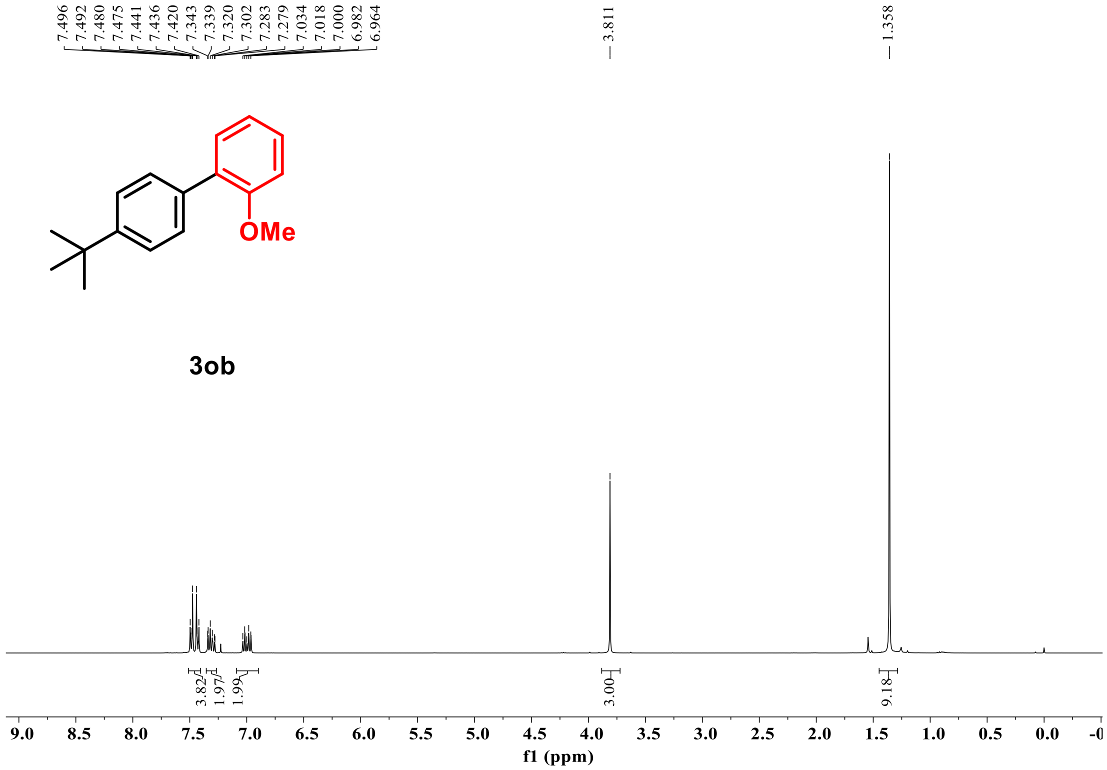

Supplementary Figure 38. ^{1}H NMR of compound **3ob** (400 MHZ, r.t., $CDCl_3$)

^{13}C NMR

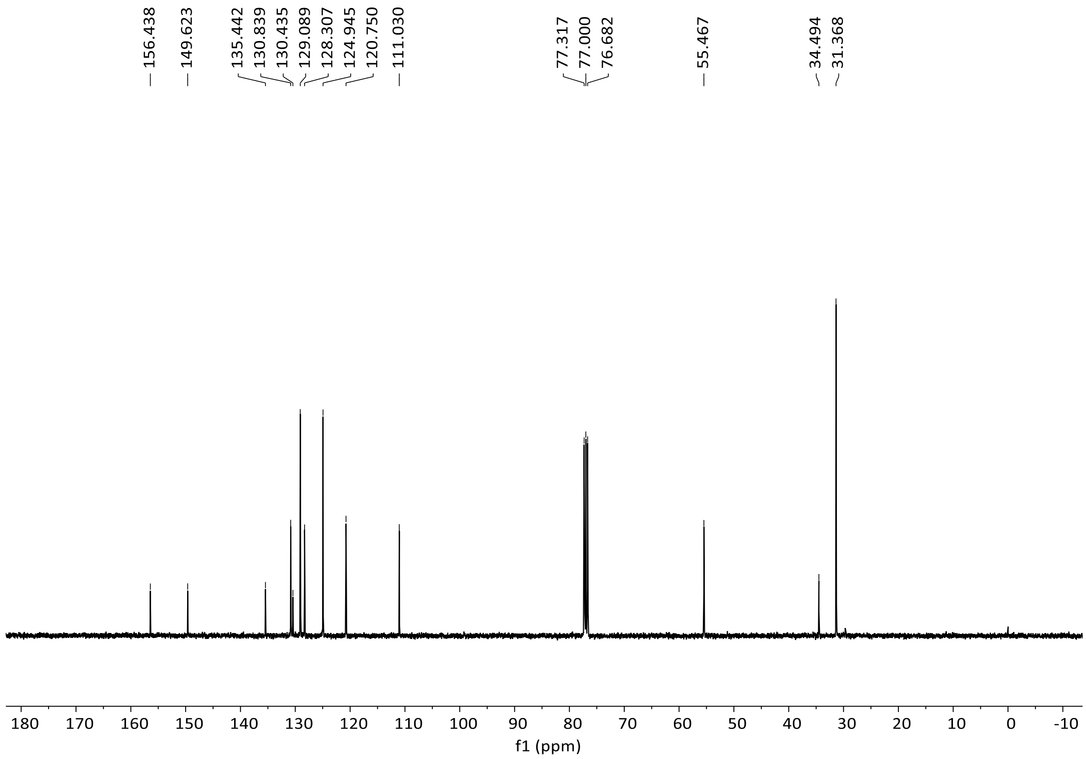

Supplementary Figure 39. ^{13}C NMR of compound **3ob** (101 MHZ, r.t., $CDCl_3$)

^{1}H NMR

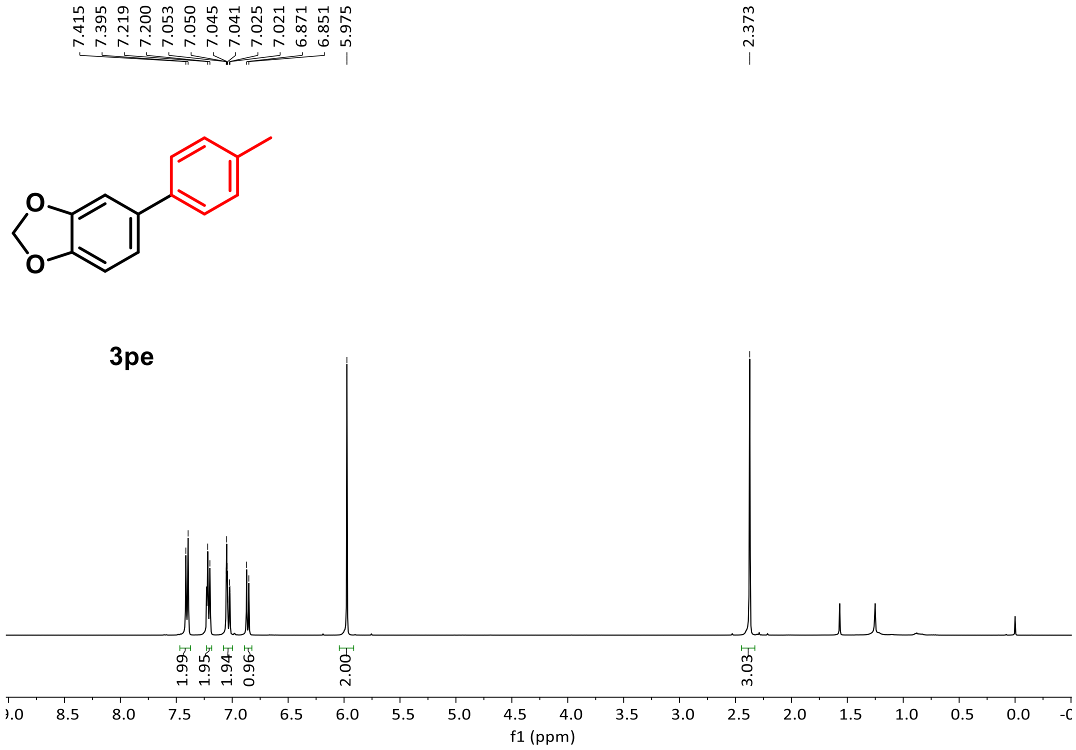

Supplementary Figure 40. ^{1}H NMR of compound **3pe** (400 MHZ, r.t., $CDCl_3$)

^{13}C NMR

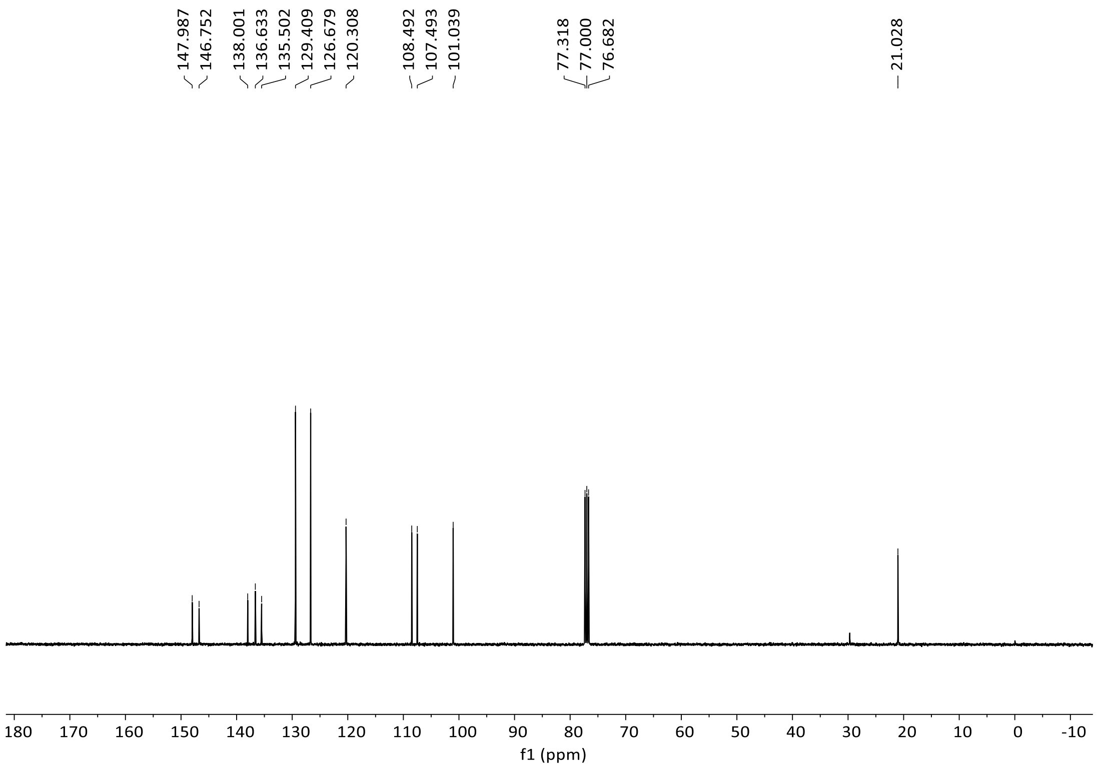

Supplementary Figure 41. ^{13}C NMR of compound **3pe** (101 MHZ, r.t., $CDCl_3$)

^{1}H NMR

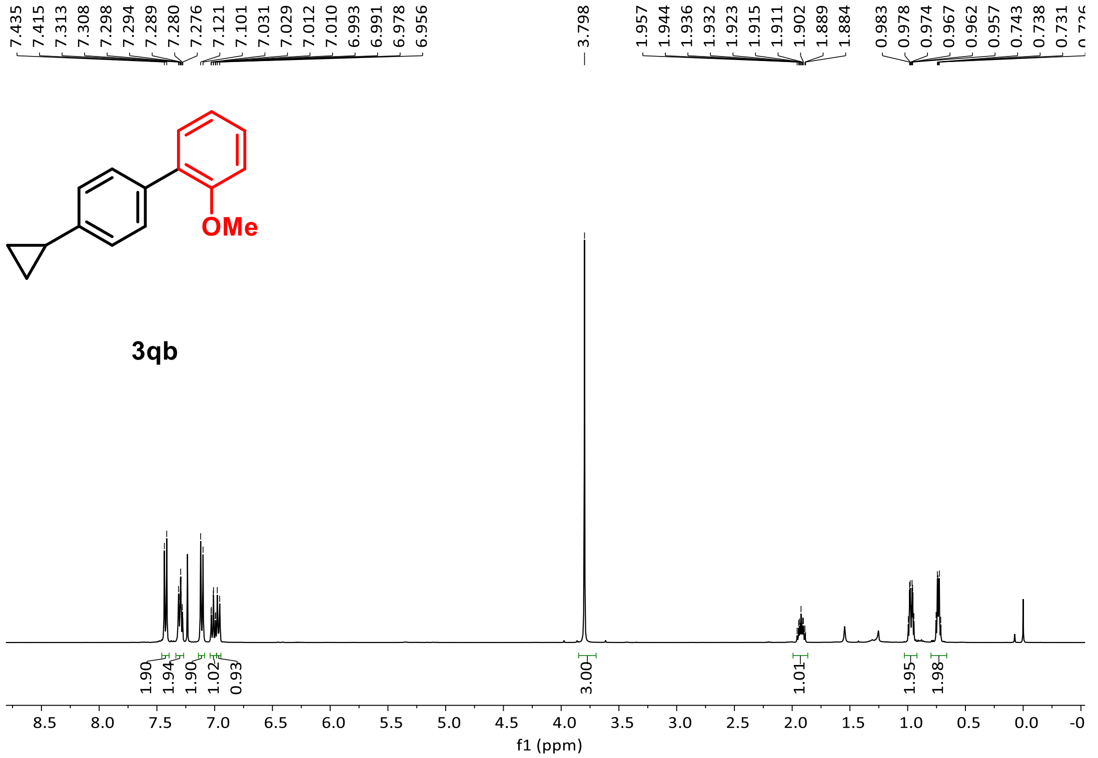

Supplementary Figure 42. ^{1}H NMR of compound **3qb** (400 MHZ, r.t., $CDCl_3$)

^{13}C NMR

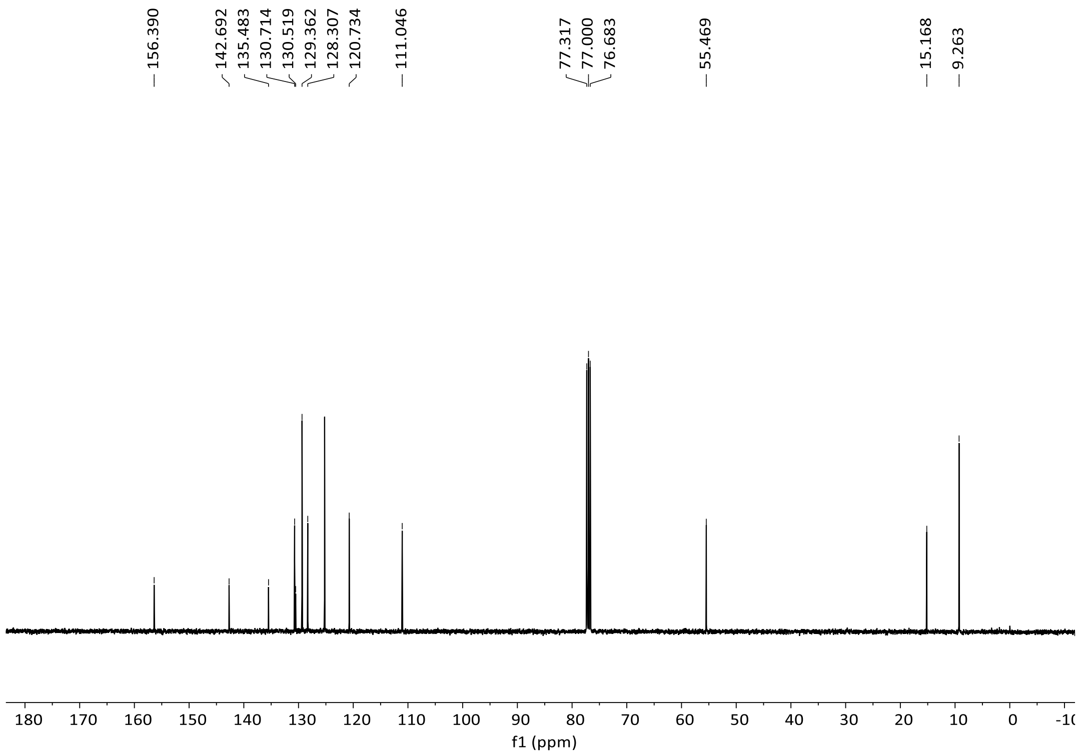

Supplementary Figure 43. ^{13}C NMR of compound **3qb** (101 MHZ, r.t., $CDCl_3$)

^{1}H NMR

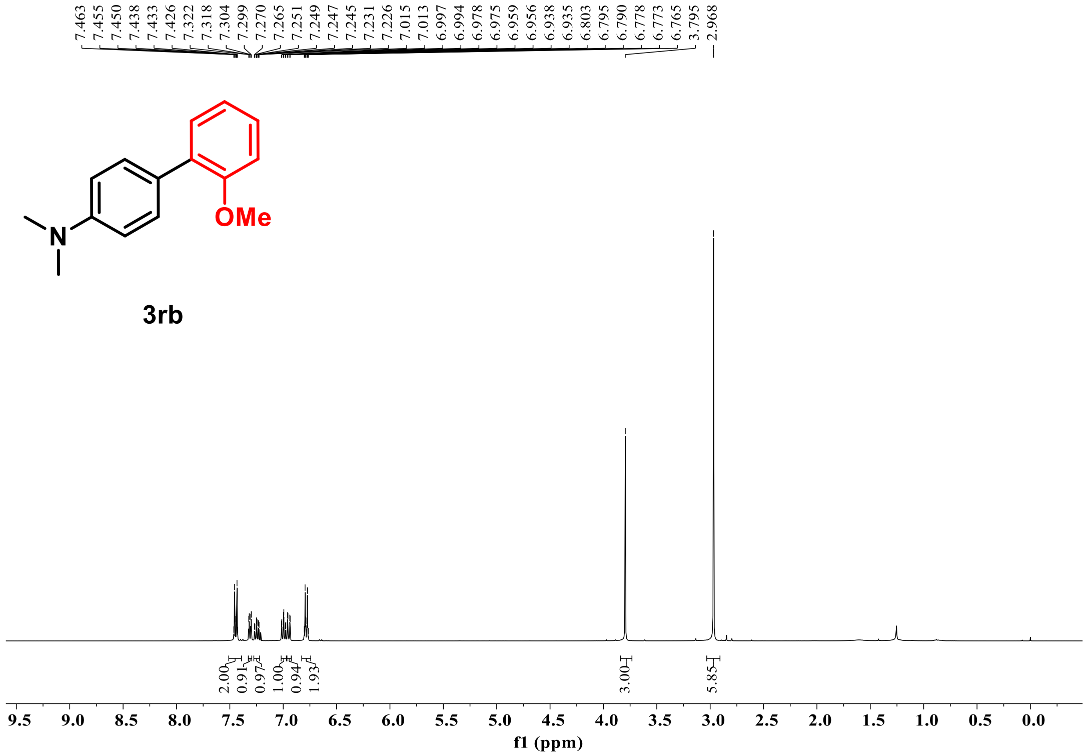

Supplementary Figure 44. ^{1}H NMR of compound **3rb** (400 MHZ, r.t., $CDCl_3$)

^{13}C NMR

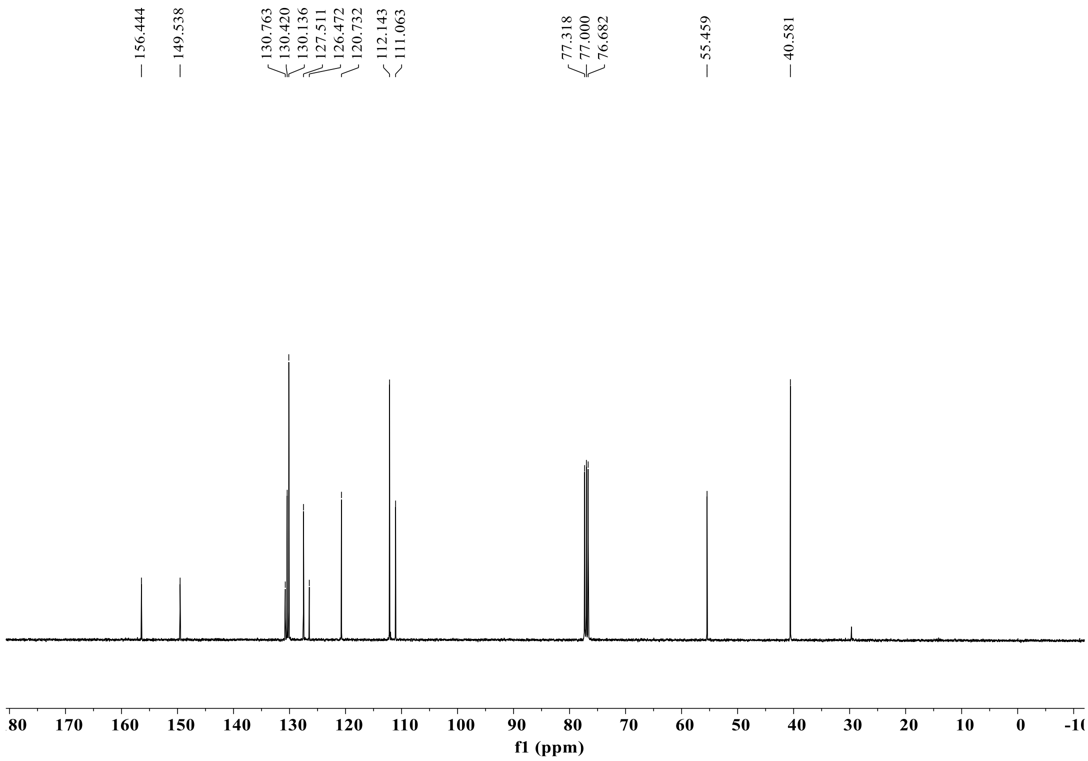

Supplementary Figure 45. ^{13}C NMR of compound **3rb** (101 MHZ, r.t., $CDCl_3$)

^{1}H NMR

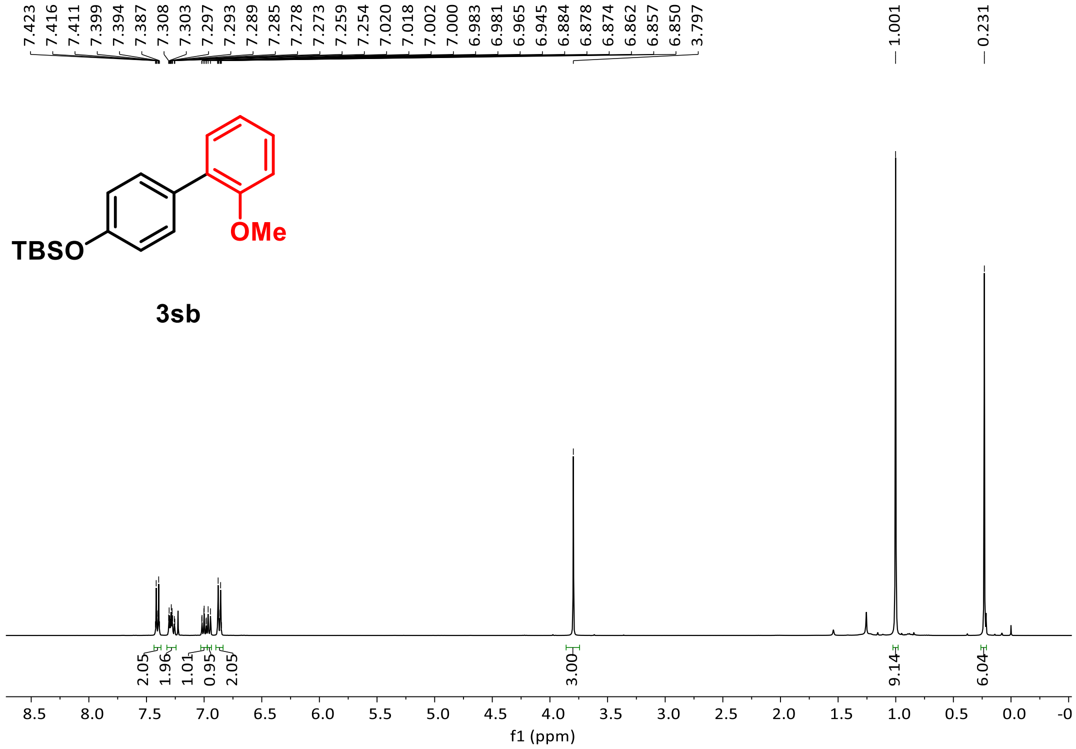

Supplementary Figure 46. ^{1}H NMR of compound **3sb** (400 MHZ, r.t., $CDCl_3$)

^{13}C NMR

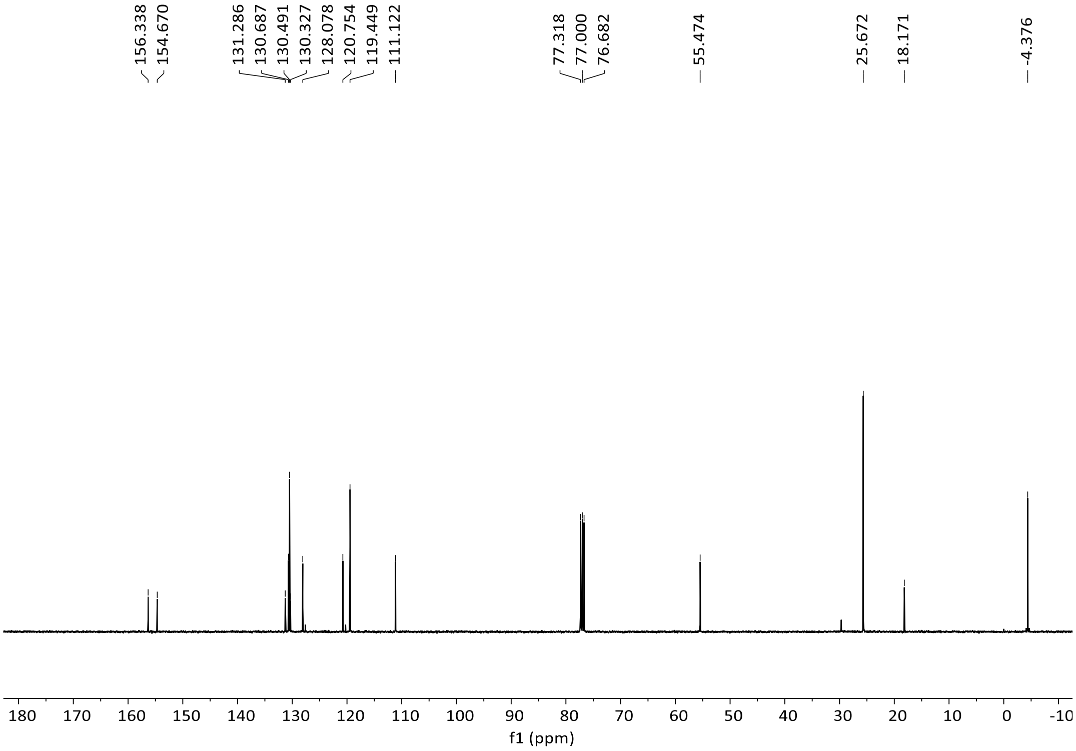

Supplementary Figure 47. ^{13}C NMR of compound **3sb** (101 MHZ, r.t., $CDCl_3$)

^{1}H NMR

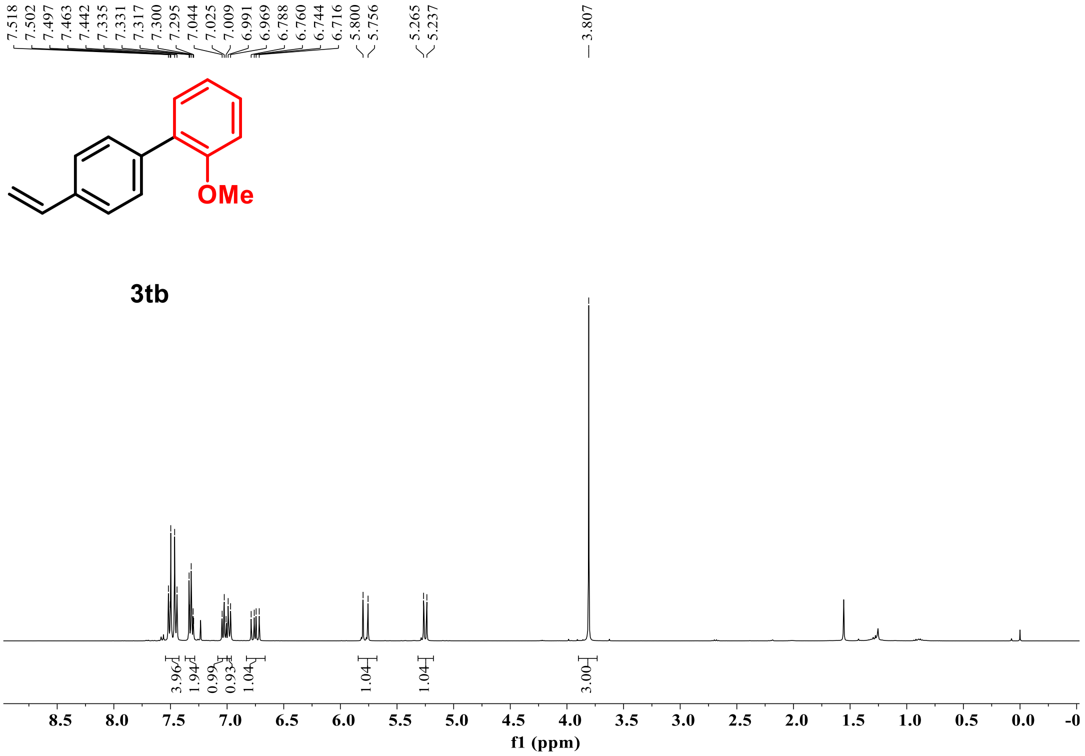

Supplementary Figure 48. ^{1}H NMR of compound **3tb** (400 MHZ, r.t., $CDCl_3$)

^{13}C NMR

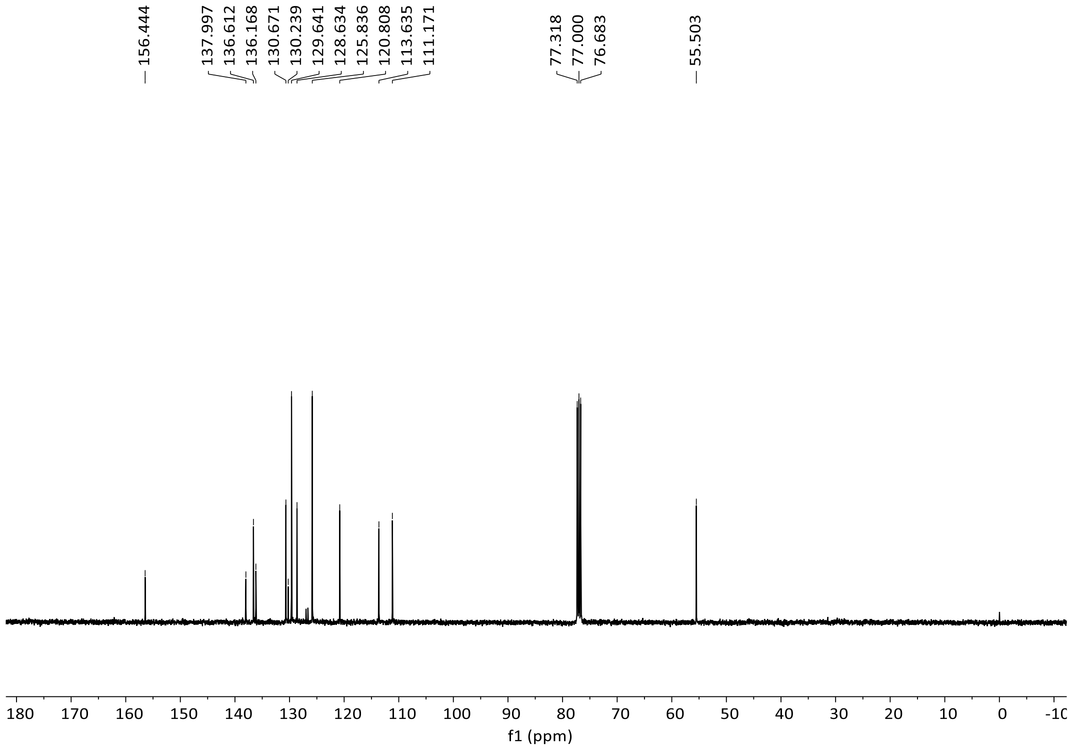

Supplementary Figure 49. ^{13}C NMR of compound **3tb** (101 MHZ, r.t., $CDCl_3$)

^{1}H NMR

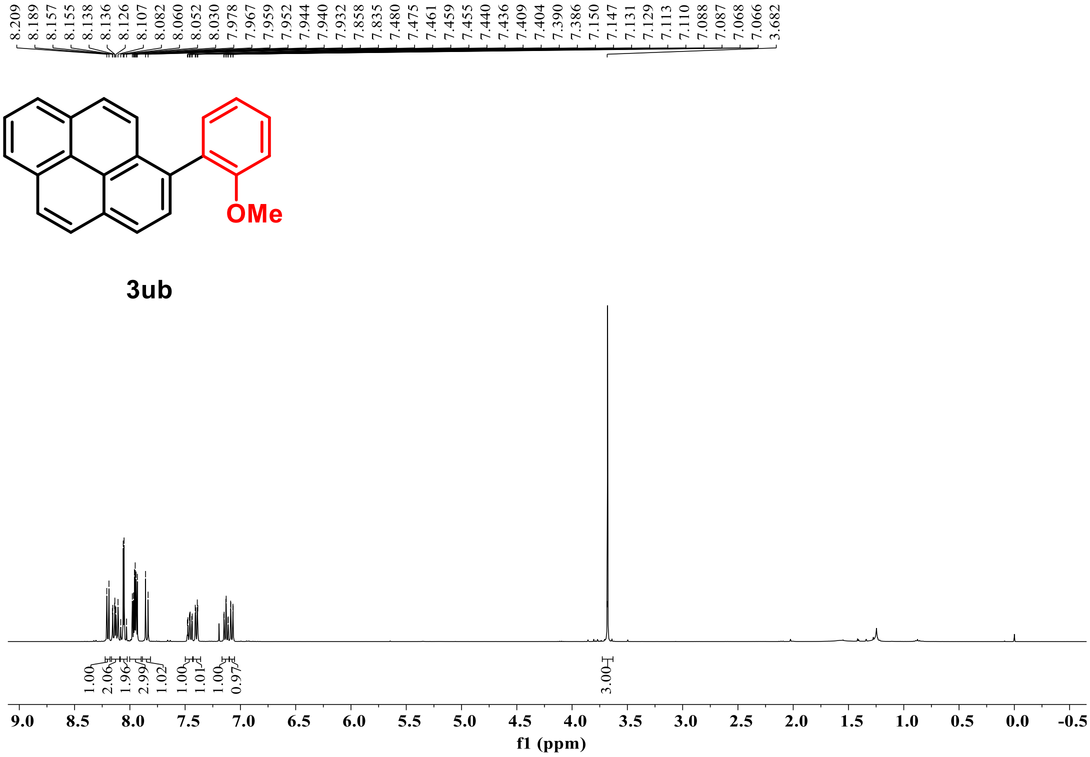

Supplementary Figure 50. ^{1}H NMR of compound **3ub** (400 MHZ, r.t., $CDCl_3$)

^{13}C NMR

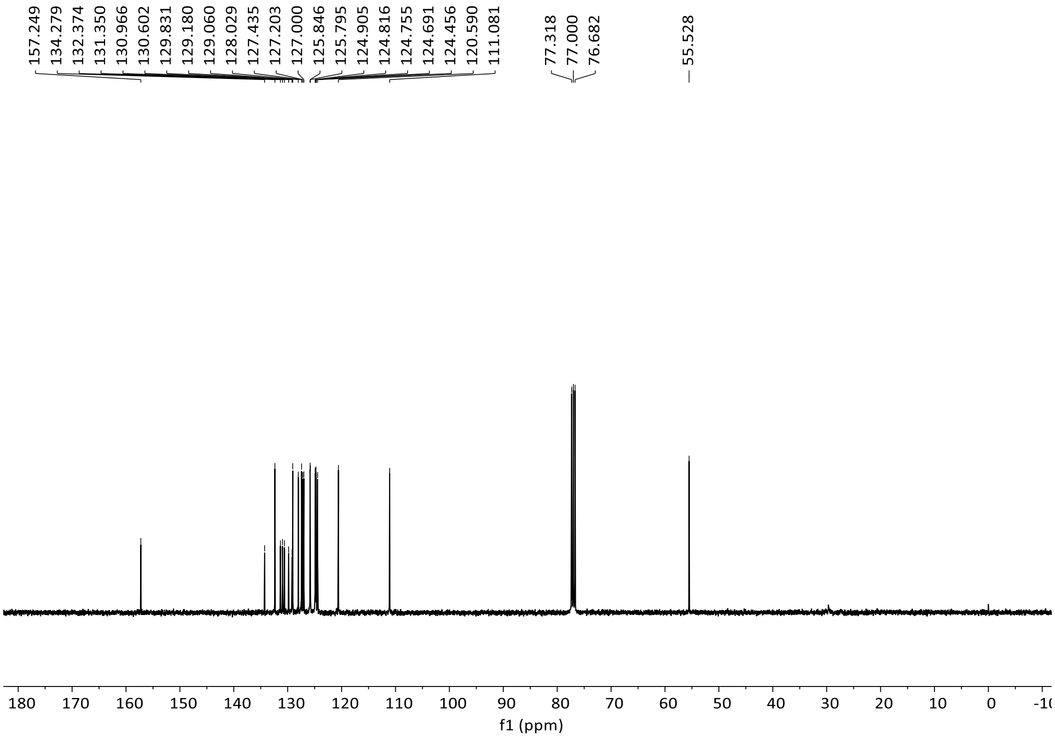

Supplementary Figure 51. ^{13}C NMR of compound **3ub** (101 MHZ, r.t., $CDCl_3$)

^{1}H NMR

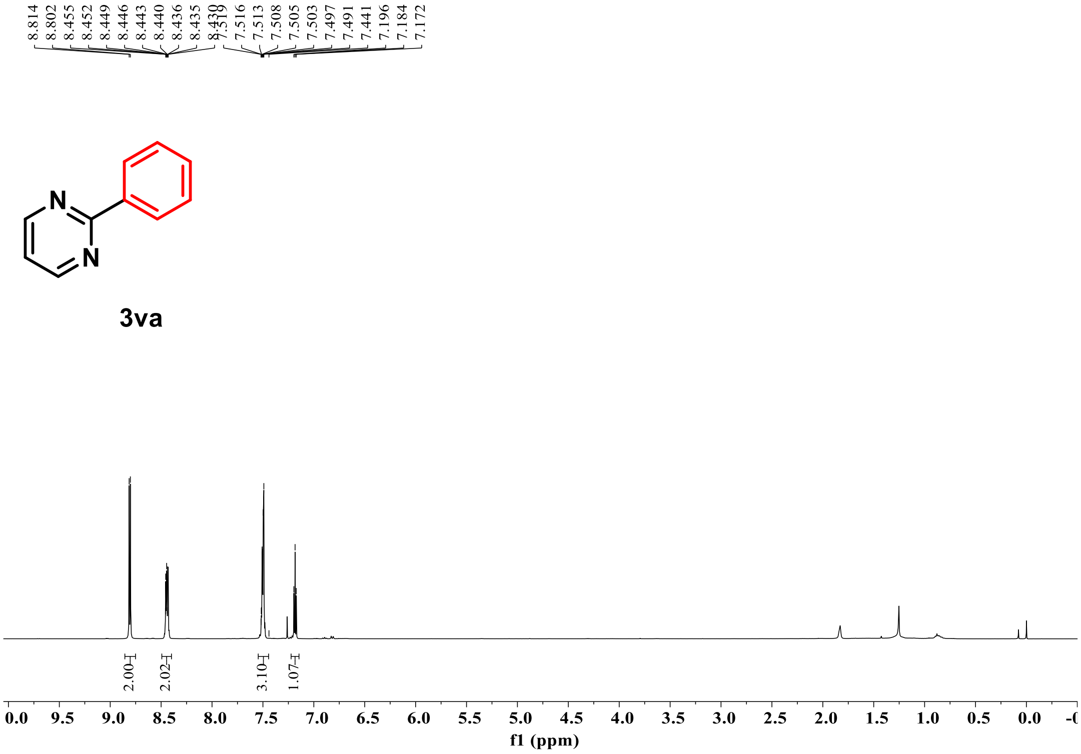

Supplementary Figure 52. ^{1}H NMR of compound **3va** (400 MHZ, r.t., $CDCl_3$)

^{13}C NMR

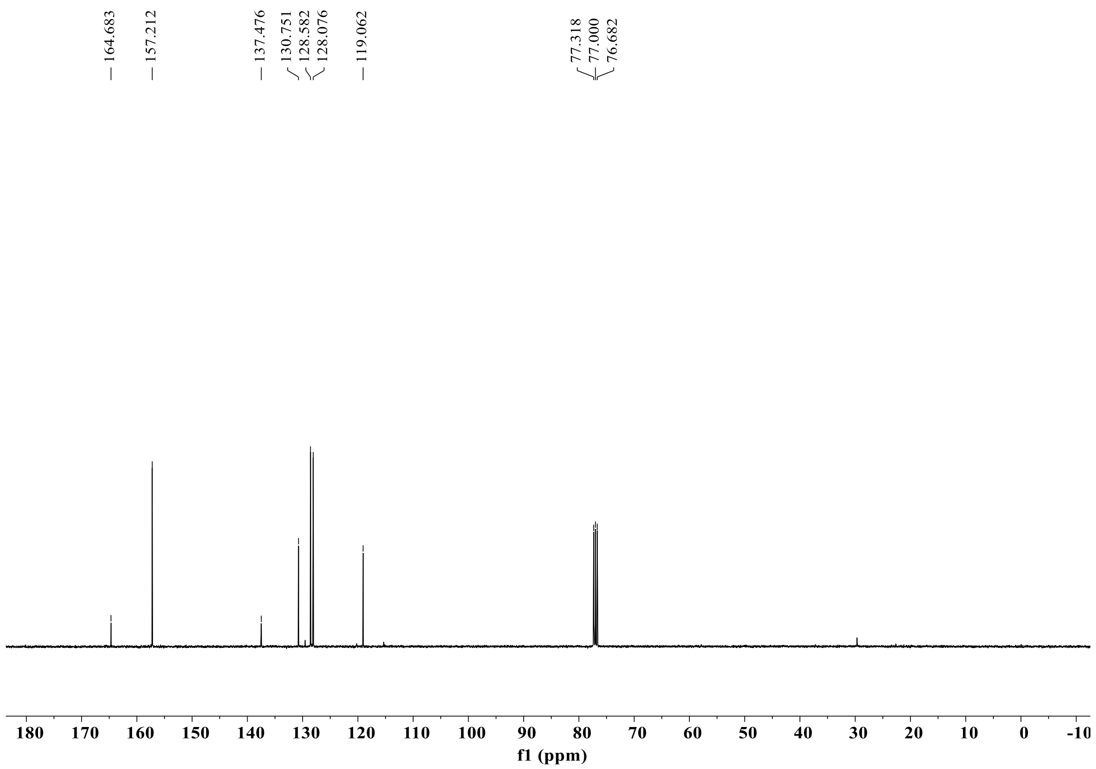

Supplementary Figure 53. ^{13}C NMR of compound **3va** (101 MHZ, r.t., $CDCl_3$)

^{1}H NMR

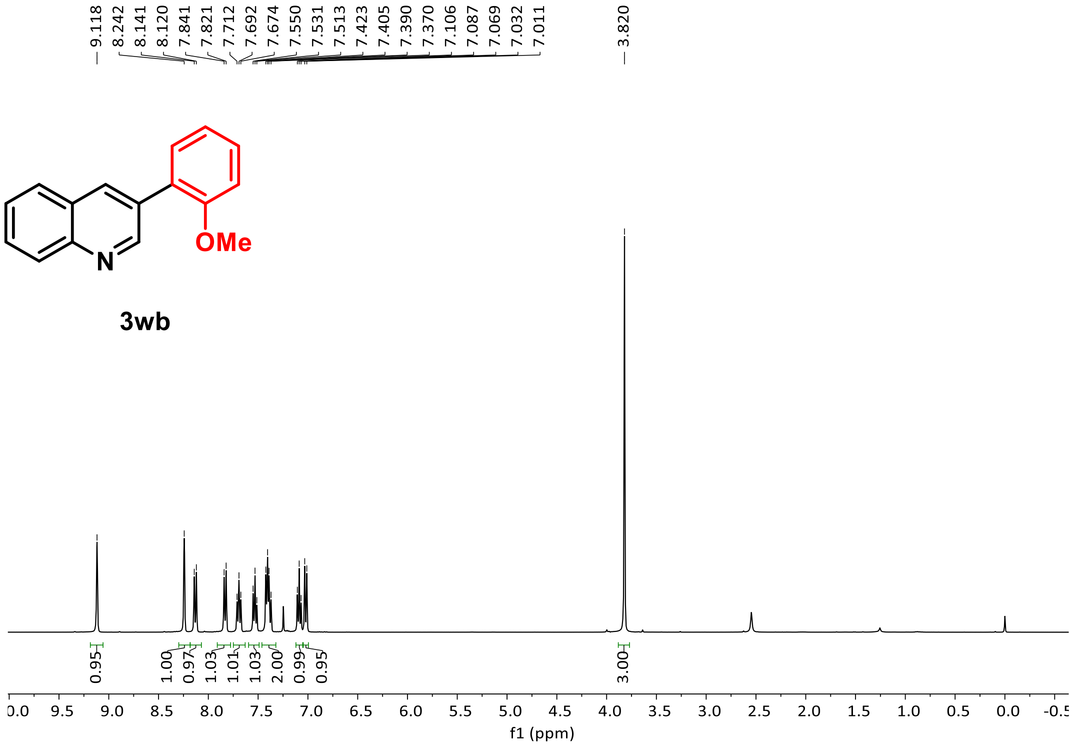

Supplementary Figure 54. ^{1}H NMR of compound **3wb** (400 MHZ, r.t., $CDCl_3$)

^{13}C NMR

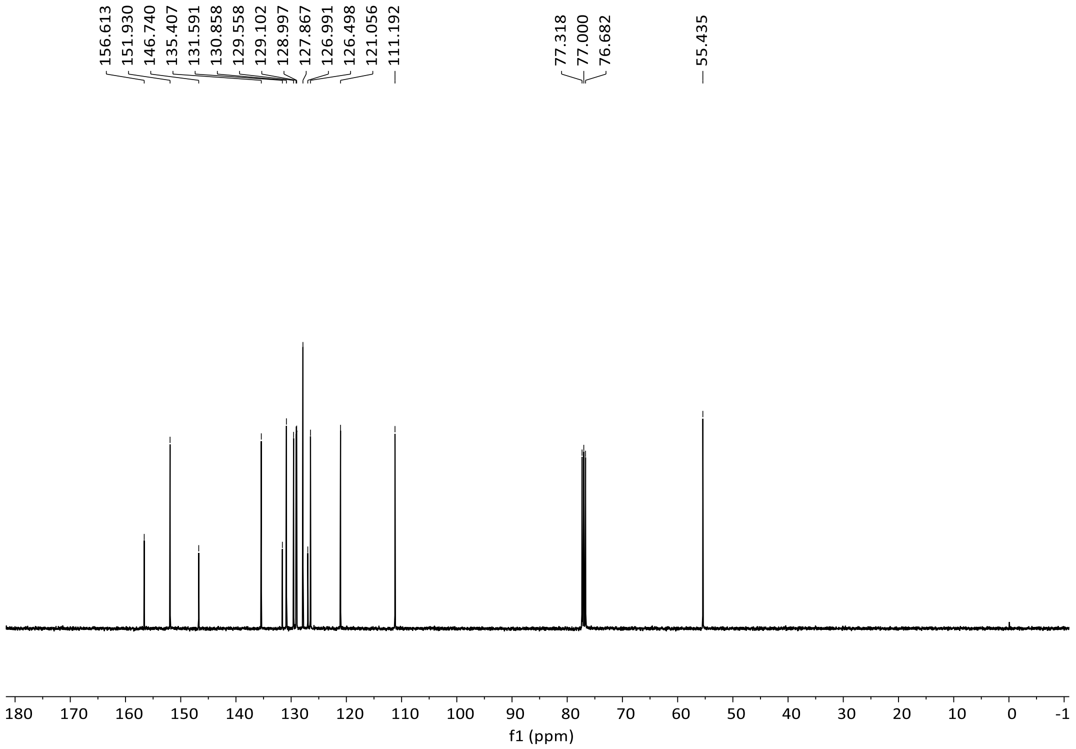

Supplementary Figure 55. ^{13}C NMR of compound **3wb** (101 MHZ, r.t., $CDCl_3$)

^{1}H NMR

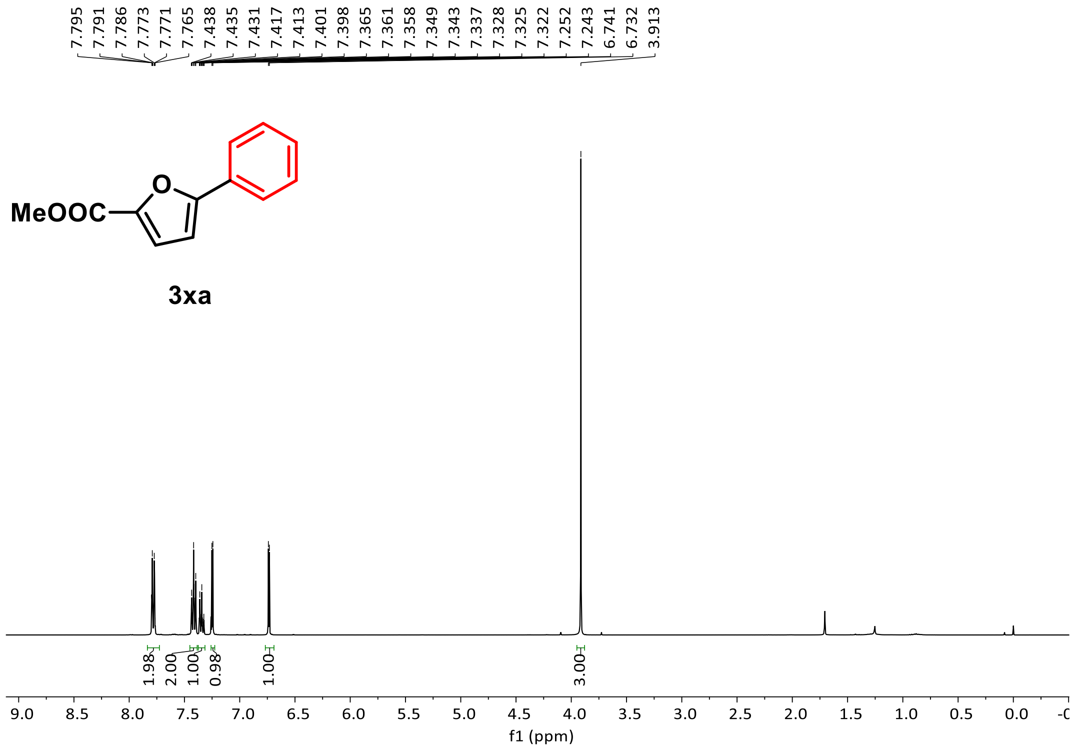

Supplementary Figure 56. ^{1}H NMR of compound **3xa** (400 MHZ, r.t., $CDCl_3$)

^{13}C NMR

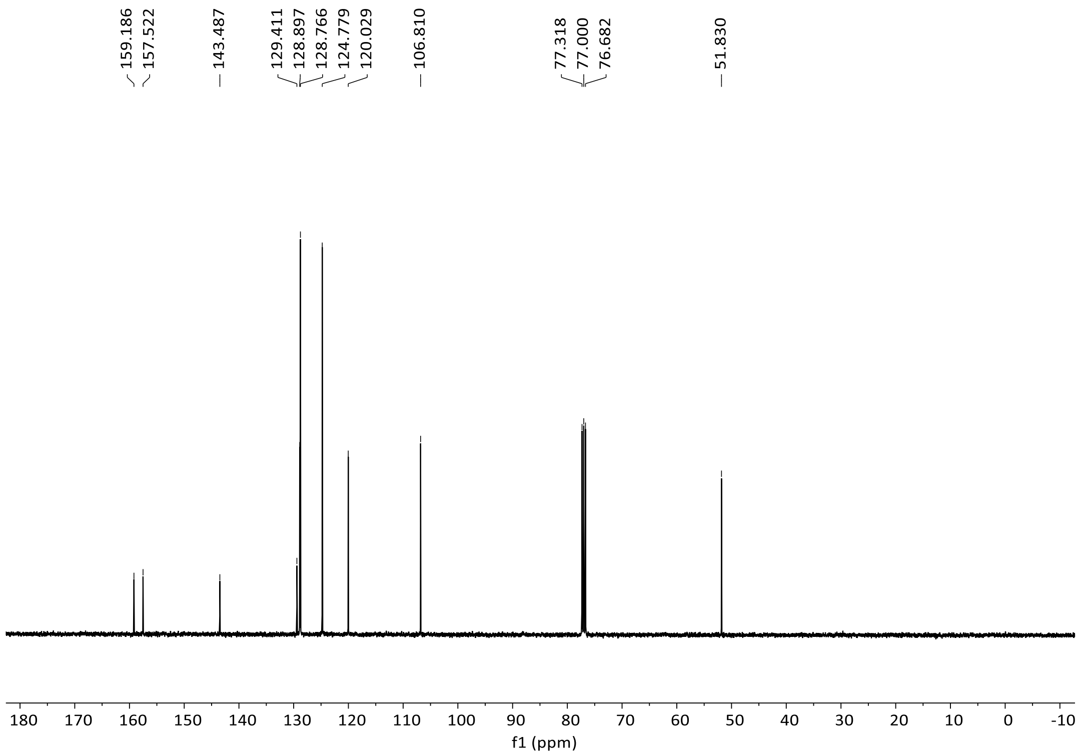

Supplementary Figure 57. ^{13}C NMR of compound **3xa** (101 MHZ, r.t., $CDCl_3$)

^{1}H NMR

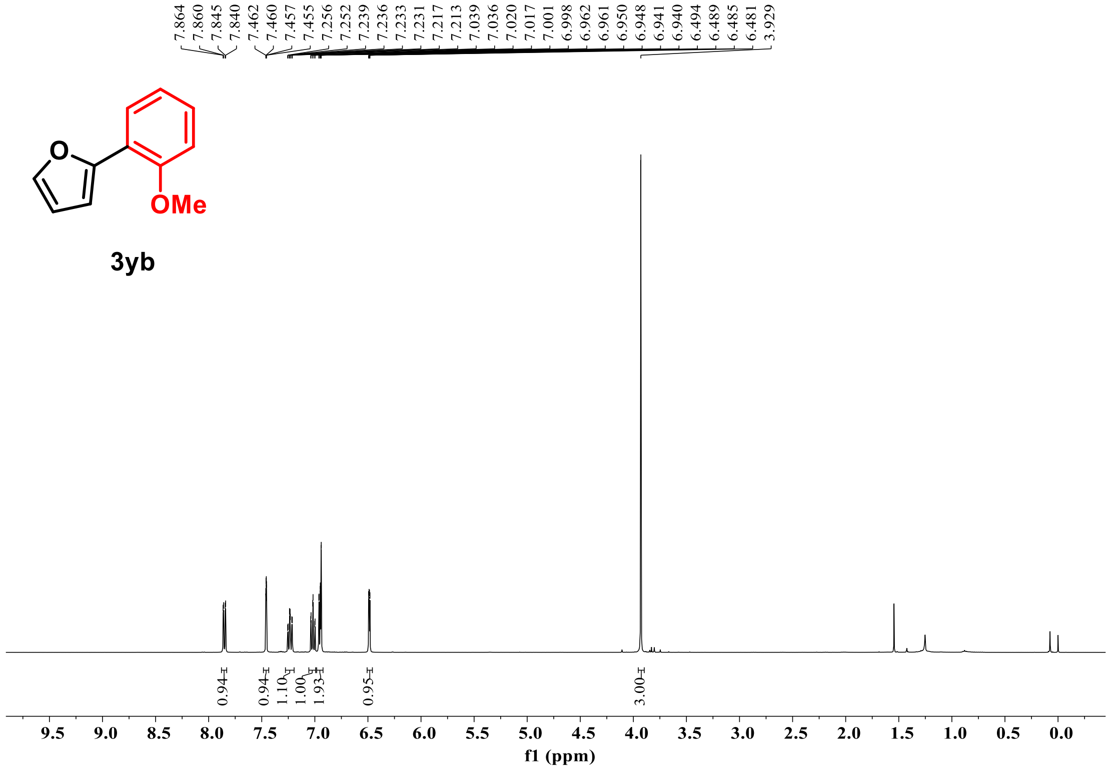

Supplementary Figure 58. ^{1}H NMR of compound **3yb** (400 MHZ, r.t., $CDCl_3$)

^{13}C NMR

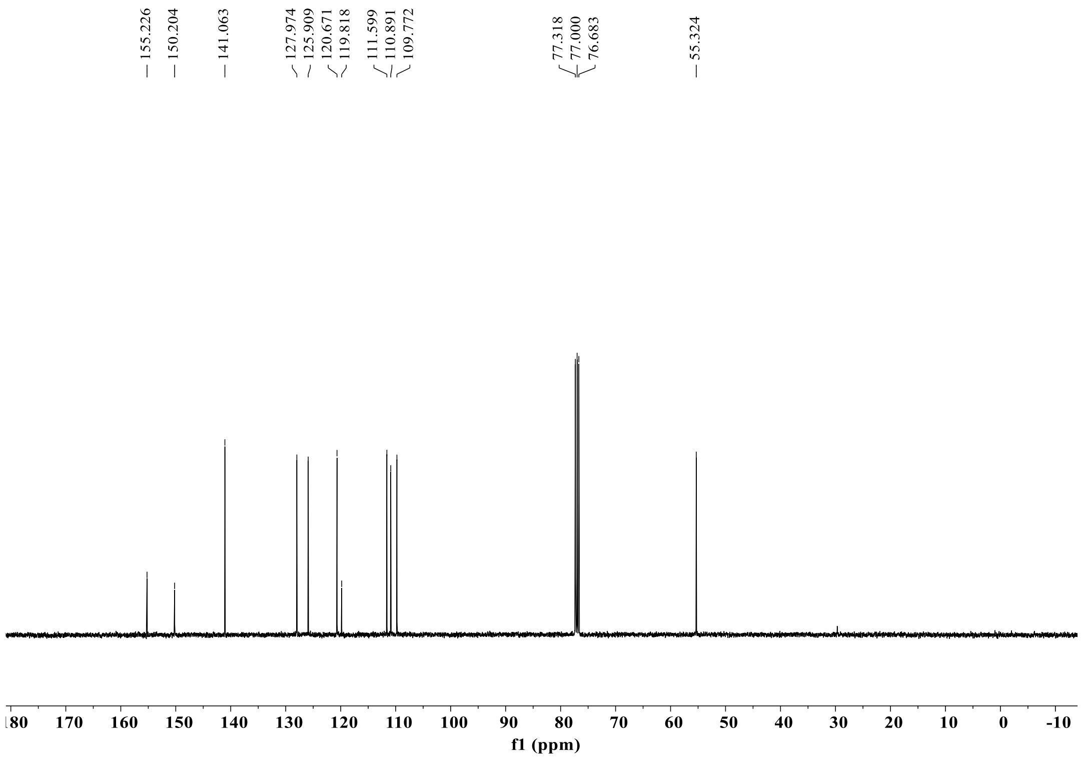

Supplementary Figure 59. ^{13}C NMR of compound **3yb** (101 MHZ, r.t., $CDCl_3$)

^{1}H NMR

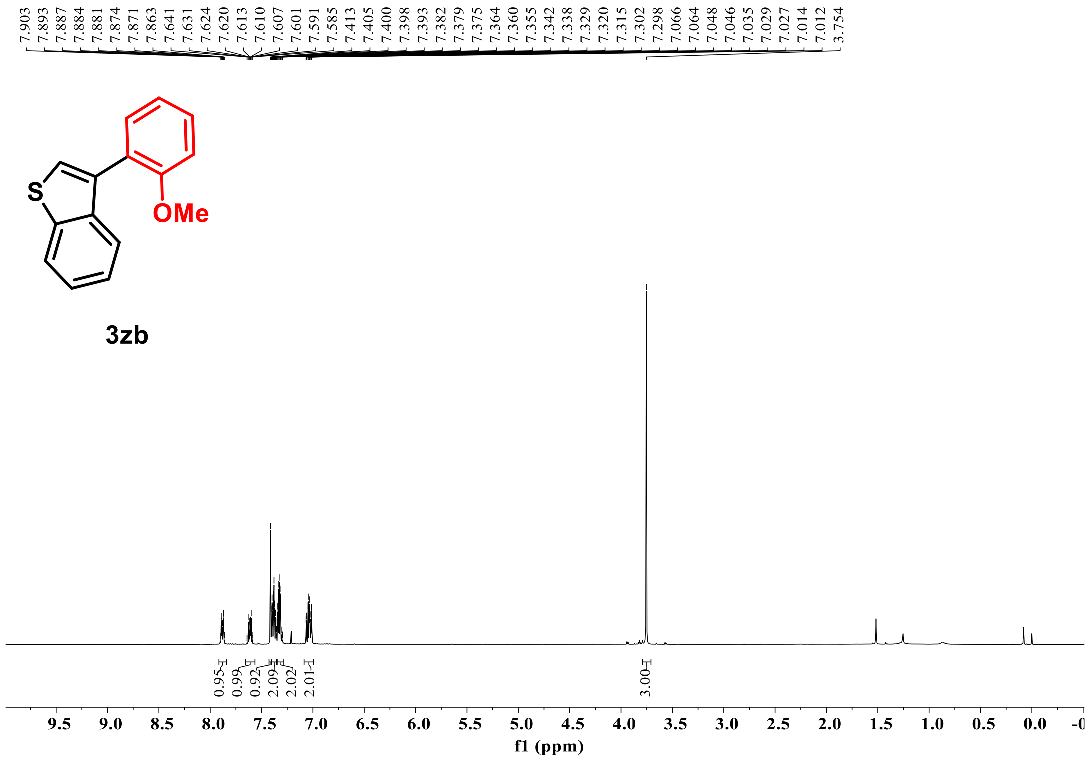

Supplementary Figure 60. ^{1}H NMR of compound **3zb** (400 MHZ, r.t., $CDCl_3$)

^{13}C NMR

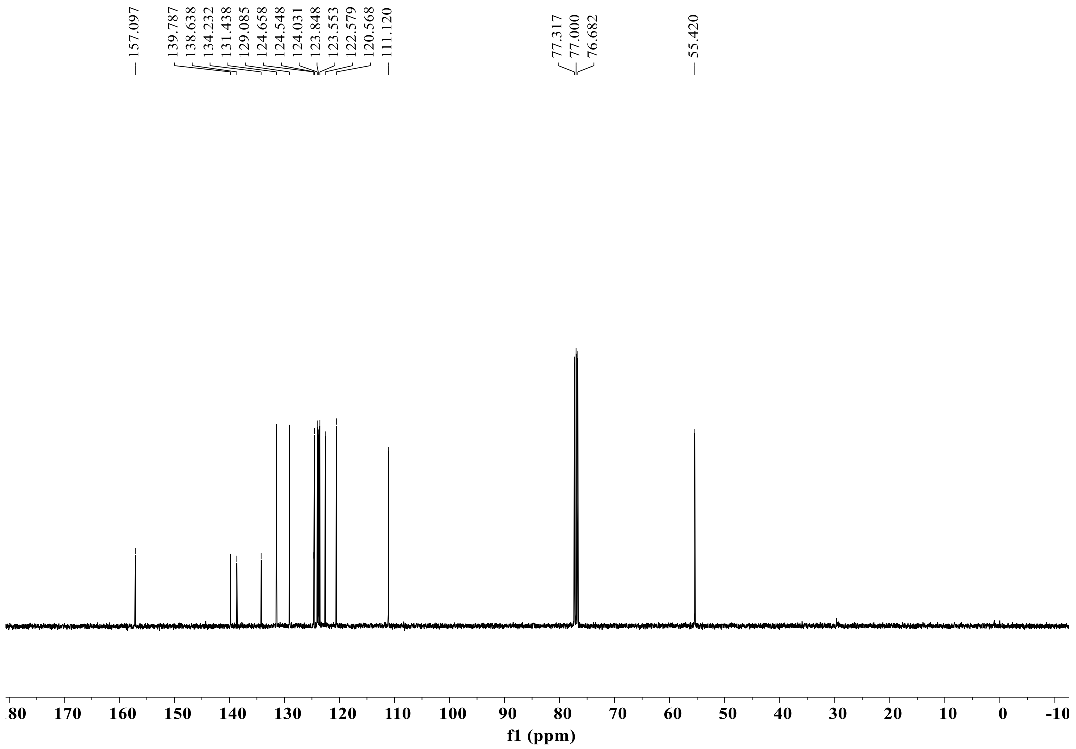

Supplementary Figure 61. ^{13}C NMR of compound **3zb** (101 MHZ, r.t., $CDCl_3$)

^{1}H NMR

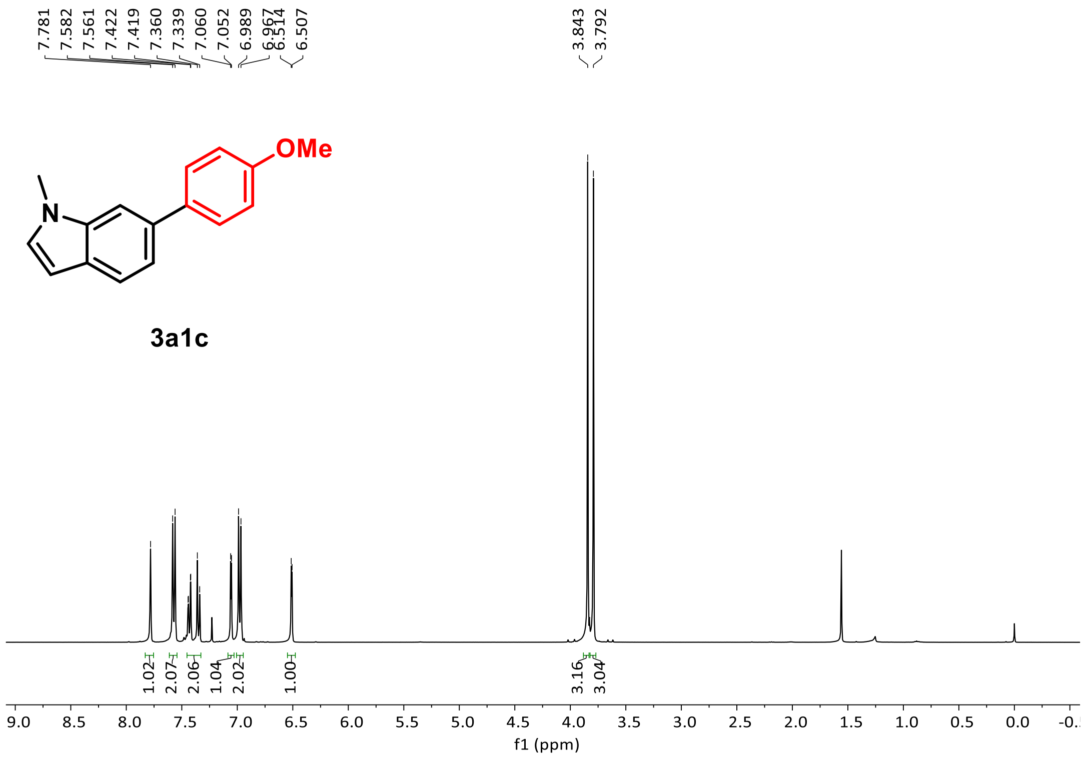

Supplementary Figure 62. ^{1}H NMR of compound **3a1c** (400 MHZ, r.t., $CDCl_3$)

^{13}C NMR

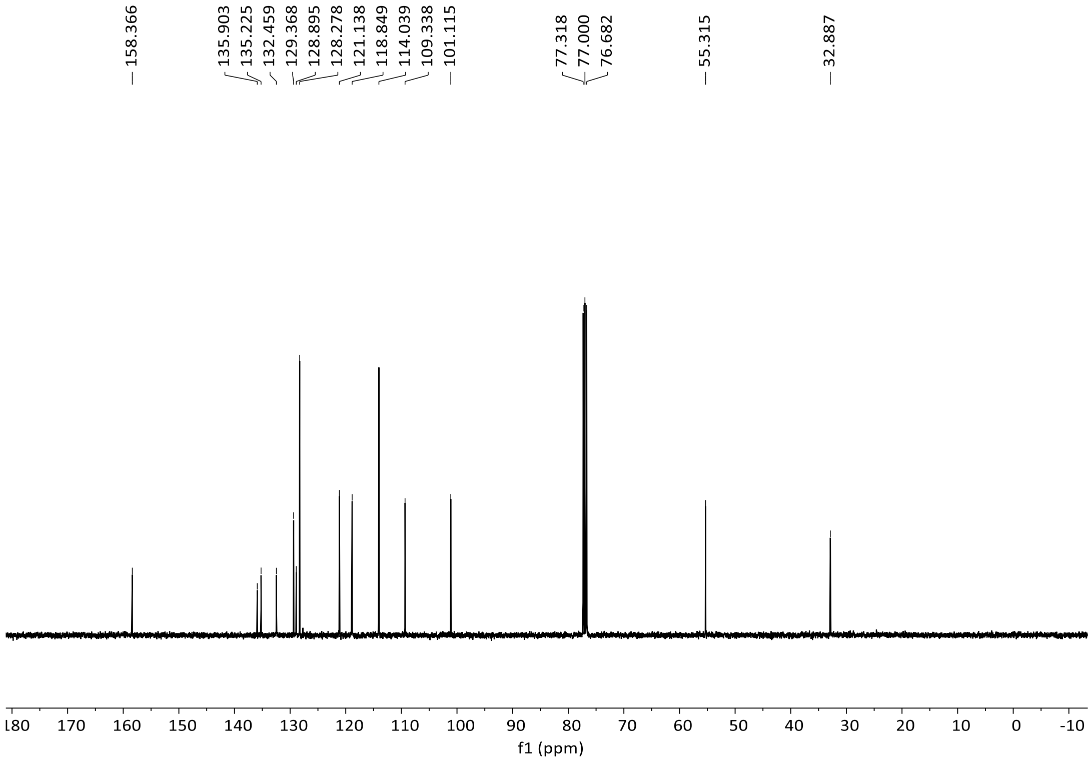

Supplementary Figure 63. ^{13}C NMR of compound **3a1c** (101 MHZ, r.t., $CDCl_3$)

^{1}H NMR

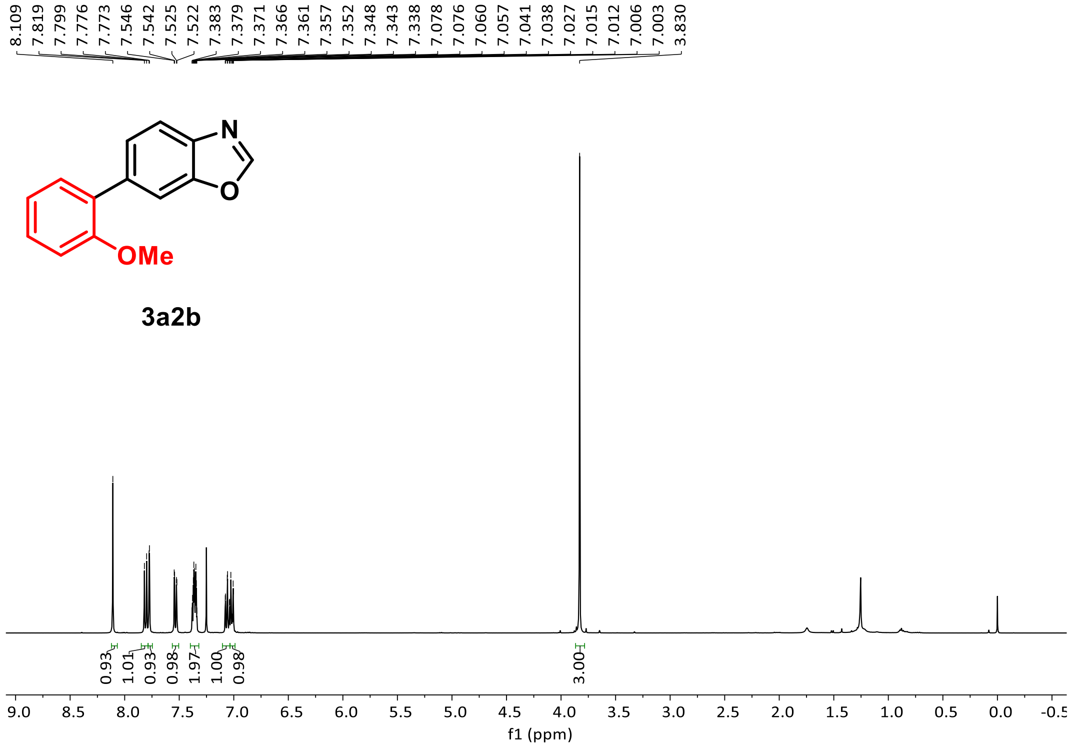

Supplementary Figure 64. ^{1}H NMR of compound **3a2b** (400 MHZ, r.t., $CDCl_3$)

^{13}C NMR

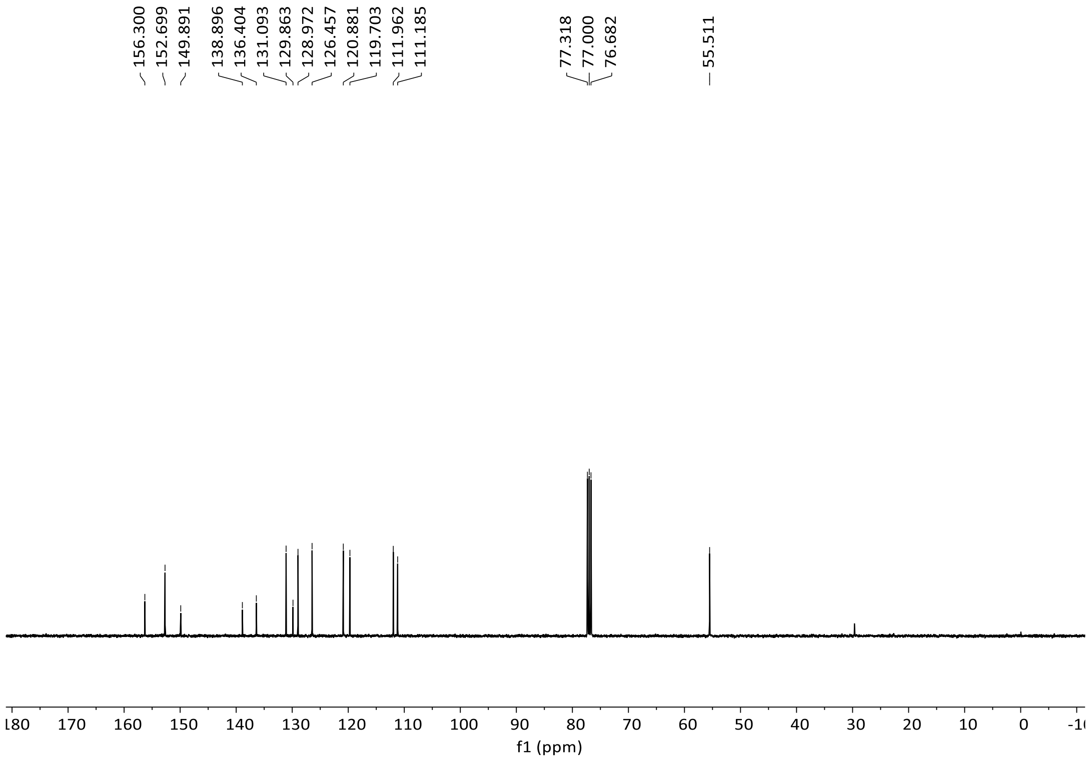

Supplementary Figure 65. ^{13}C NMR of compound **3a2b** (101 MHZ, r.t., $CDCl_3$)

^{1}H NMR

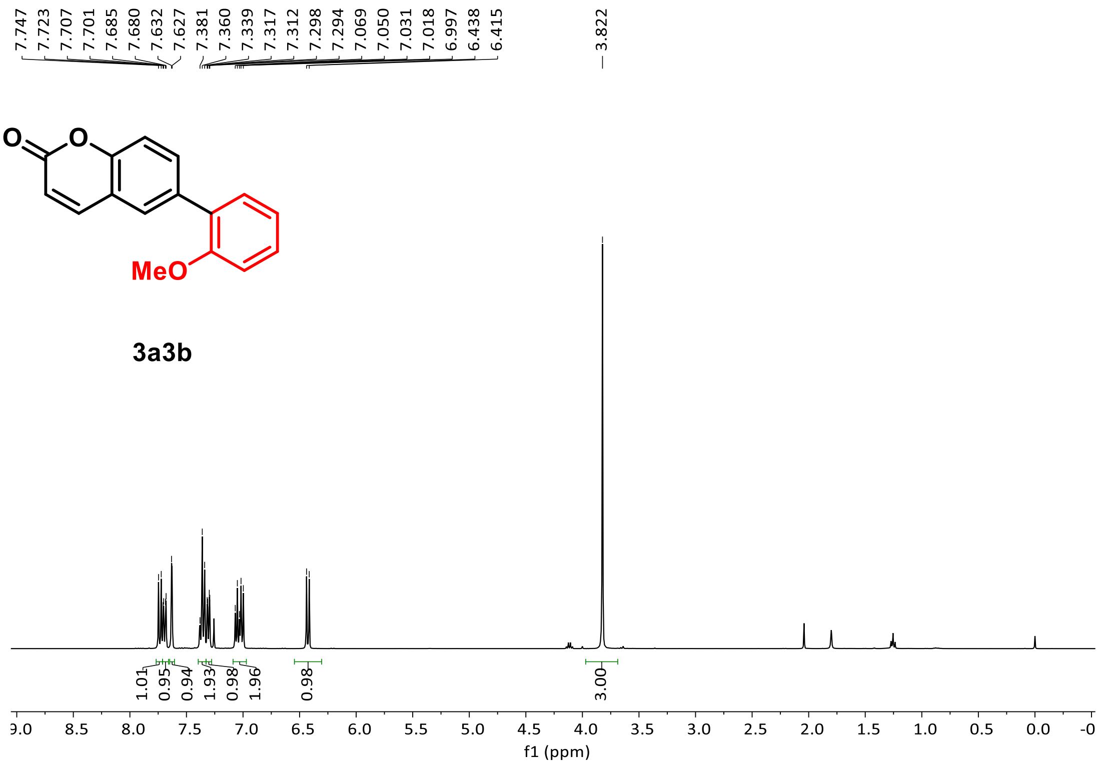

Supplementary Figure 66. ^{1}H NMR of compound **3a3b** (400 MHZ, r.t., $CDCl_3$)

^{13}C NMR

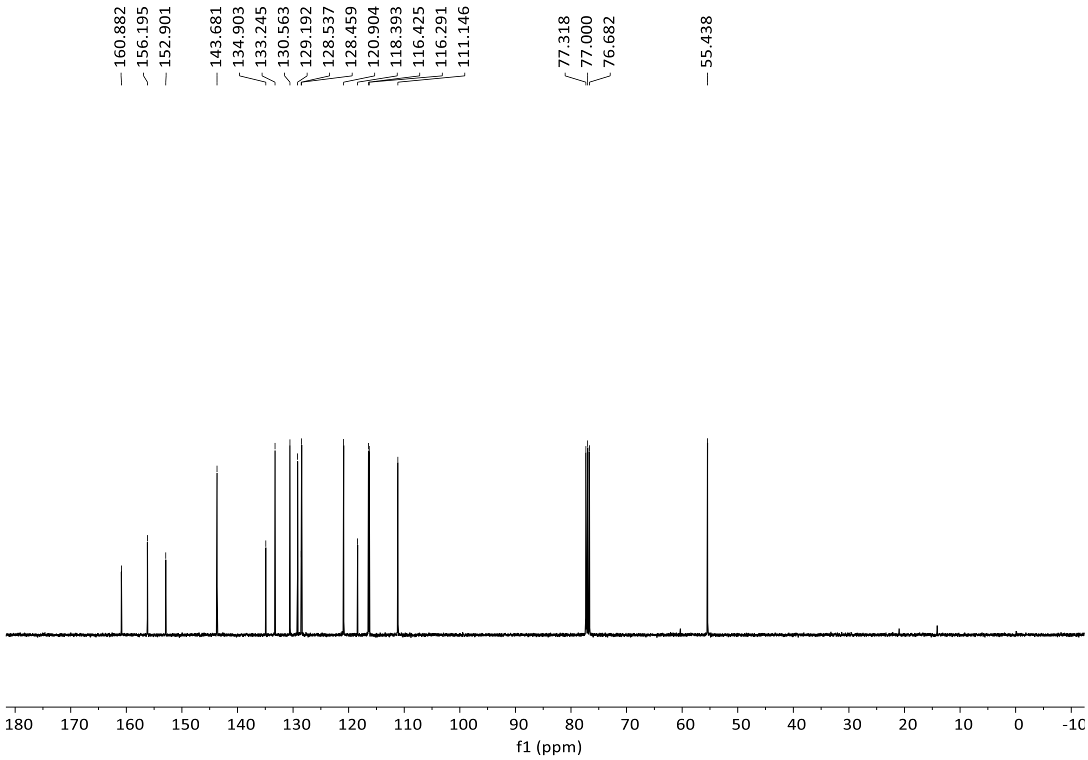

Supplementary Figure 67. ^{13}C NMR of compound **3a3b** (101 MHZ, r.t., $CDCl_3$)

^{1}H NMR

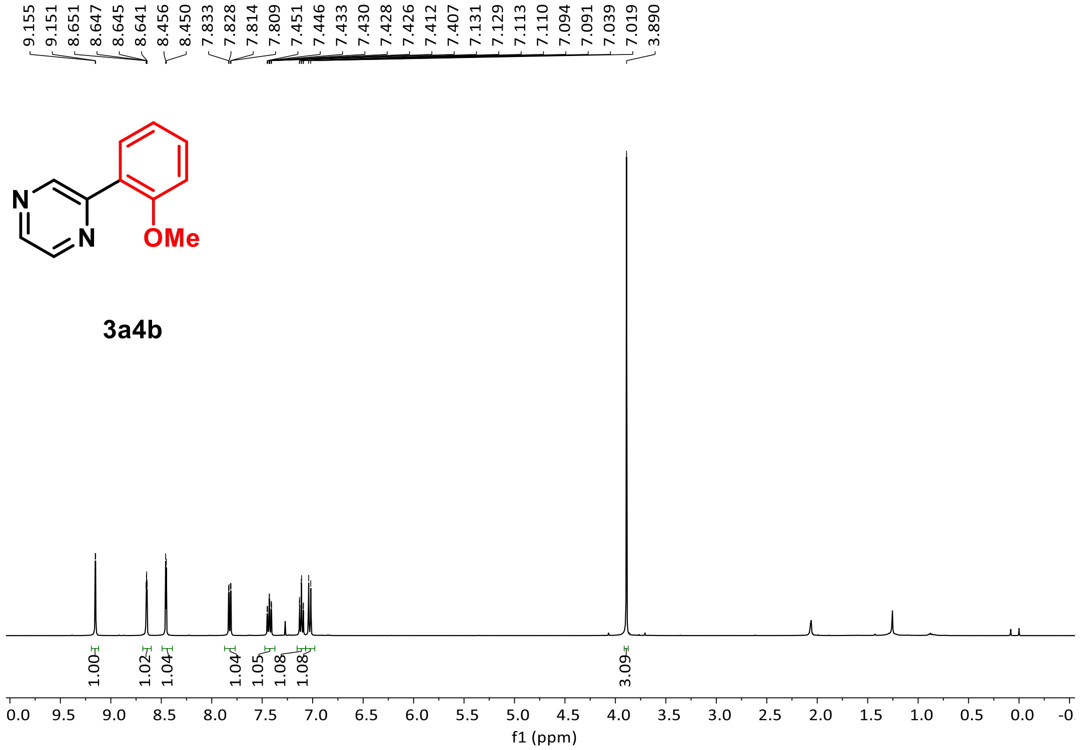

Supplementary Figure 68. ^{1}H NMR of compound **3a4b** (400 MHZ, r.t., $CDCl_3$)

^{13}C NMR

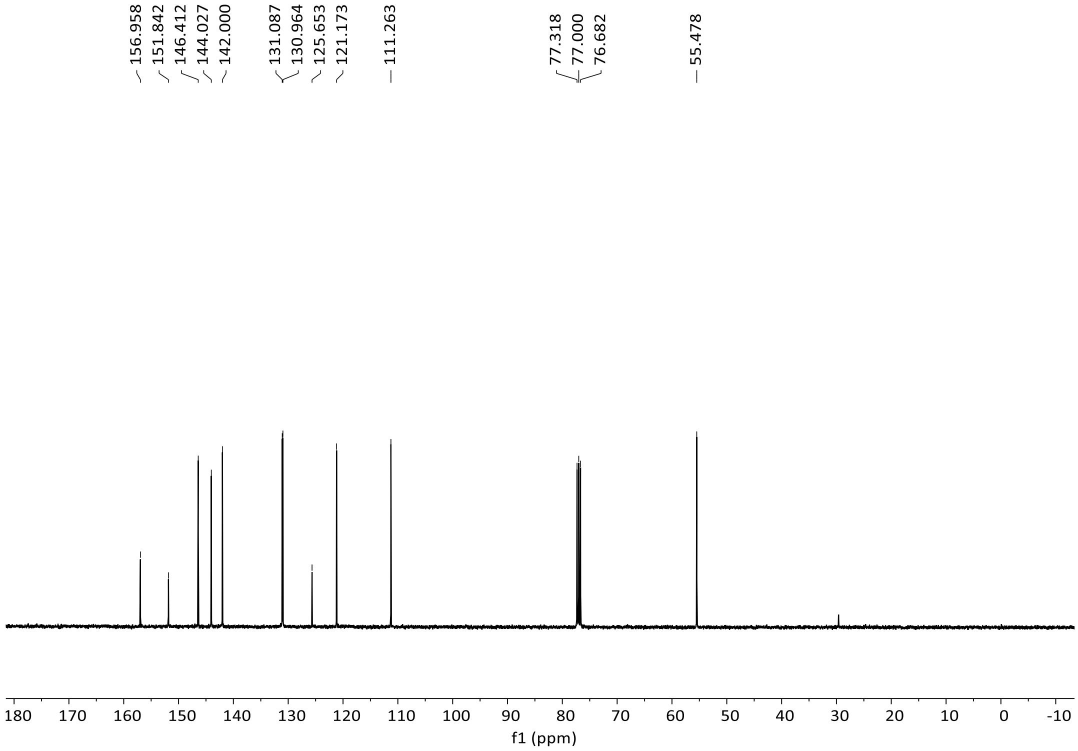

Supplementary Figure 69. ^{13}C NMR of compound **3a4b** (101 MHZ, r.t., $CDCl_3$)

^{1}H NMR

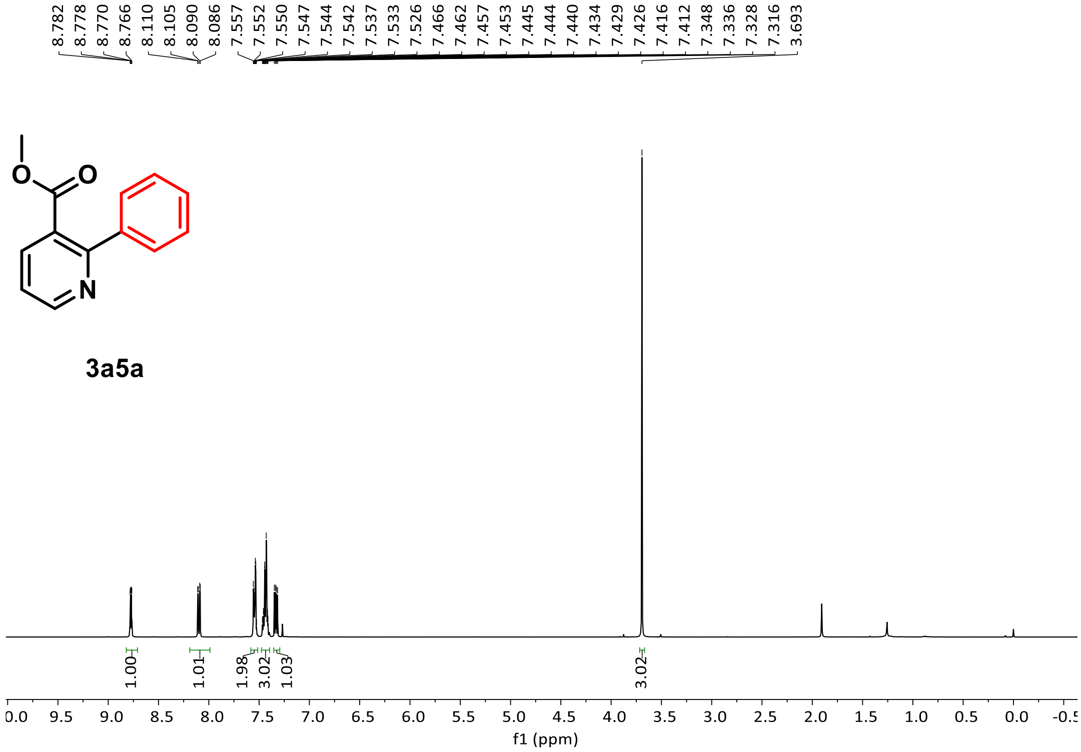

Supplementary Figure 70. ^{1}H NMR of compound **3a5a** (400 MHZ, r.t., $CDCl_3$)

^{13}C NMR

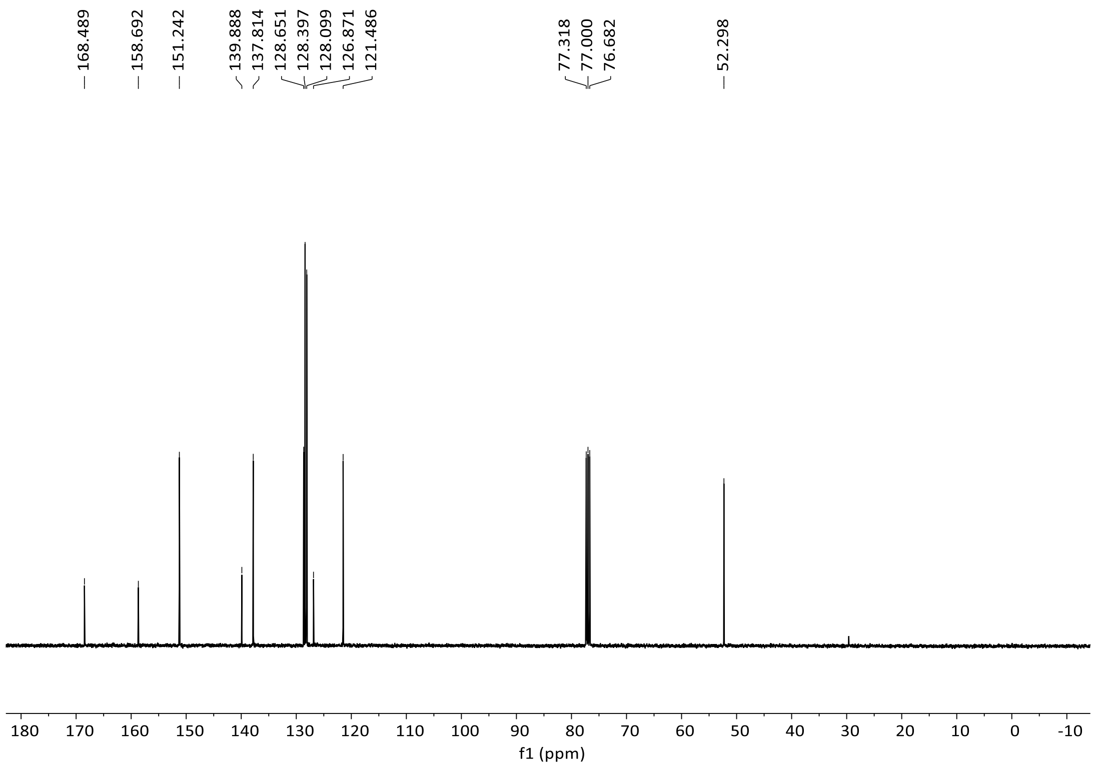

Supplementary Figure 71. ^{13}C NMR of compound **3a5a** (101 MHZ, r.t., $CDCl_3$)

^{1}H NMR

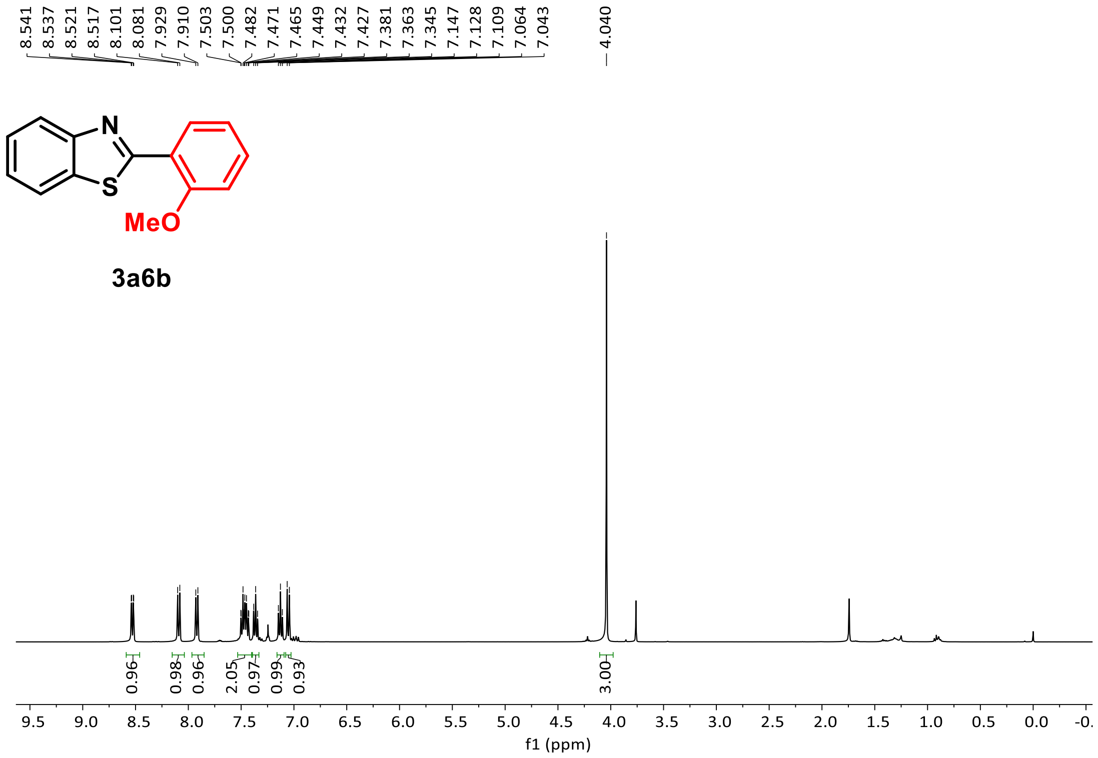

Supplementary Figure 72. ^{1}H NMR of compound **3a6b** (400 MHZ, r.t., $CDCl_3$)

^{13}C NMR

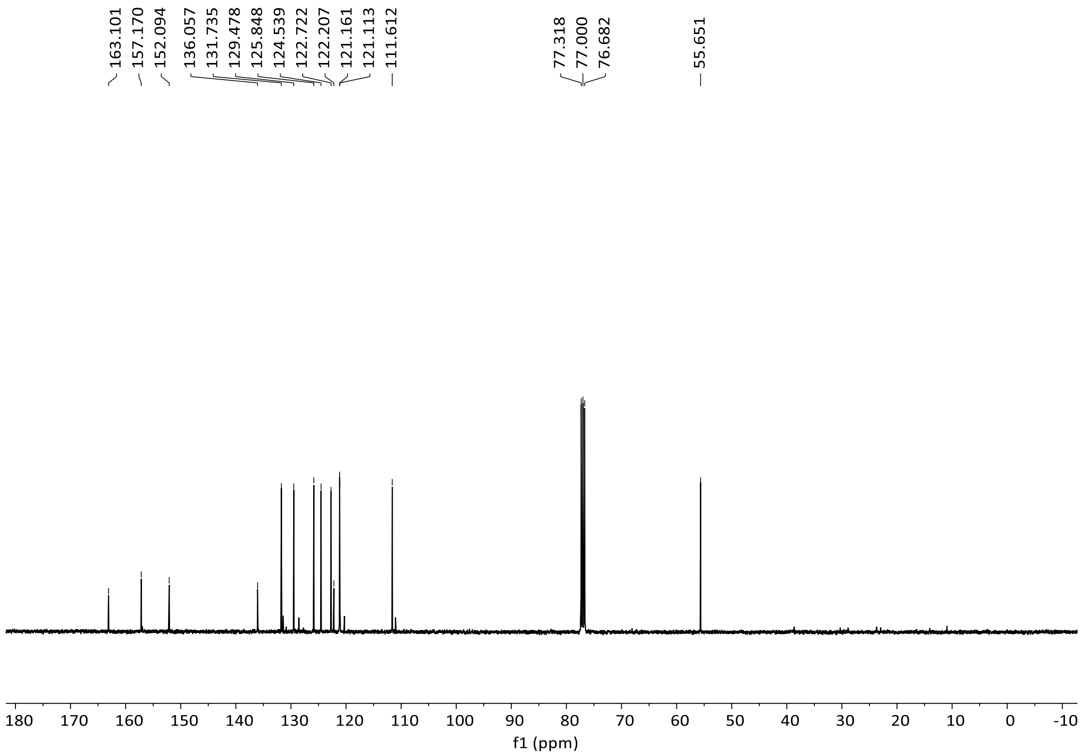

Supplementary Figure 73. ^{13}C NMR of compound 3**a6b** (101 MHZ, r.t., $CDCl_3$)

^{1}H NMR

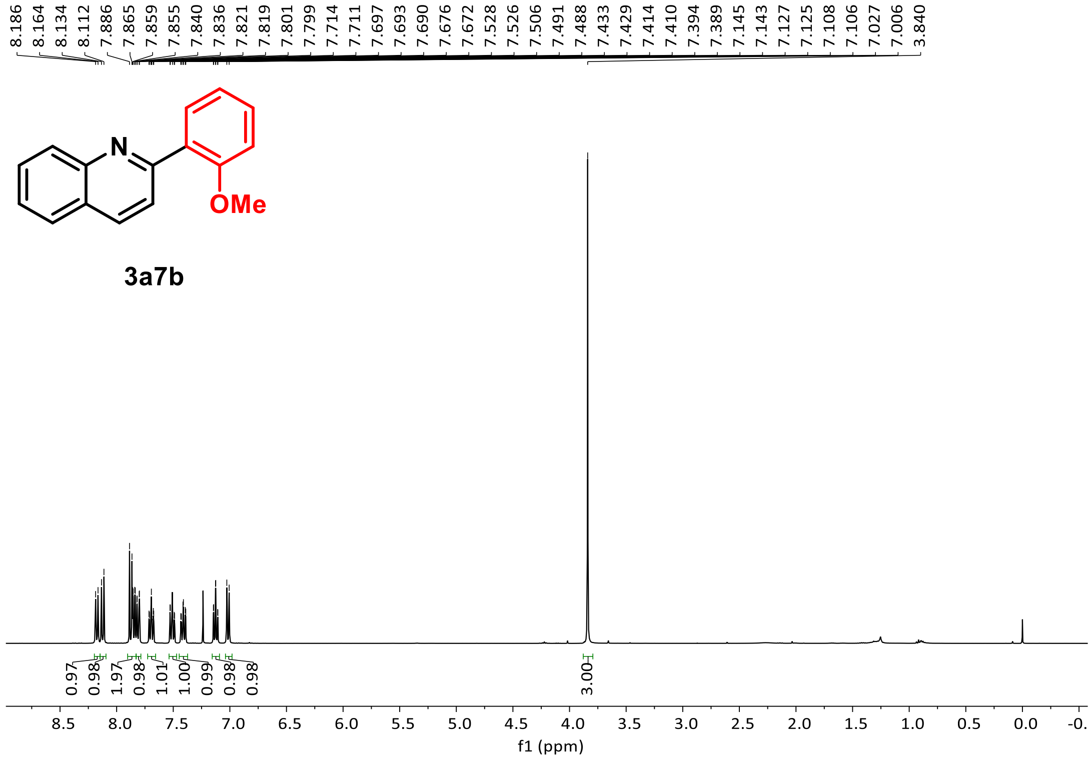

Supplementary Figure 74. ^{1}H NMR of compound **3a7b** (400 MHZ, r.t., $CDCl_3$)

^{13}C NMR

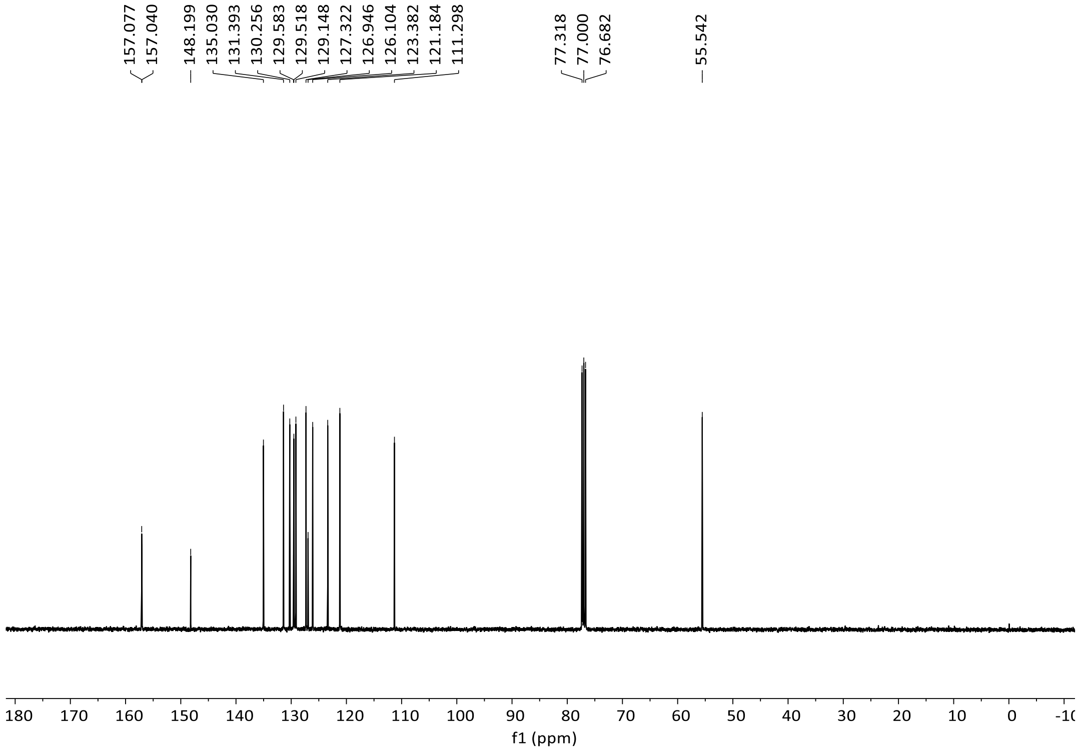

Supplementary Figure 75. ^{13}C NMR of compound **3a7b** (101 MHZ, r.t., $CDCl_3$)

^{1}H NMR

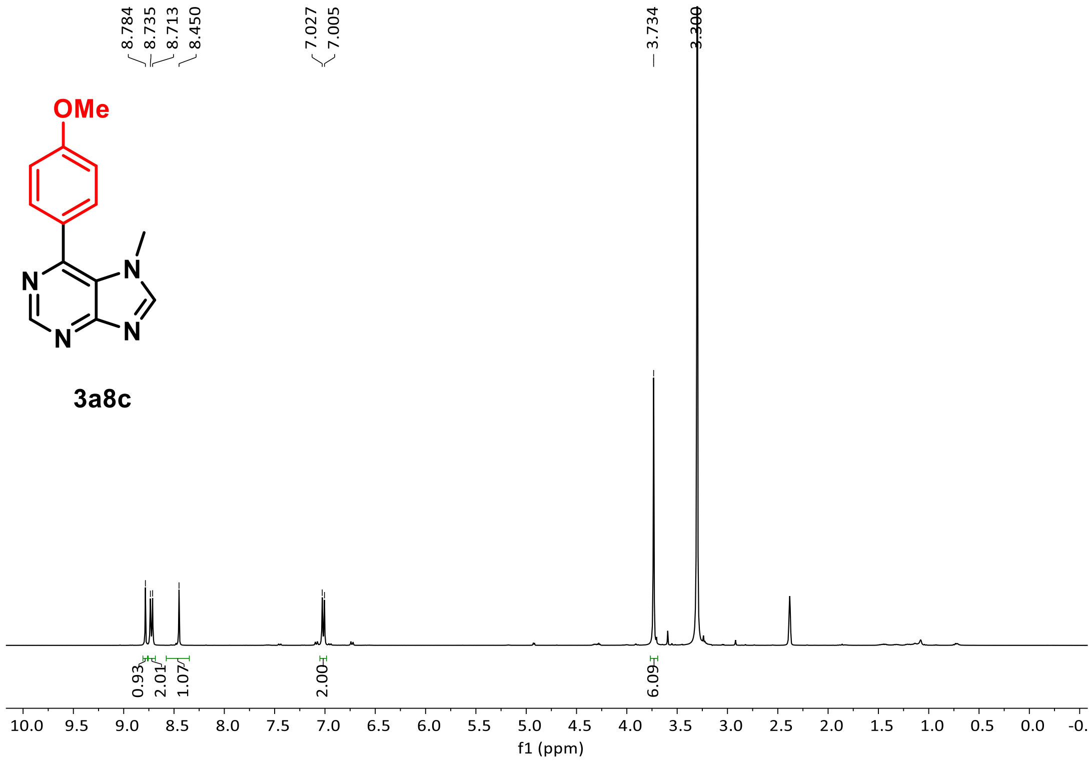

Supplementary Figure 76. ^{1}H NMR of compound 3**a8c** (400 MHZ, r.t., $CDCl_3$)

^{13}C NMR

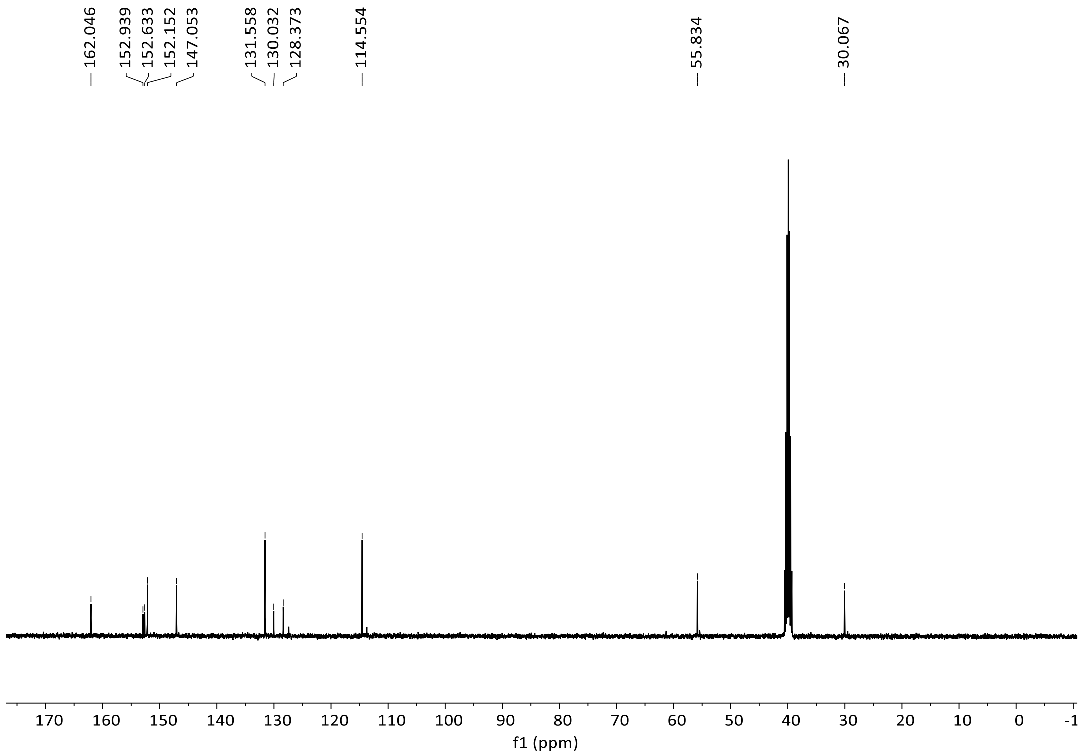

Supplementary Figure 77. ^{13}C NMR of compound **3a8c** (101 MHZ, r.t., $CDCl_3$)

^{1}H NMR

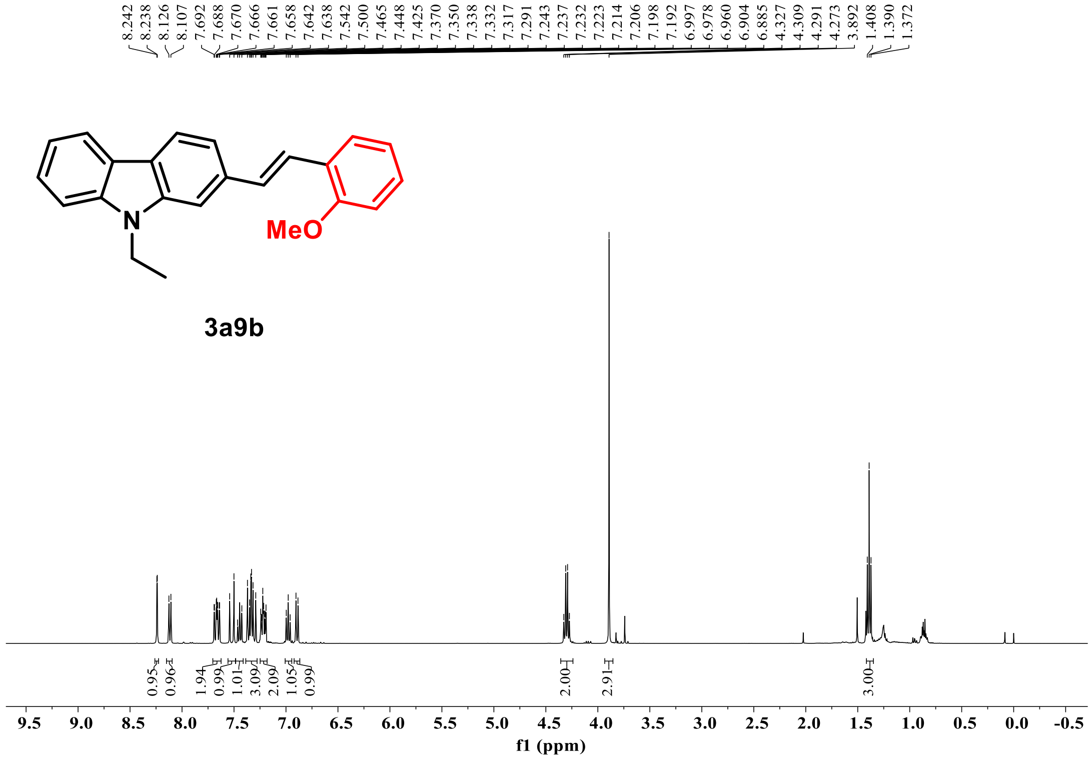

Supplementary Figure 78. ^{1}H NMR of compound **3a9b** (400 MHZ, r.t., $CDCl_3$)

^{13}C NMR

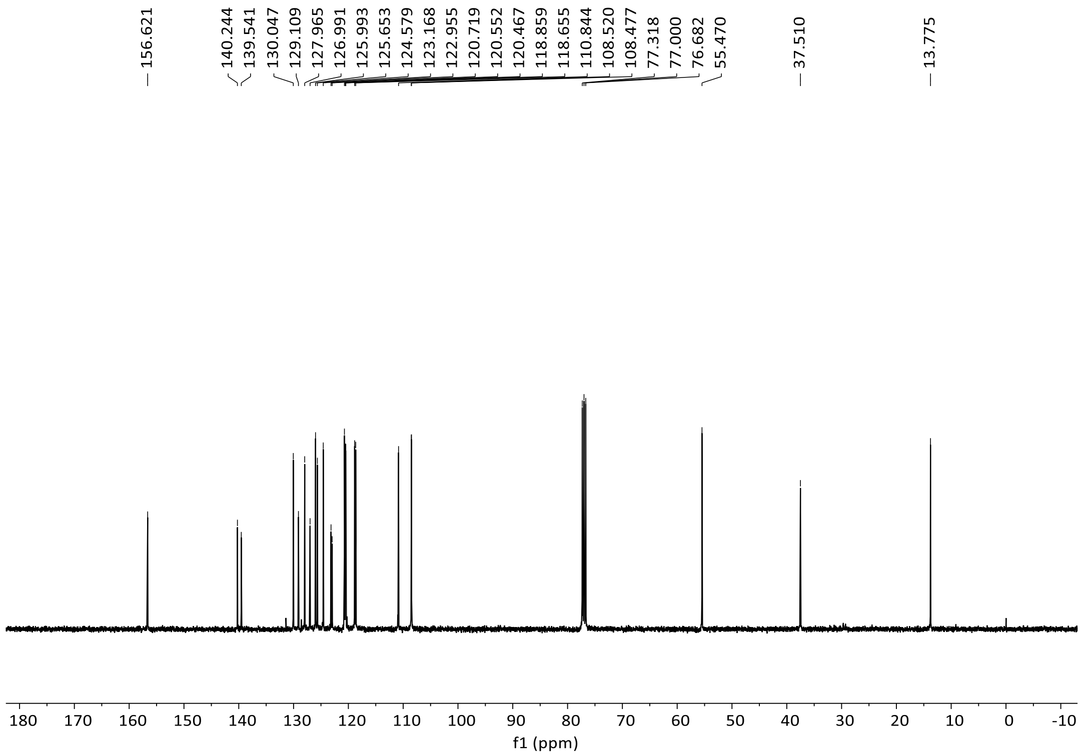

Supplementary Figure 79. ^{13}C NMR of compound **3a9b** (101 MHZ, r.t., $CDCl_3$)

^{1}H NMR

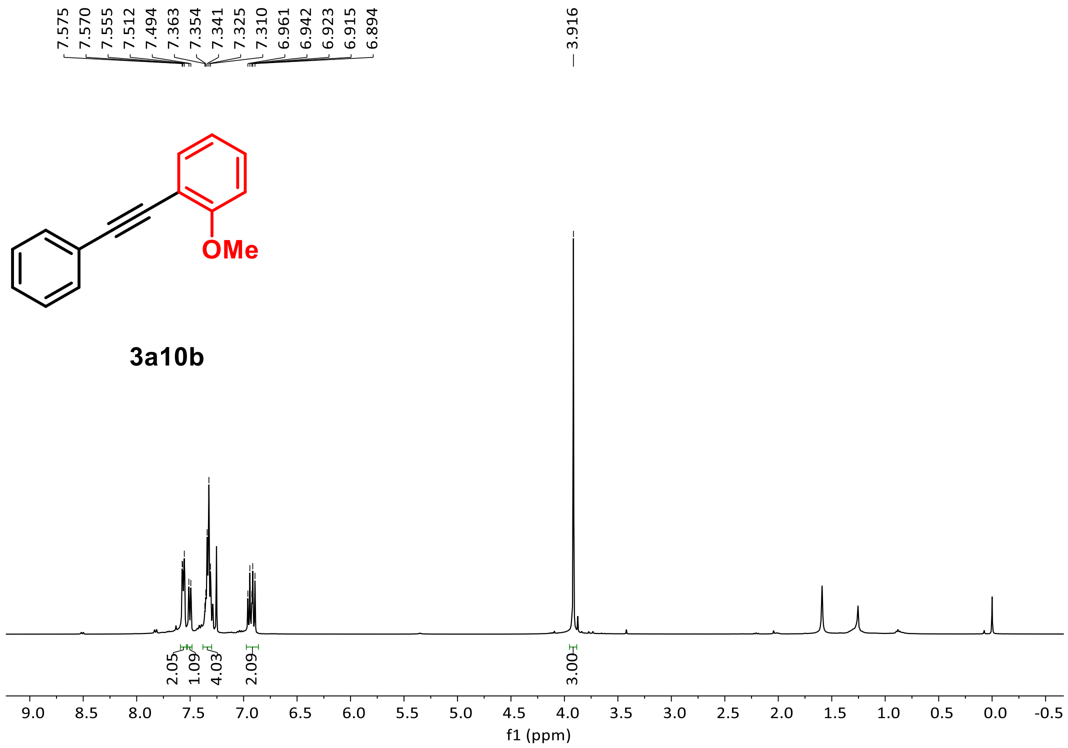

Supplementary Figure 80. ^{1}H NMR of compound **3a10b** (400 MHZ, r.t., $CDCl_3$)

^{13}C NMR

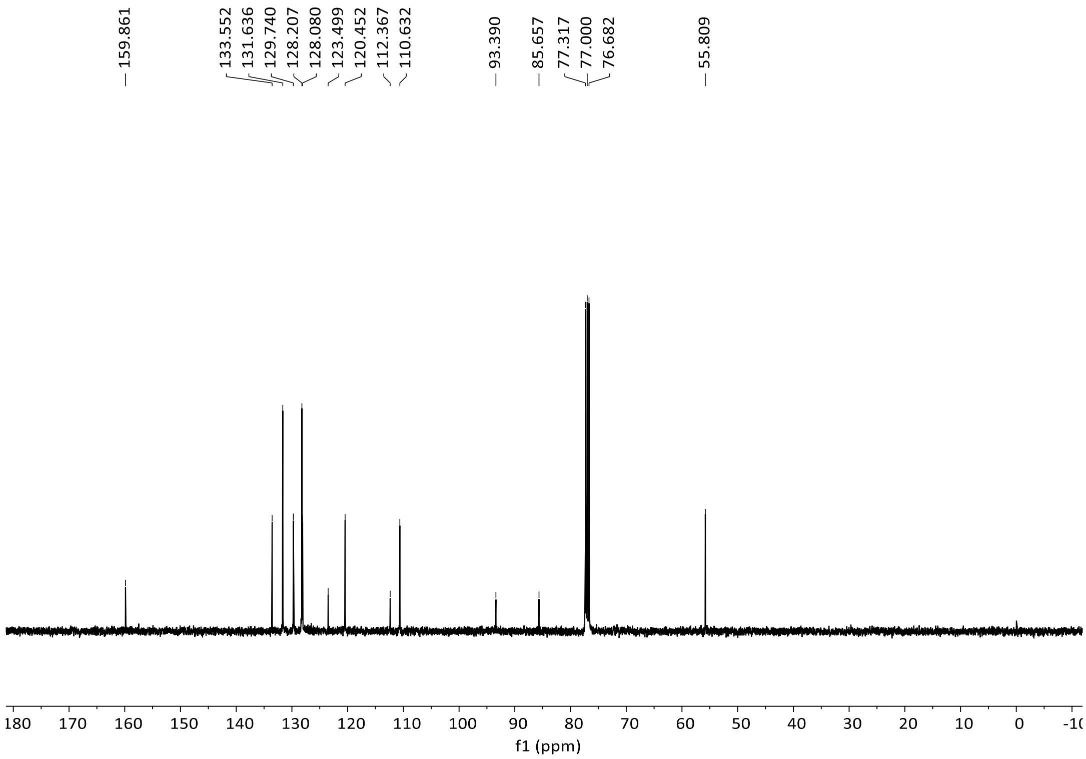

Supplementary Figure 81. ^{13}C NMR of compound **3a10b** (101 MHZ, r.t., $CDCl_3$)

^{1}H NMR

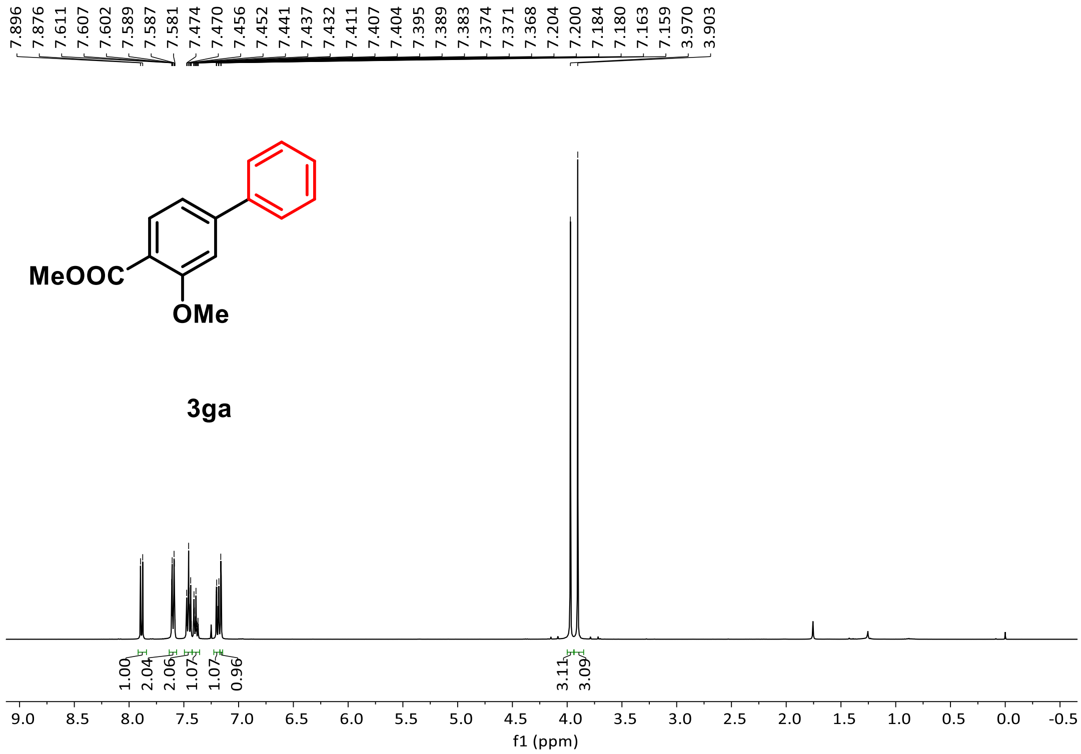

Supplementary Figure 82. ^{1}H NMR of compound **3ga** (400 MHZ, r.t., $CDCl_3$)

^{13}C NMR

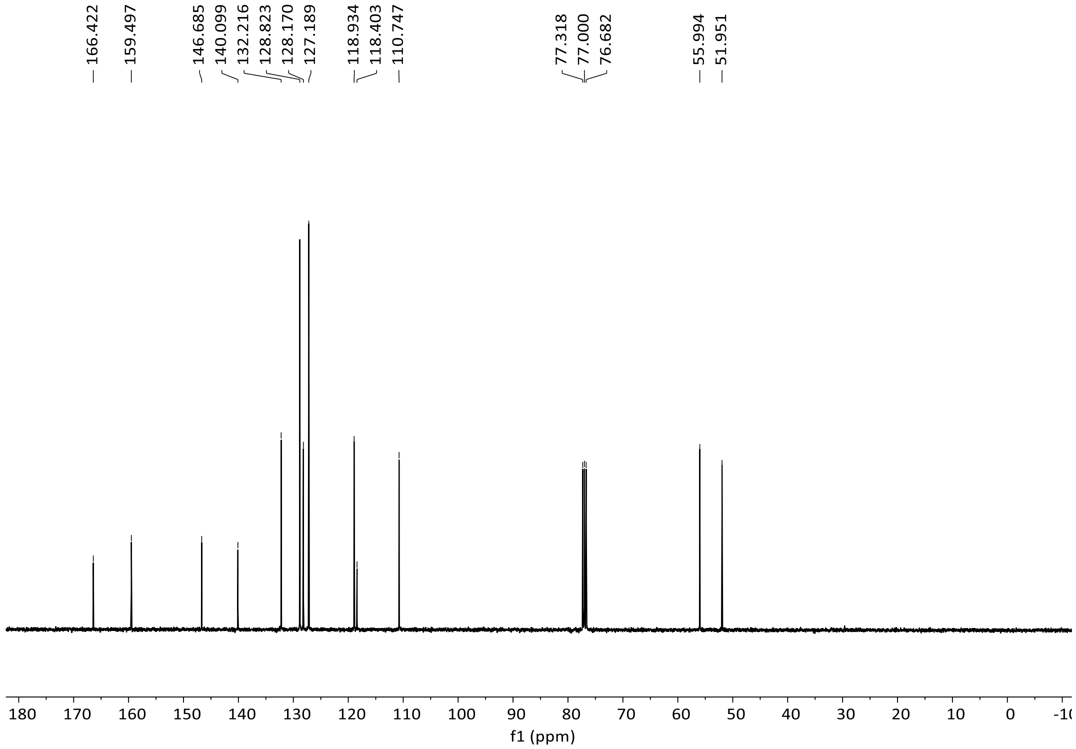

Supplementary Figure 83. ^{13}C NMR of compound **3ga** (101 MHZ, r.t., $CDCl_3$)

^{1}H NMR

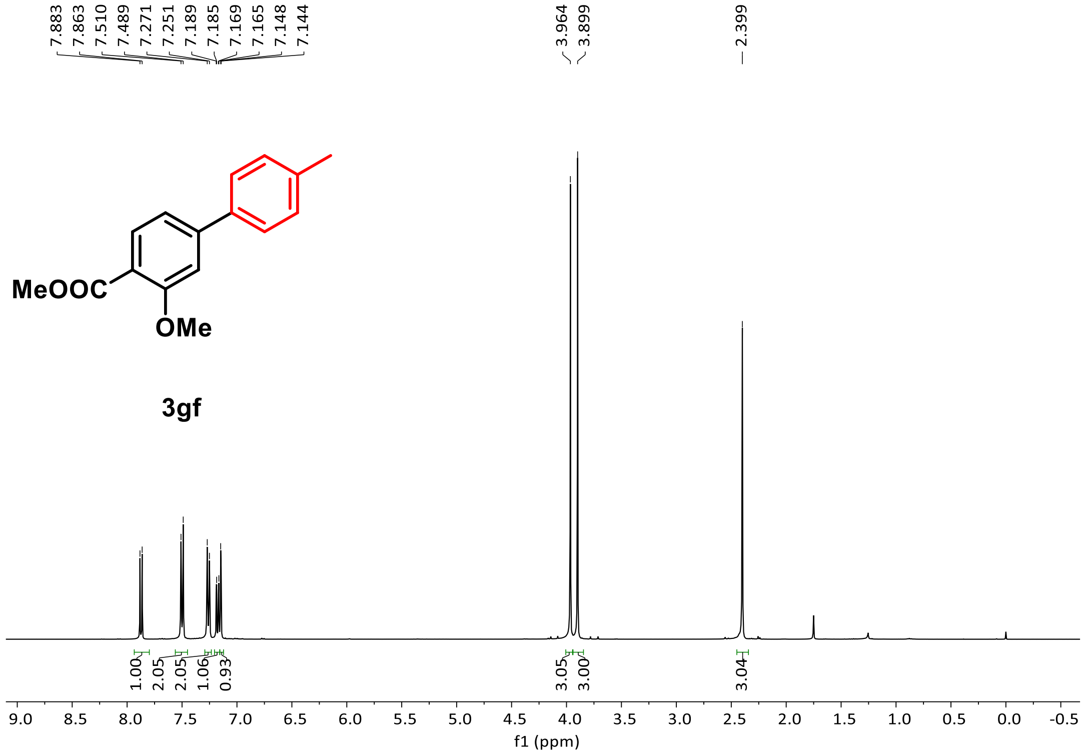

Supplementary Figure 84. ^{1}H NMR of compound **3gf** (400 MHZ, r.t., $CDCl_3$)

^{13}C NMR

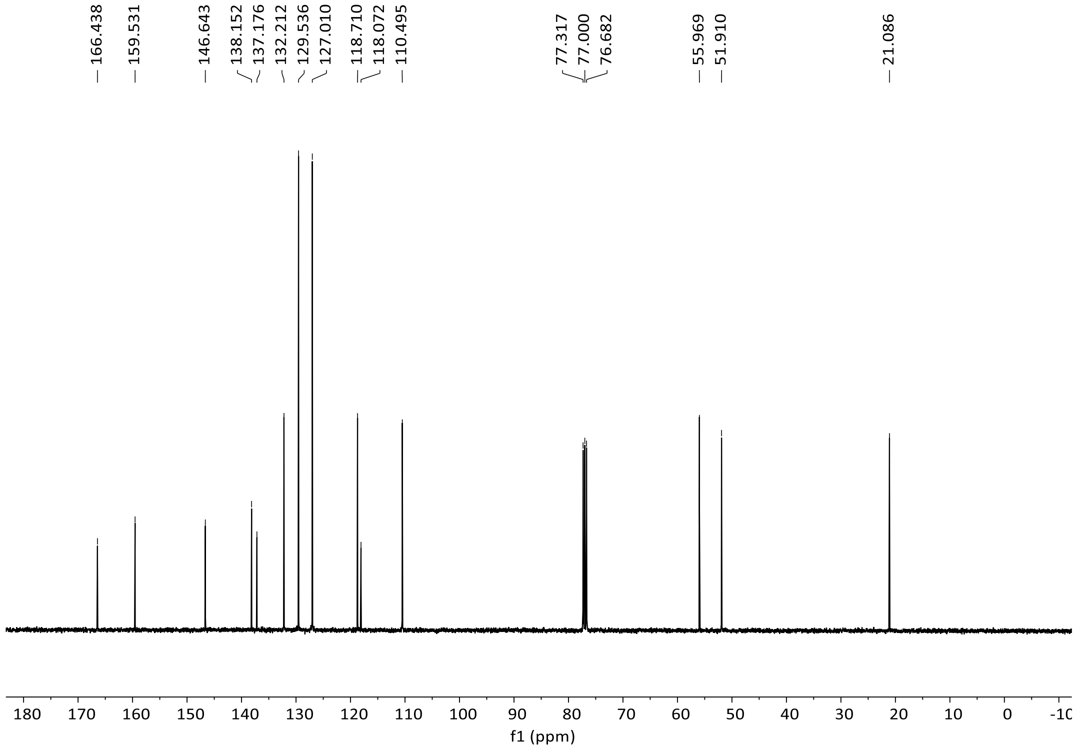

Supplementary Figure 85. ^{13}C NMR of compound **3gf** (101 MHZ, r.t., $CDCl_3$)

^{1}H NMR

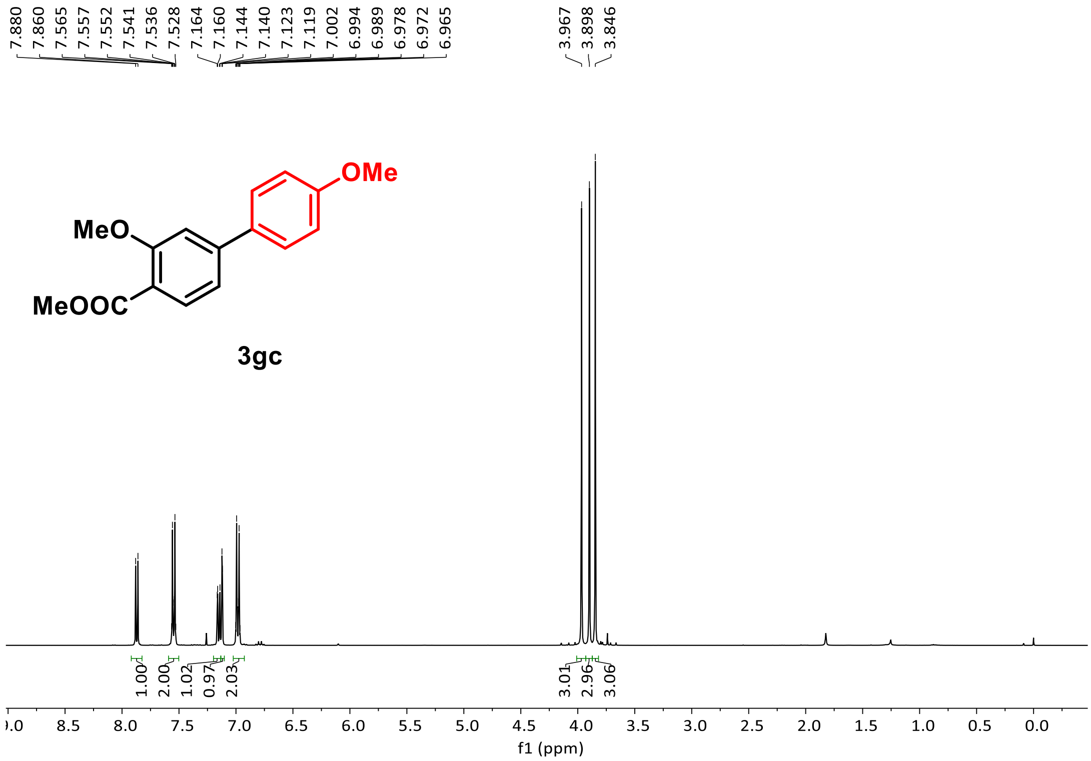

Supplementary Figure 86. ^{1}H NMR of compound **3gc** (400 MHZ, r.t., $CDCl_3$)

^{13}C NMR

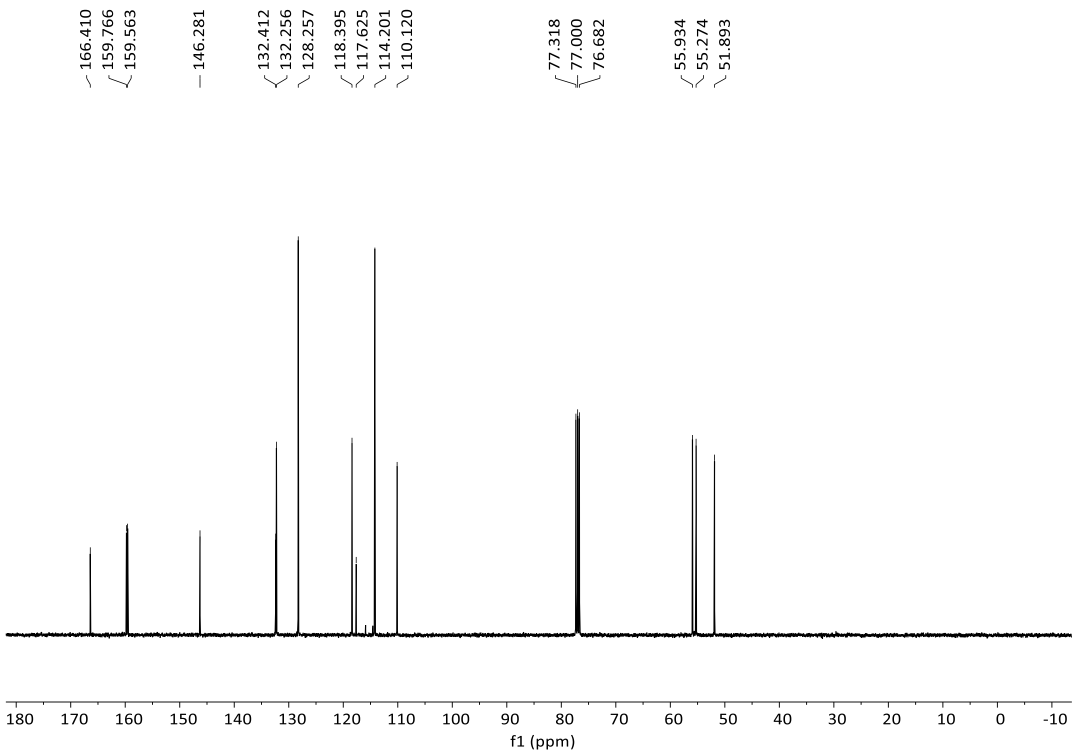

Supplementary Figure 87. ^{13}C NMR of compound **3gc** (101 MHZ, r.t., $CDCl_3$)

^{1}H NMR

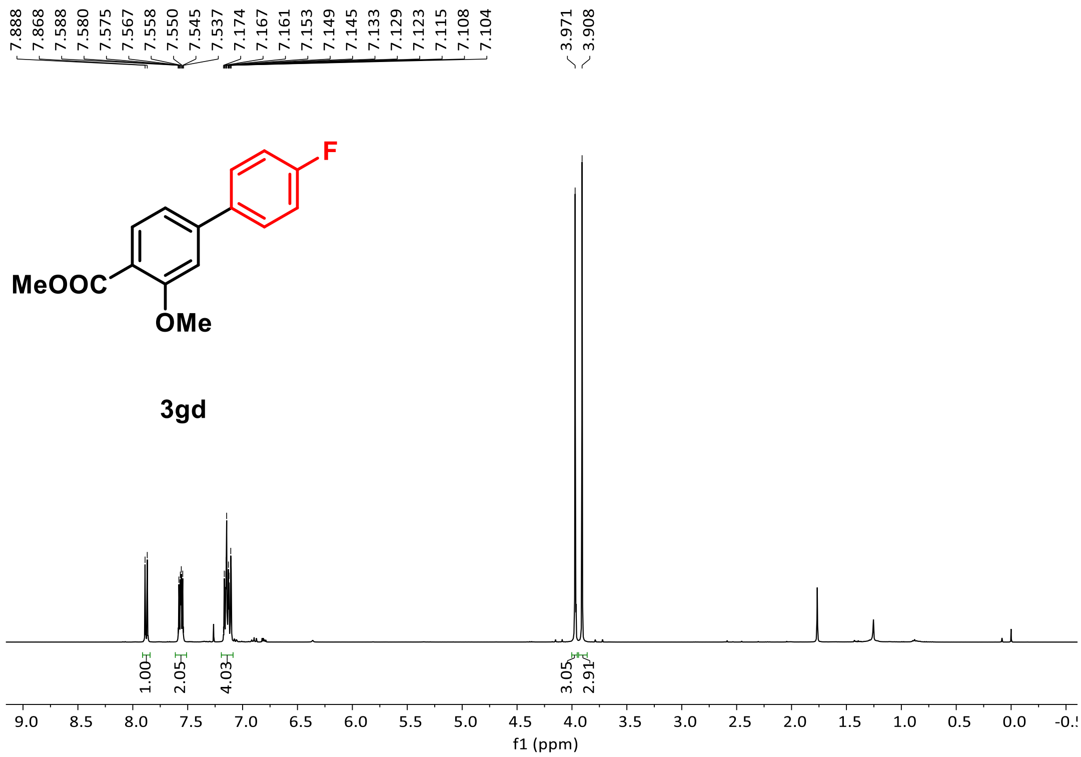

Supplementary Figure 88. ^{1}H NMR of compound **3gd** (400 MHZ, r.t., $CDCl_3$)

^{13}C NMR

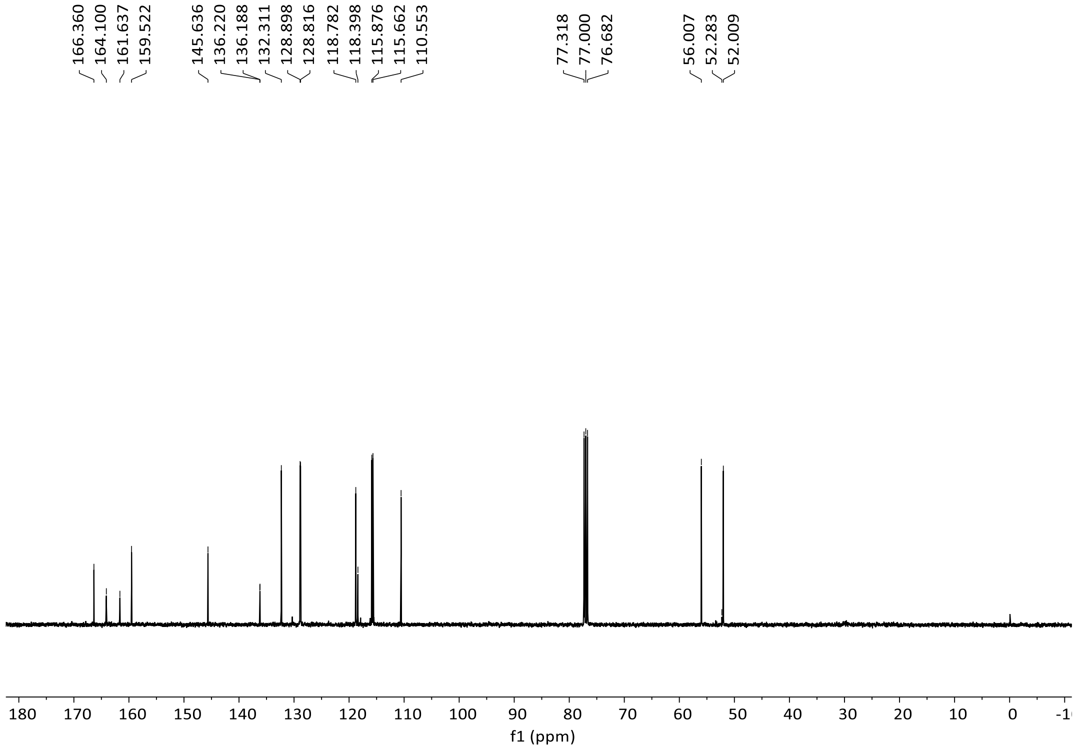

Supplementary Figure 89. ^{13}C NMR of compound **3gd** (101 MHZ, r.t., $CDCl_3$)

^{1}H NMR

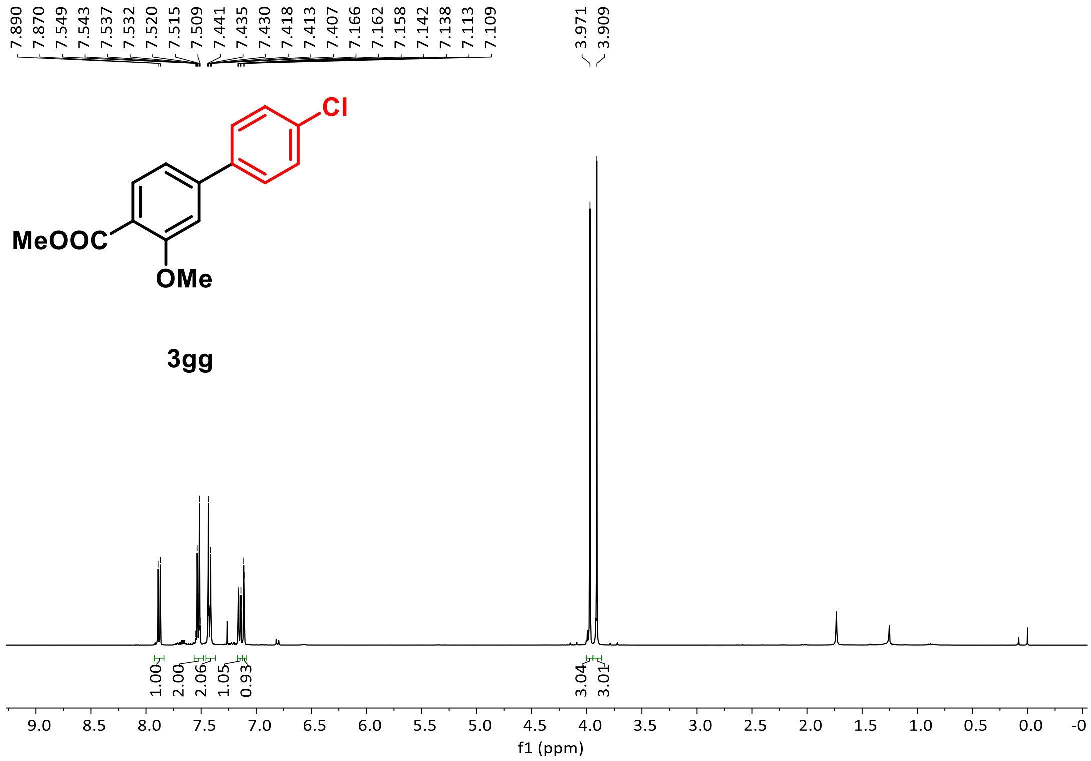

Supplementary Figure 90. ^{1}H NMR of compound **3gg** (400 MHZ, r.t., $CDCl_3$)

^{13}C NMR

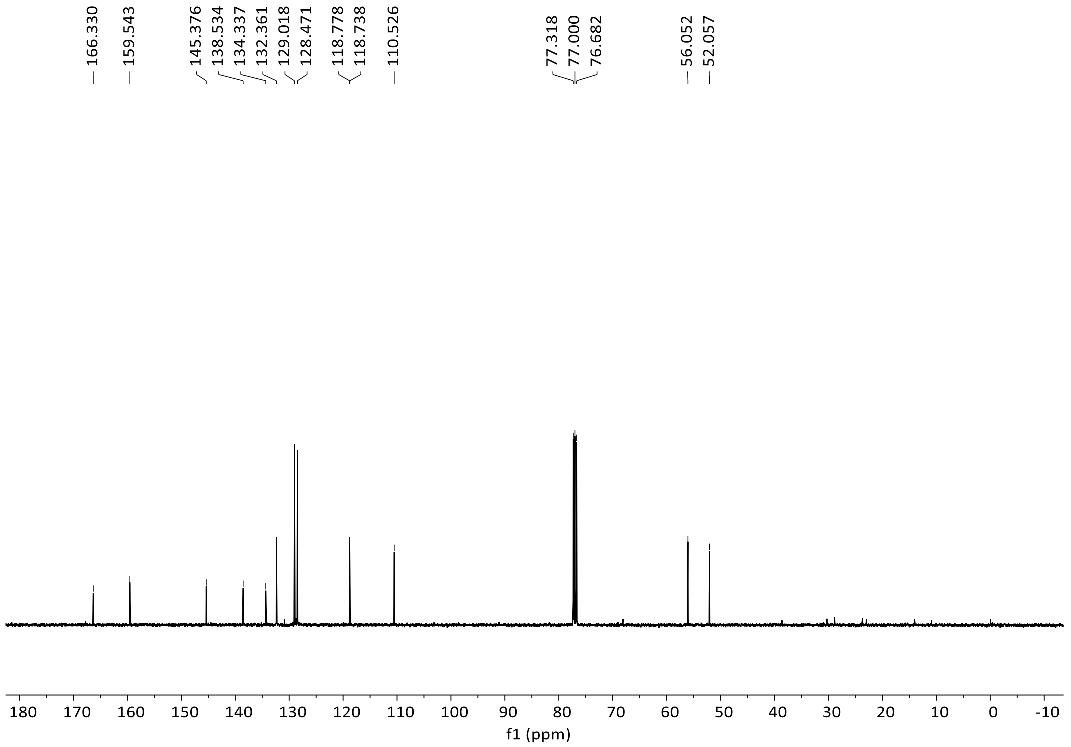

Supplementary Figure 91. ^{13}C NMR of compound **3gg** (101 MHZ, r.t., $CDCl_3$)

^{1}H NMR

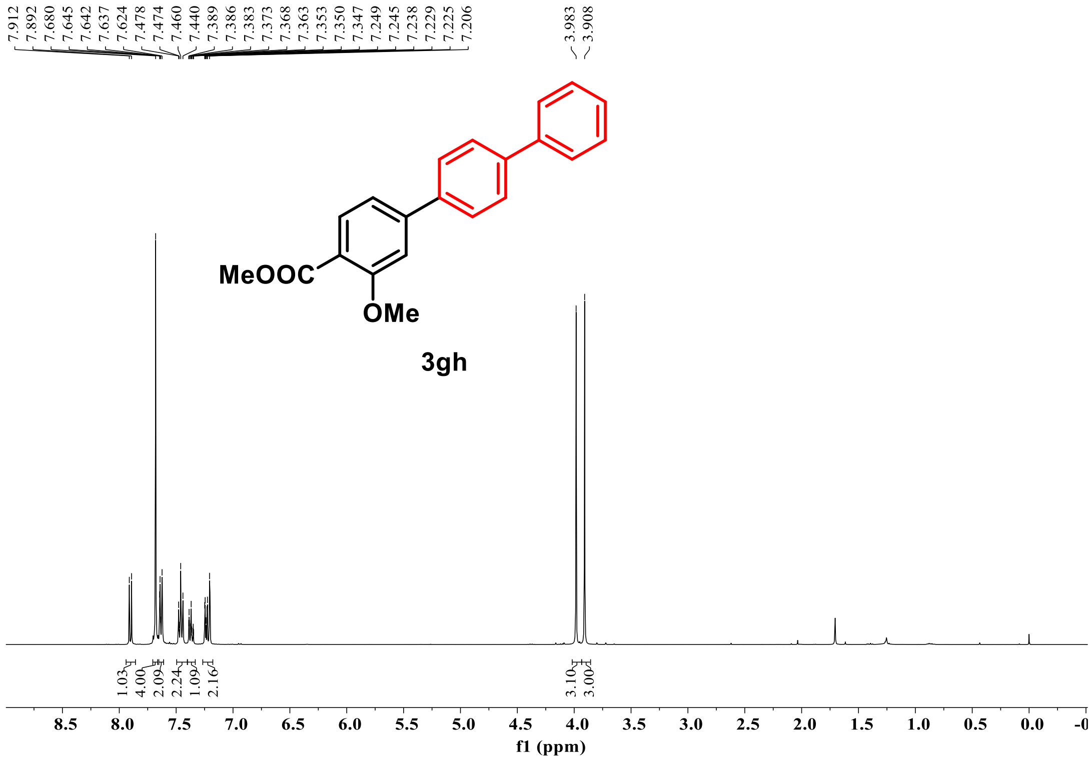

Supplementary Figure 92. ^{1}H NMR of compound **3gh** (400 MHZ, r.t., $CDCl_3$)

^{13}C NMR

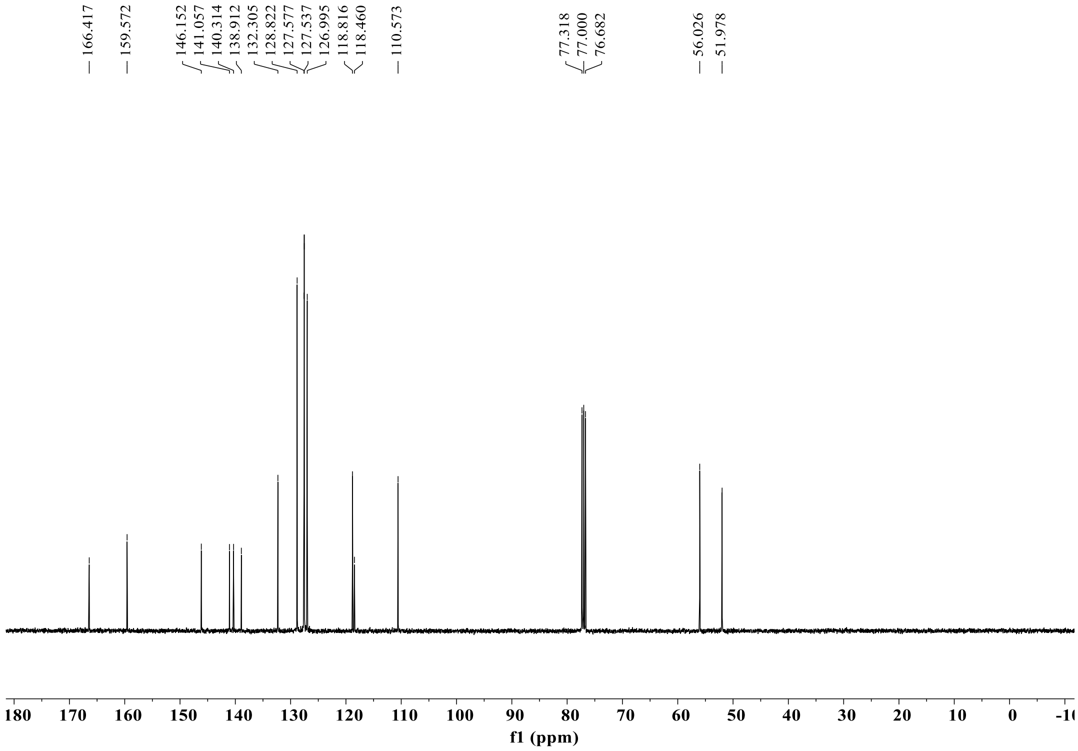

Supplementary Figure 93. ^{13}C NMR of compound **3gh** (101 MHZ, r.t., $CDCl_3$)

^{1}H NMR

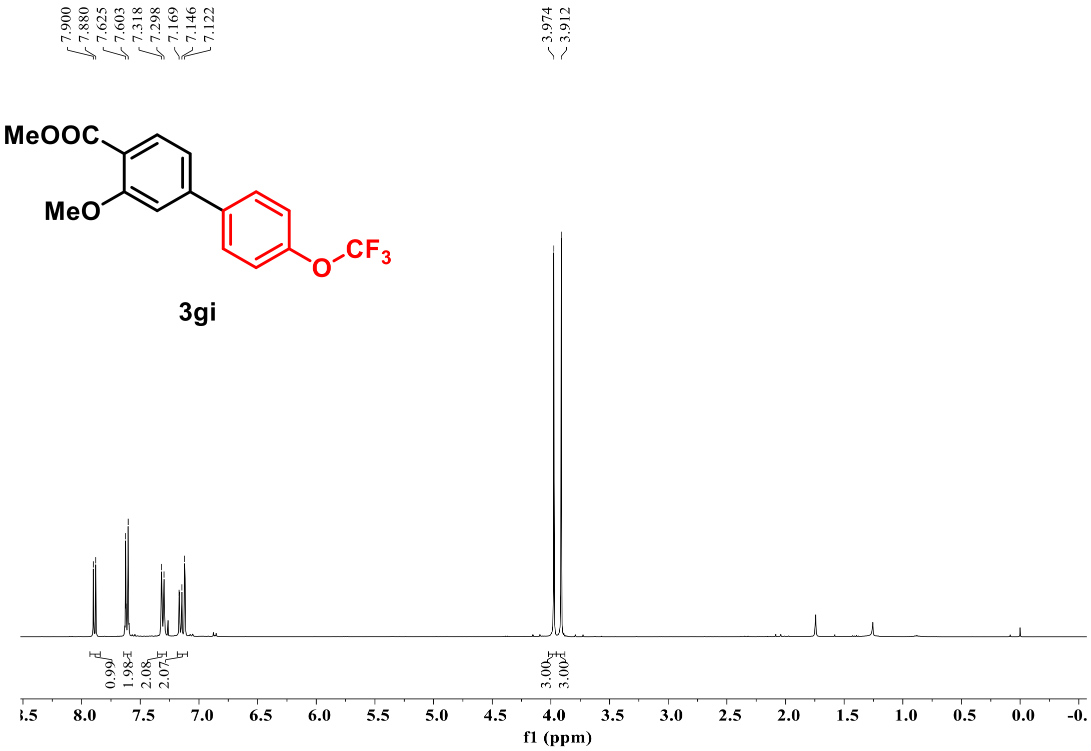

Supplementary Figure 94. ^{1}H NMR of compound **3gi** (400 MHZ, r.t., $CDCl_3$)

^{13}C NMR

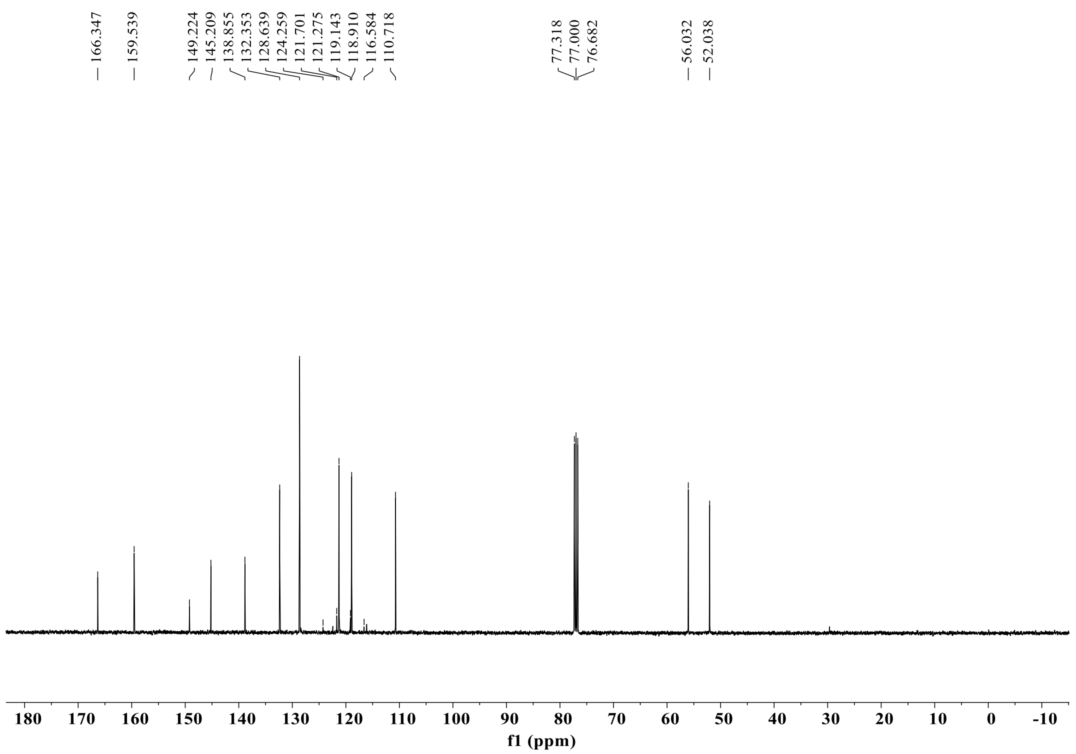

Supplementary Figure 95. ^{13}C NMR of compound **3gi** (101 MHZ, r.t., $CDCl_3$)

^{19}F NMR

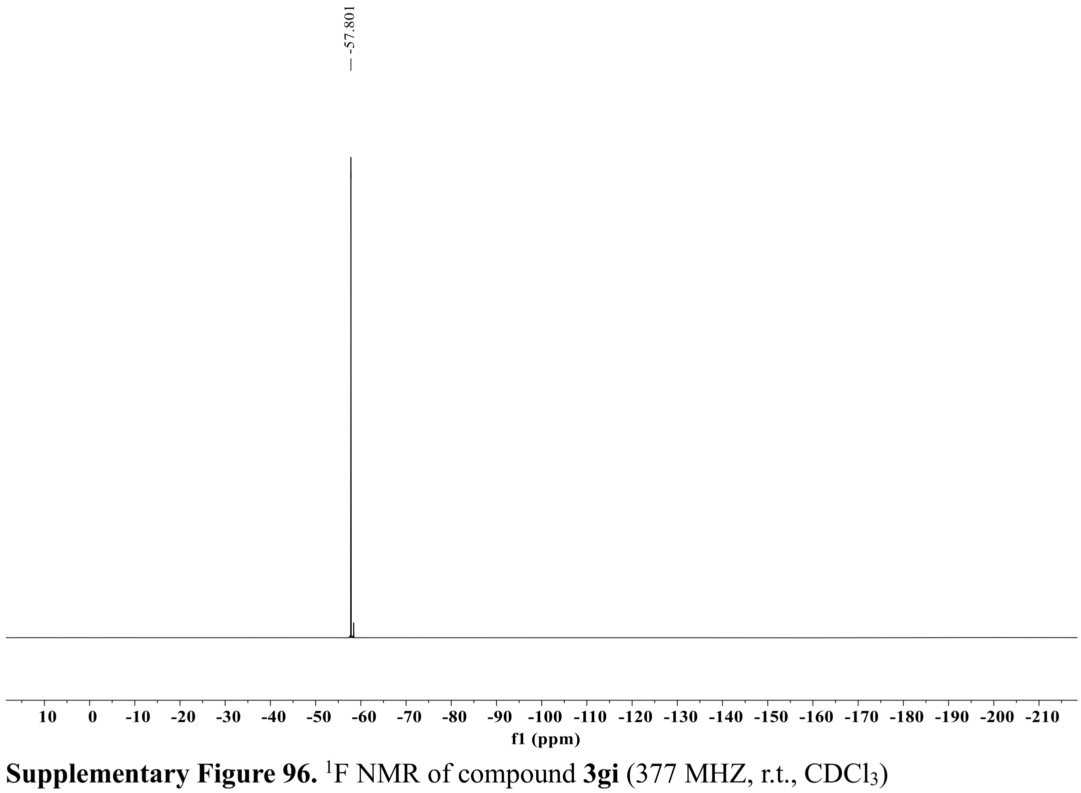

Supplementary Figure 96. ^{1}F NMR of compound **3gi** (377 MHZ, r.t., $CDCl_3$)

^{1}H NMR

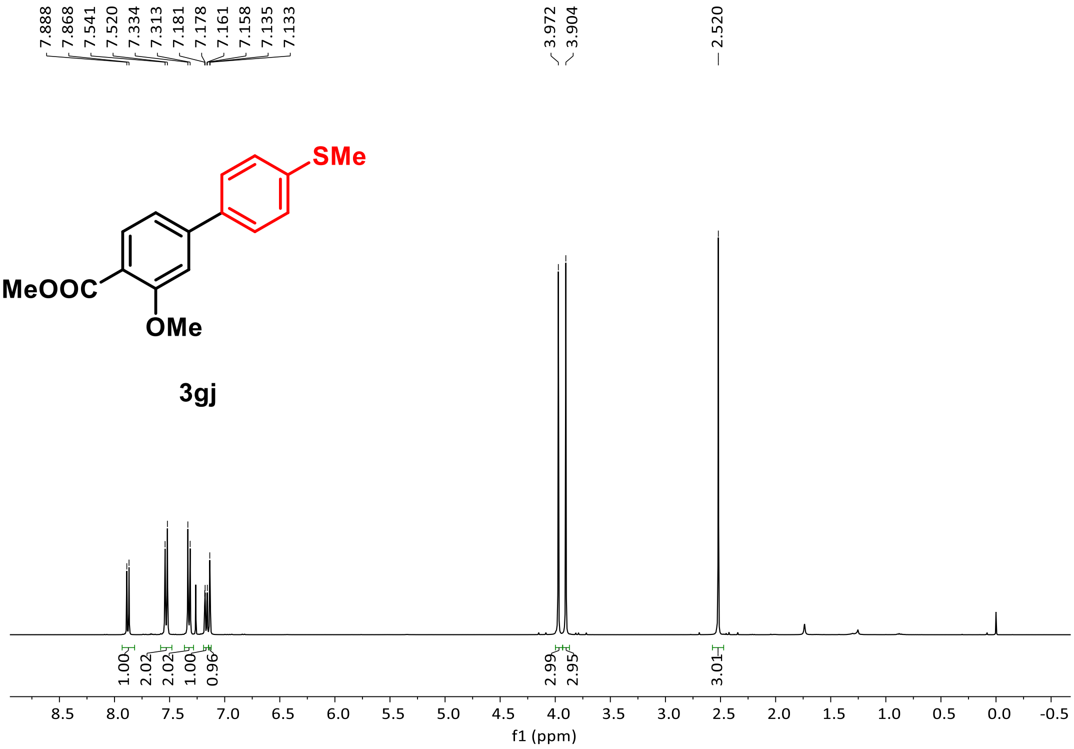

Supplementary Figure 97. ^{1}H NMR of compound **3gj** (400 MHZ, r.t., $CDCl_3$)

^{13}C NMR

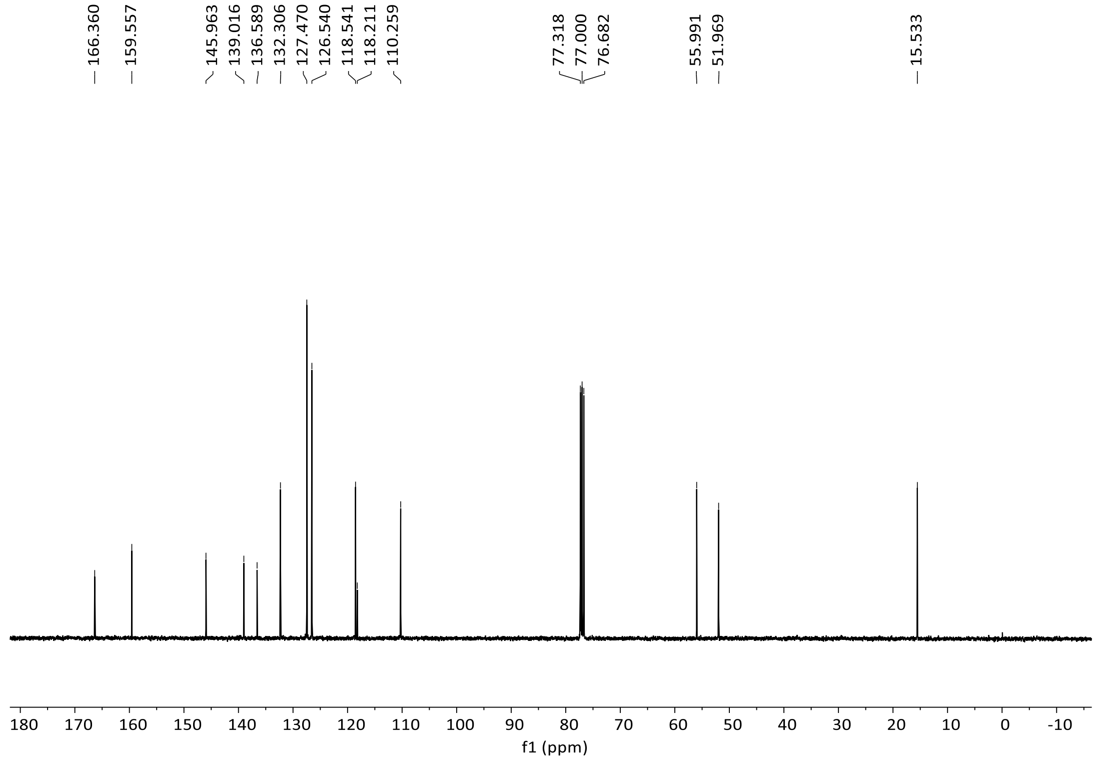

Supplementary Figure 98. ^{13}C NMR of compound **3gj** (101 MHZ, r.t., $CDCl_3$)

^{1}H NMR

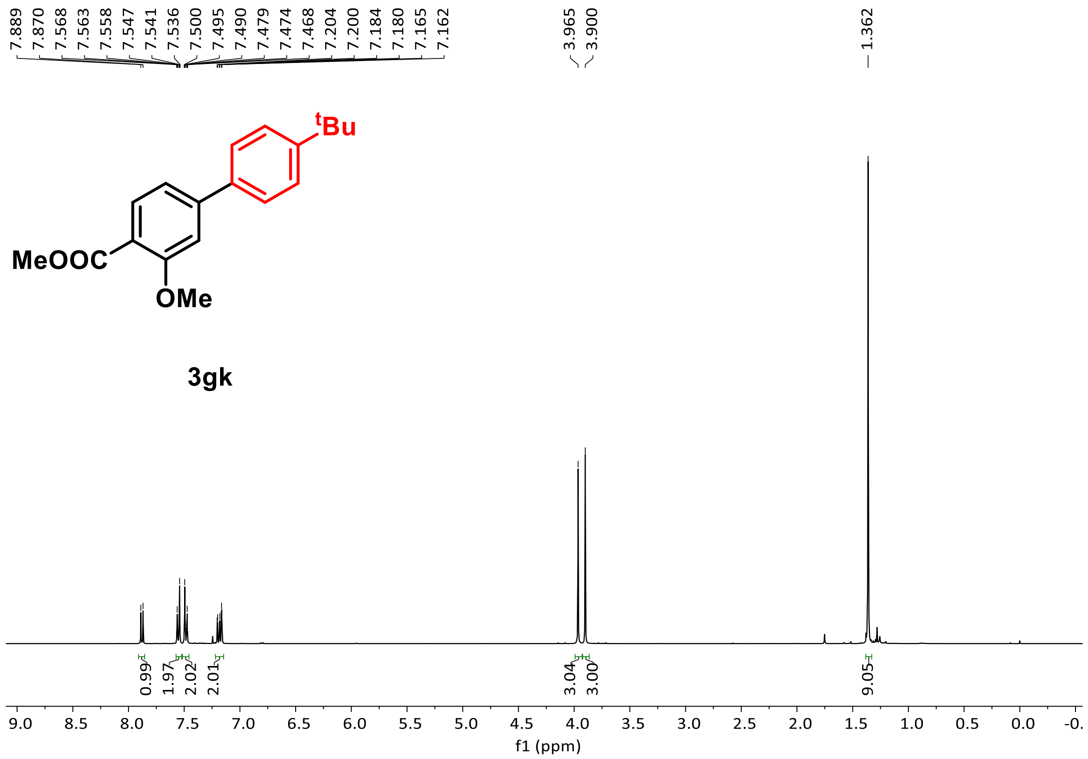

Supplementary Figure 99. ^{1}H NMR of compound **3gk** (400 MHZ, r.t., $CDCl_3$)

^{13}C NMR

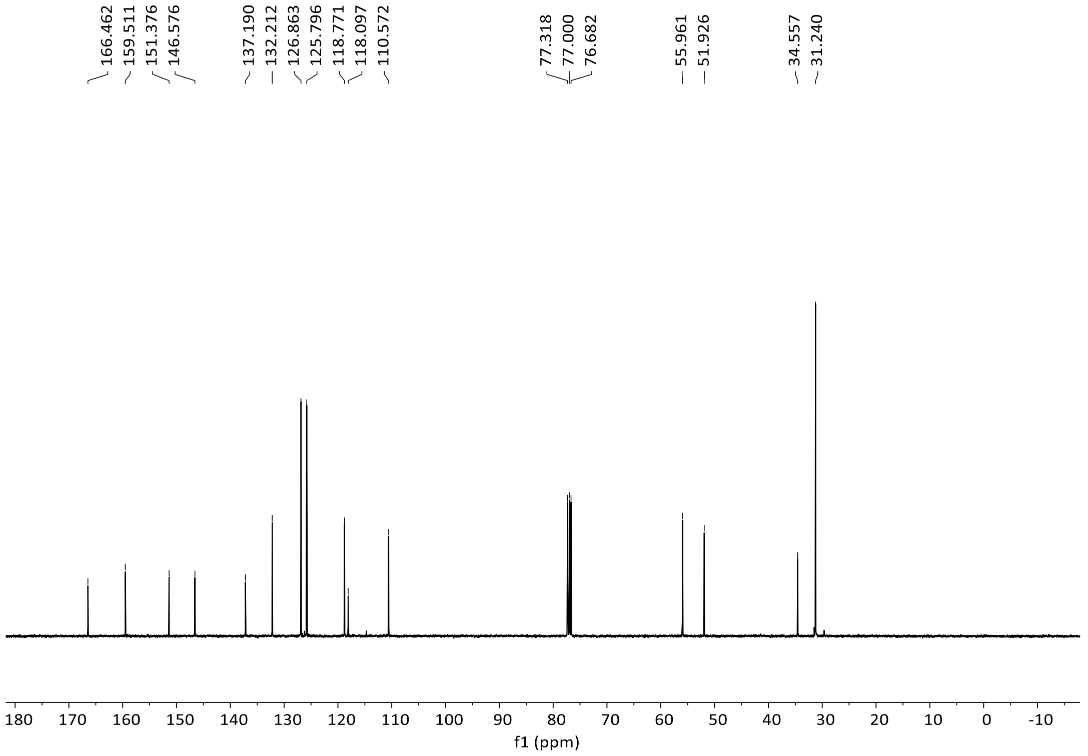

Supplementary Figure 100. ^{13}C NMR of compound **3gk** (101 MHZ, r.t., $CDCl_3$)

^{1}H NMR

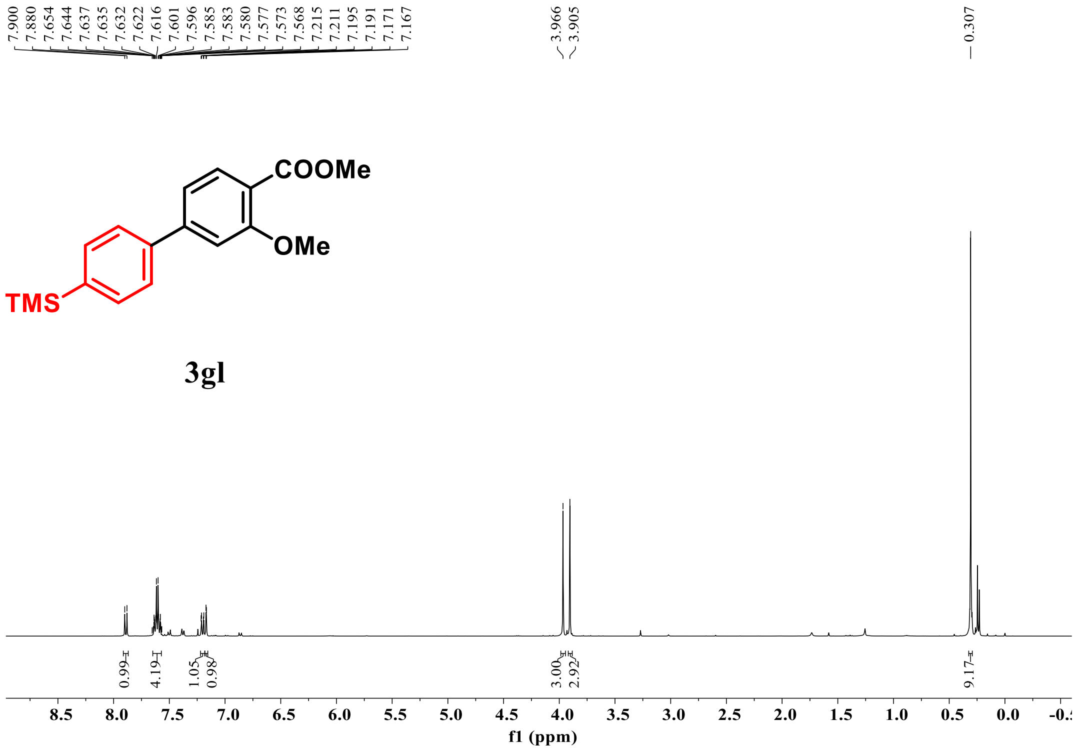

Supplementary Figure 101. ^{1}H NMR of compound **3gl** (400 MHZ, r.t., $CDCl_3$)

^{13}C NMR

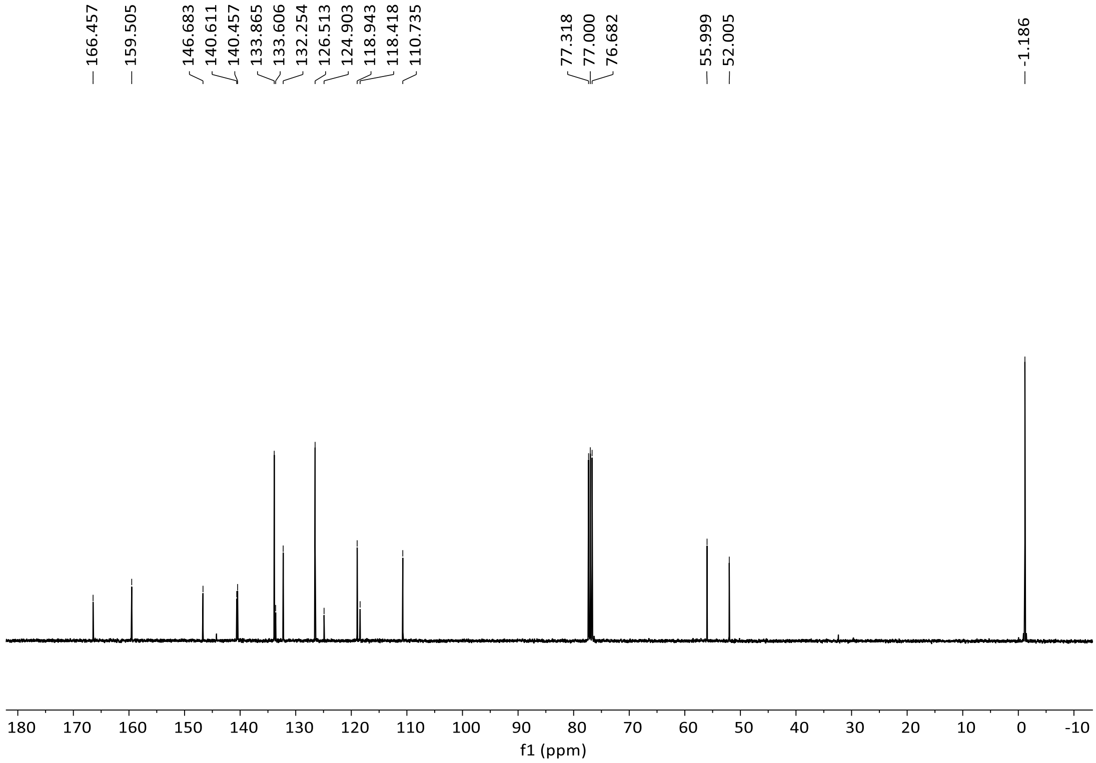

Supplementary Figure 102. ^{13}C NMR of compound **3gl** (101 MHZ, r.t., $CDCl_3$)

^{1}H NMR

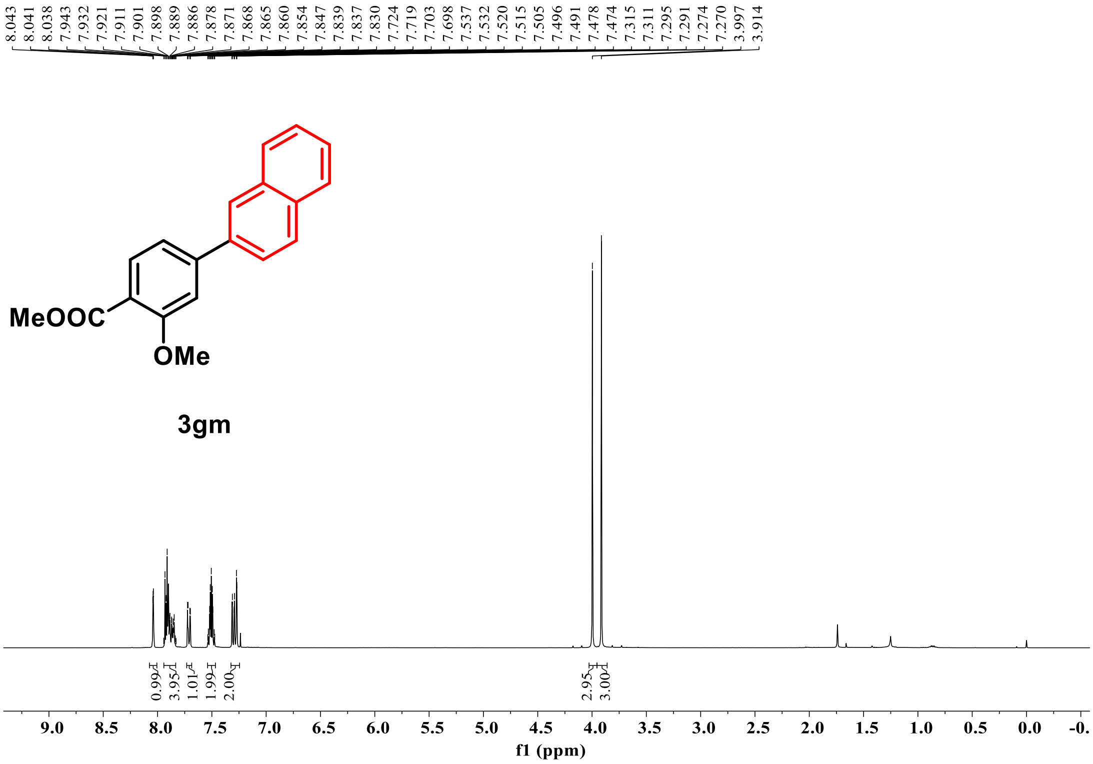

Supplementary Figure 103. ^{1}H NMR of compound **3gm** (400 MHZ, r.t., $CDCl_3$)

^{13}C NMR

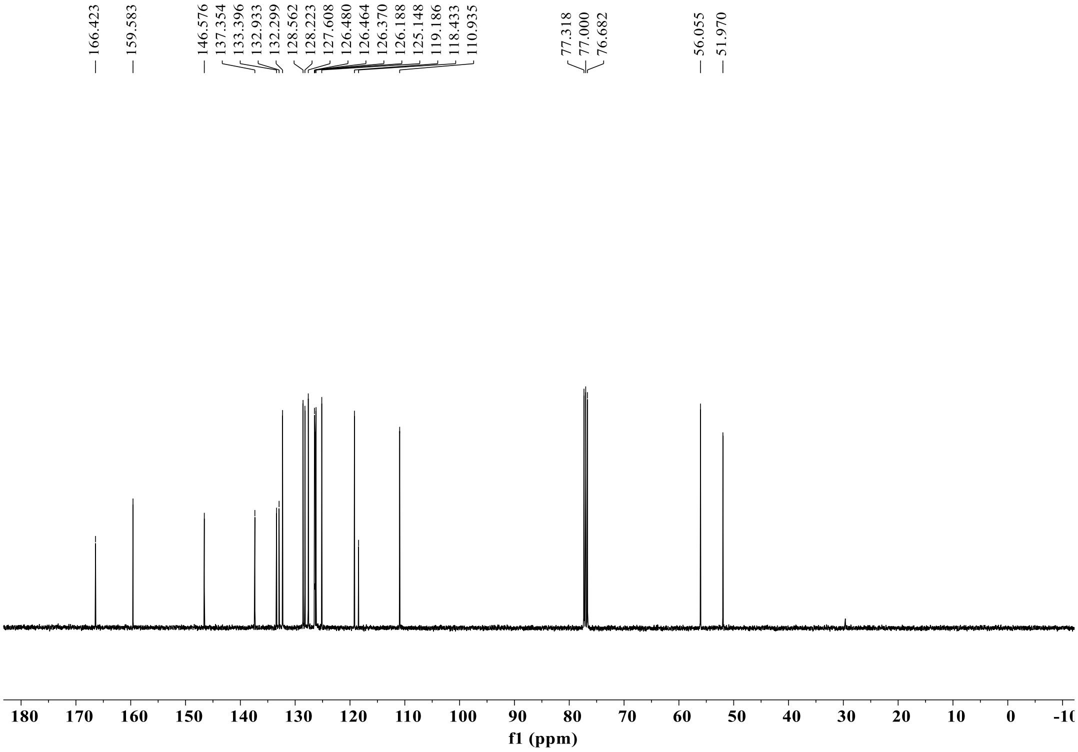

Supplementary Figure 104. ^{13}C NMR of compound **3gm** (101 MHZ, r.t., $CDCl_3$)

^{1}H NMR

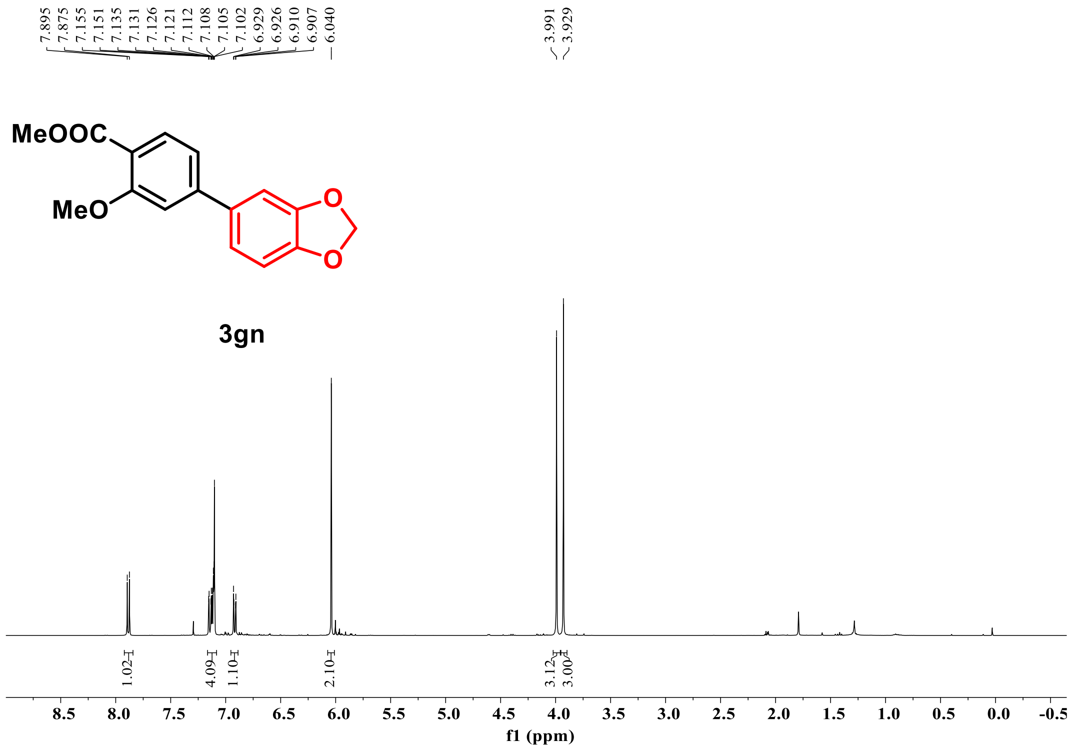

Supplementary Figure 105. ^{1}H NMR of compound **3gn** (400 MHZ, r.t., $CDCl_3$)

^{13}C NMR

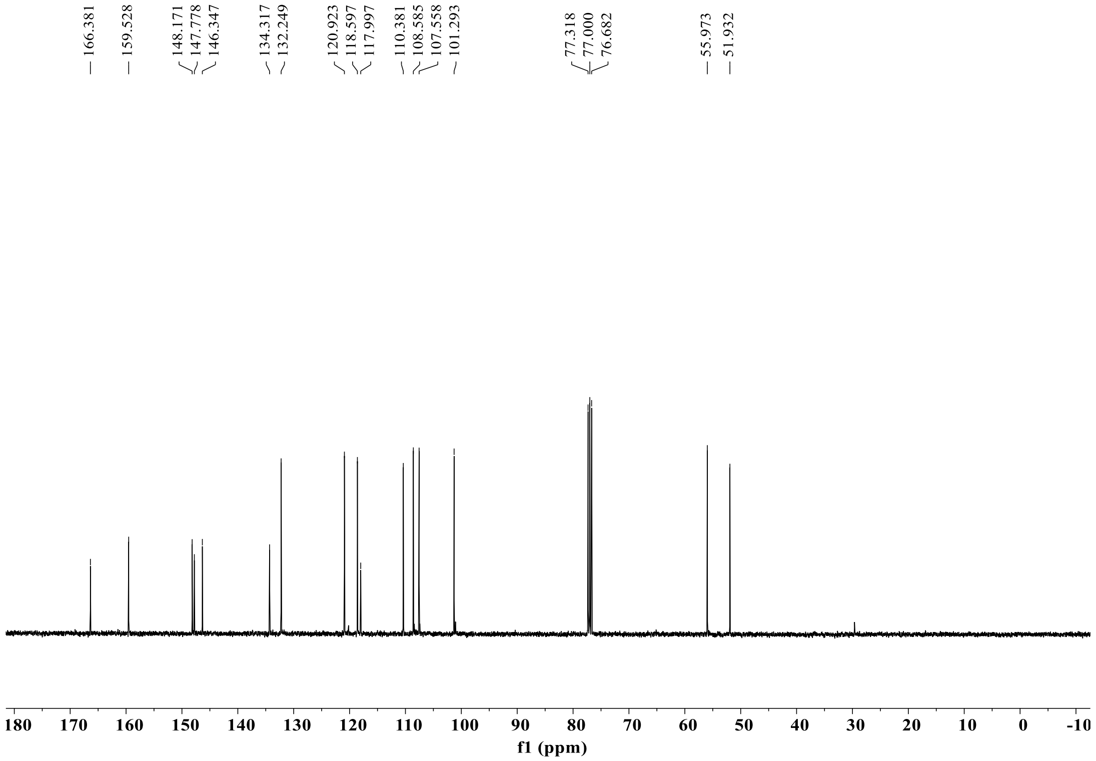

Supplementary Figure 106. ^{13}C NMR of compound **3gn** (101 MHZ, r.t., $CDCl_3$)

^{1}H NMR

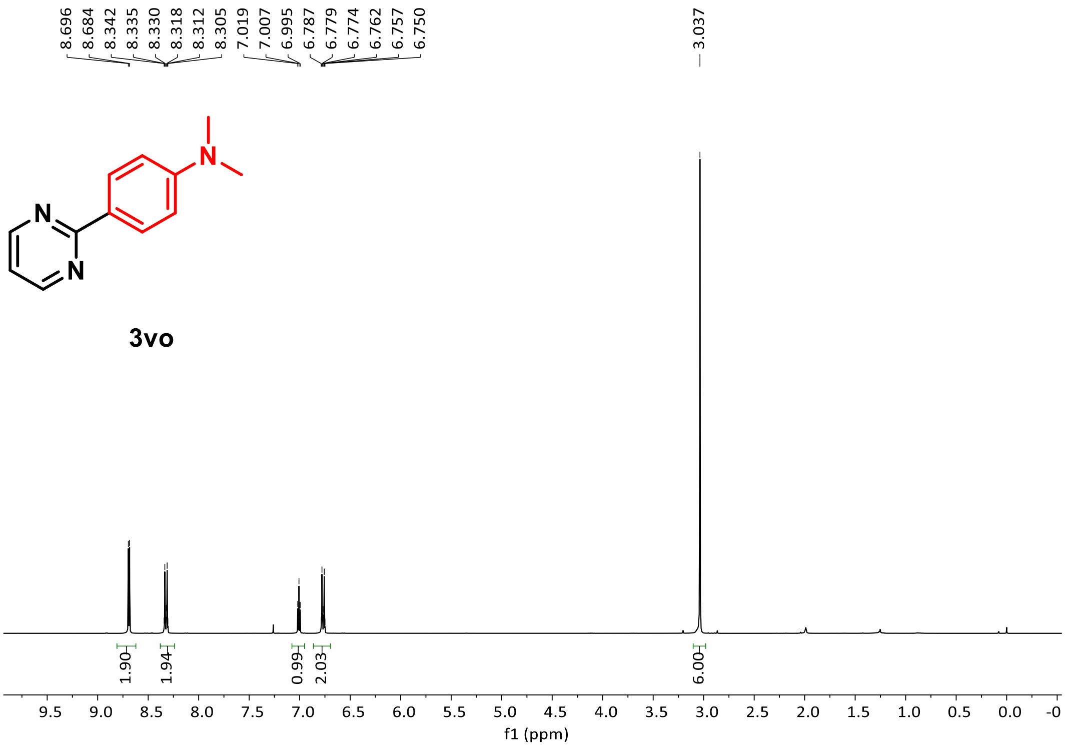

Supplementary Figure 107. ^{1}H NMR of compound **3vo** (400 MHZ, r.t., $CDCl_3$)

^{13}C NMR

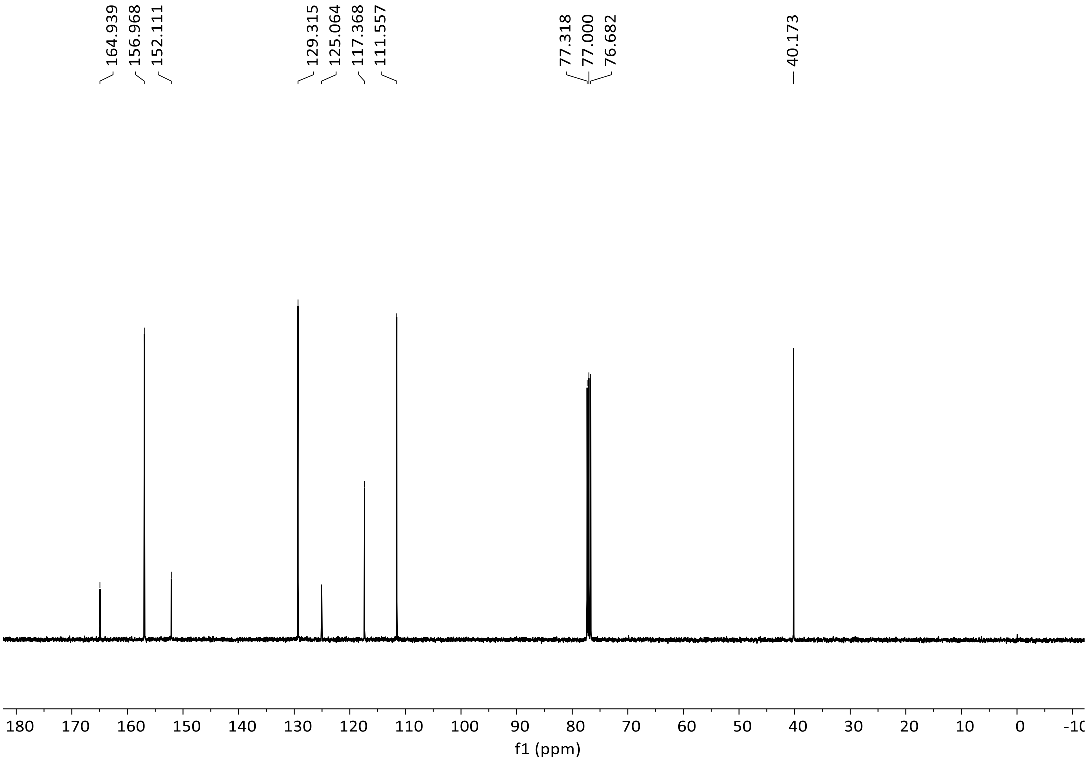

Supplementary Figure 108. ^{13}C NMR of compound **3vo** (101 MHZ, r.t., $CDCl_3$)

^{1}H NMR

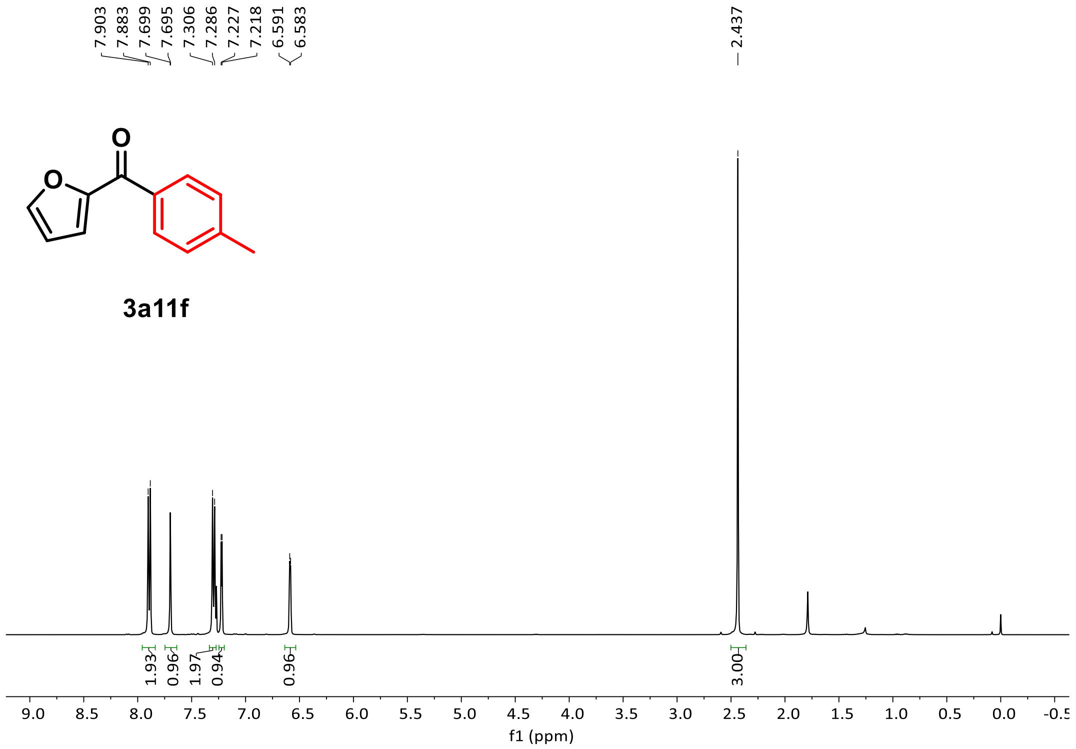

Supplementary Figure 109. ^{1}H NMR of compound **3a11f** (400 MHZ, r.t., $CDCl_3$)

^{13}C NMR

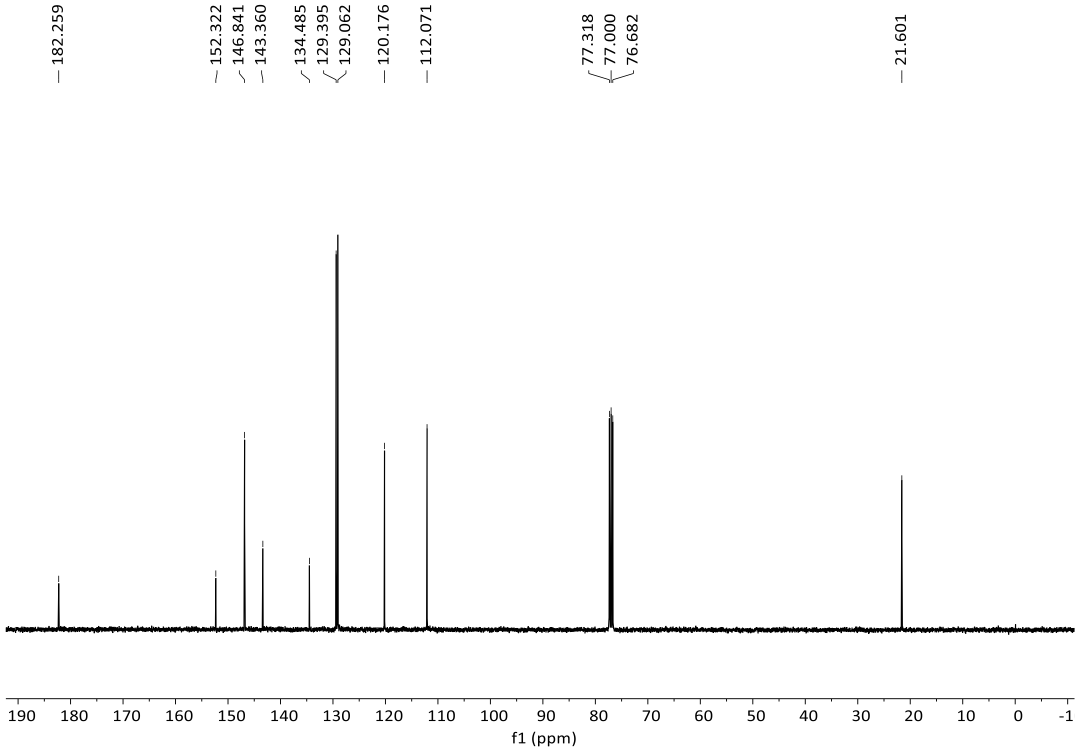

Supplementary Figure 110. ^{13}C NMR of compound **3a11f** (101 MHZ, r.t., $CDCl_3$)

^{1}H NMR

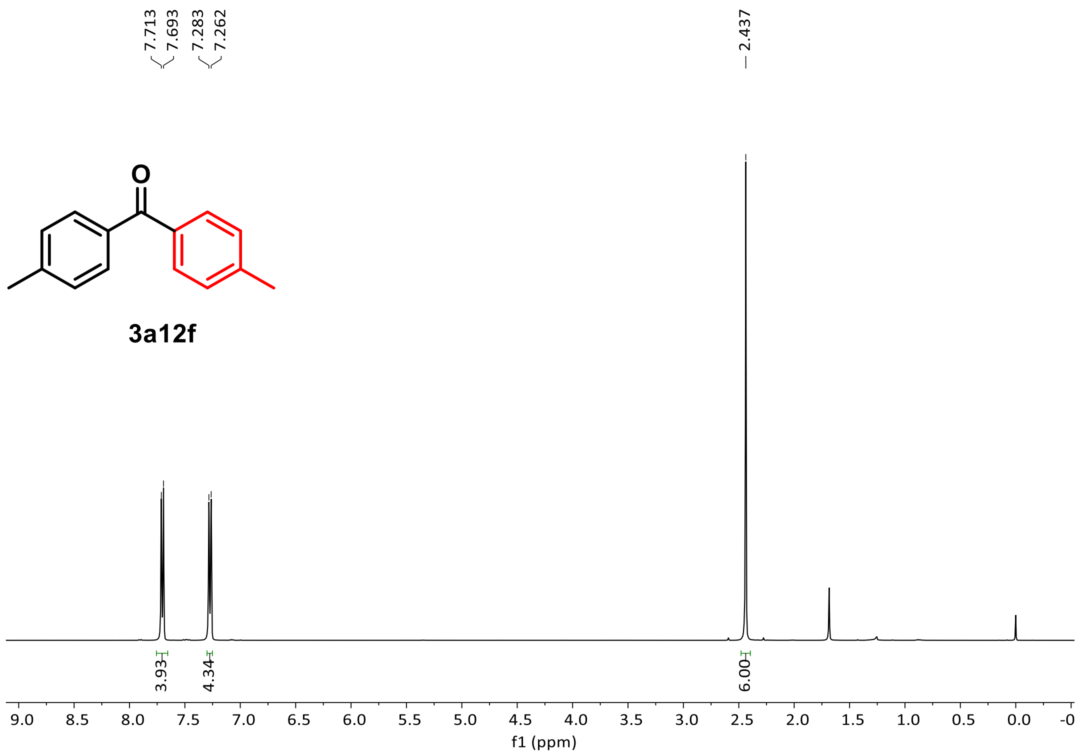

Supplementary Figure 111. ^{1}H NMR of compound **3a12f** (400 MHZ, r.t., $CDCl_3$)

^{13}C NMR

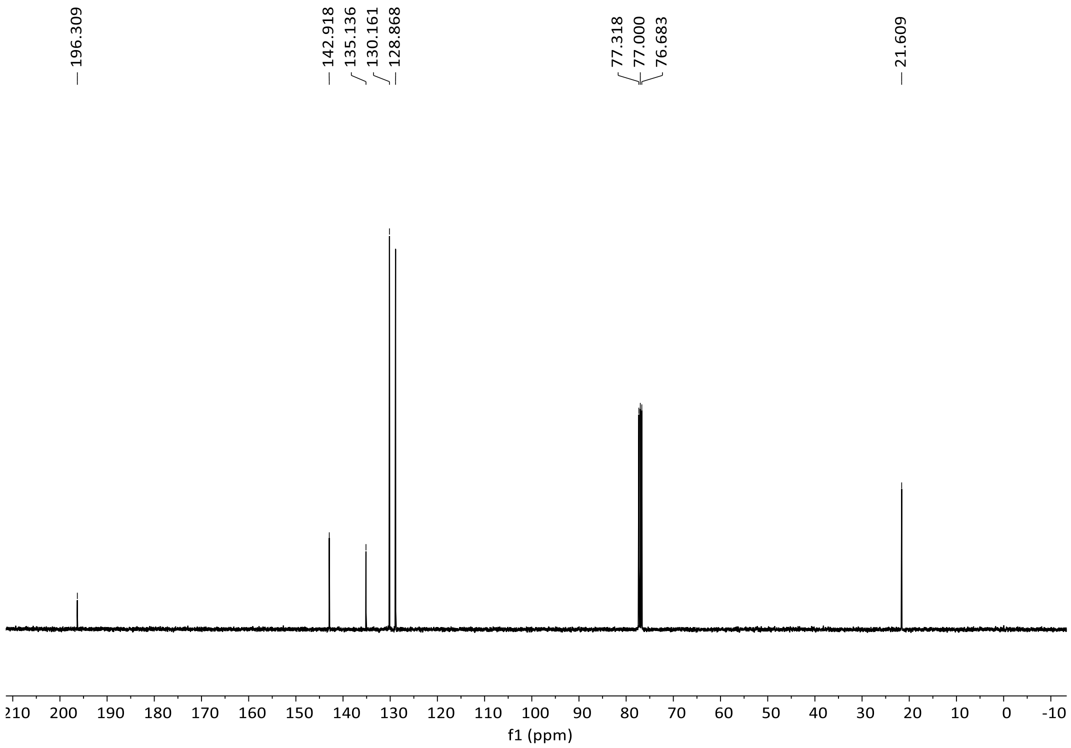

Supplementary Figure 112. ^{13}C NMR of compound **3a12f** (101 MHZ, r.t., $CDCl_3$)

^{1}H NMR

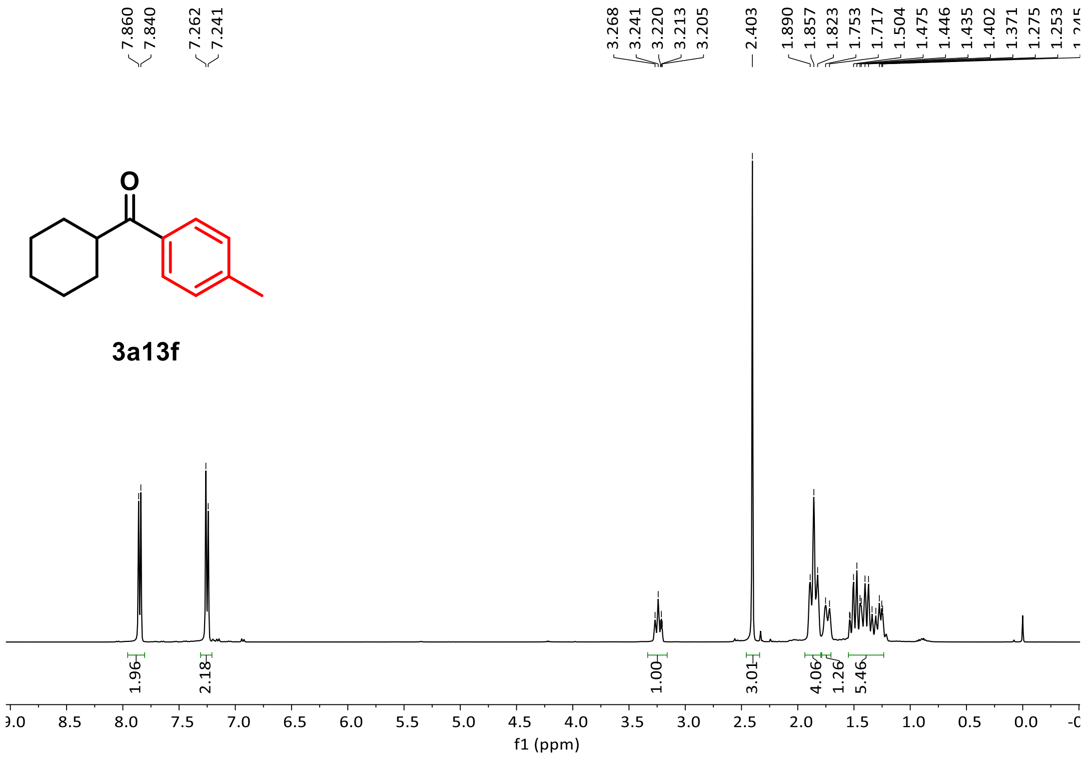

Supplementary Figure 113. ^{1}H NMR of compound **3a13f** (400 MHZ, r.t., $CDCl_3$)

^{13}C NMR

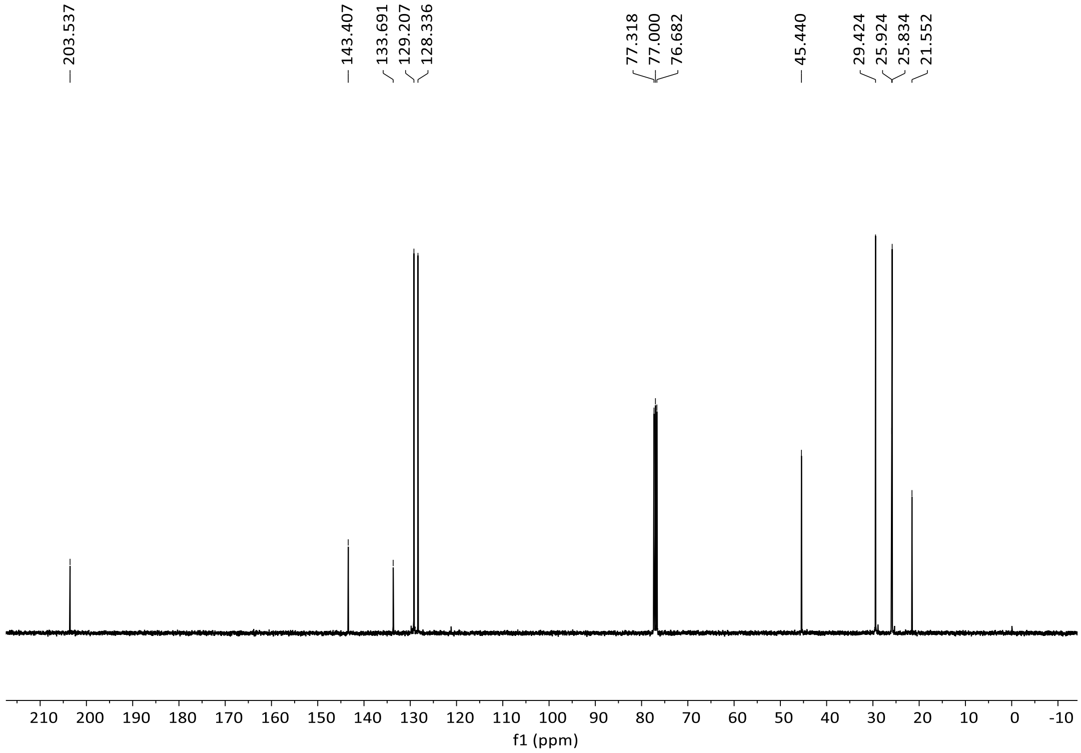

Supplementary Figure 114. ^{13}C NMR of compound **3a13f** (101 MHZ, r.t., $CDCl_3$)

^{1}H NMR

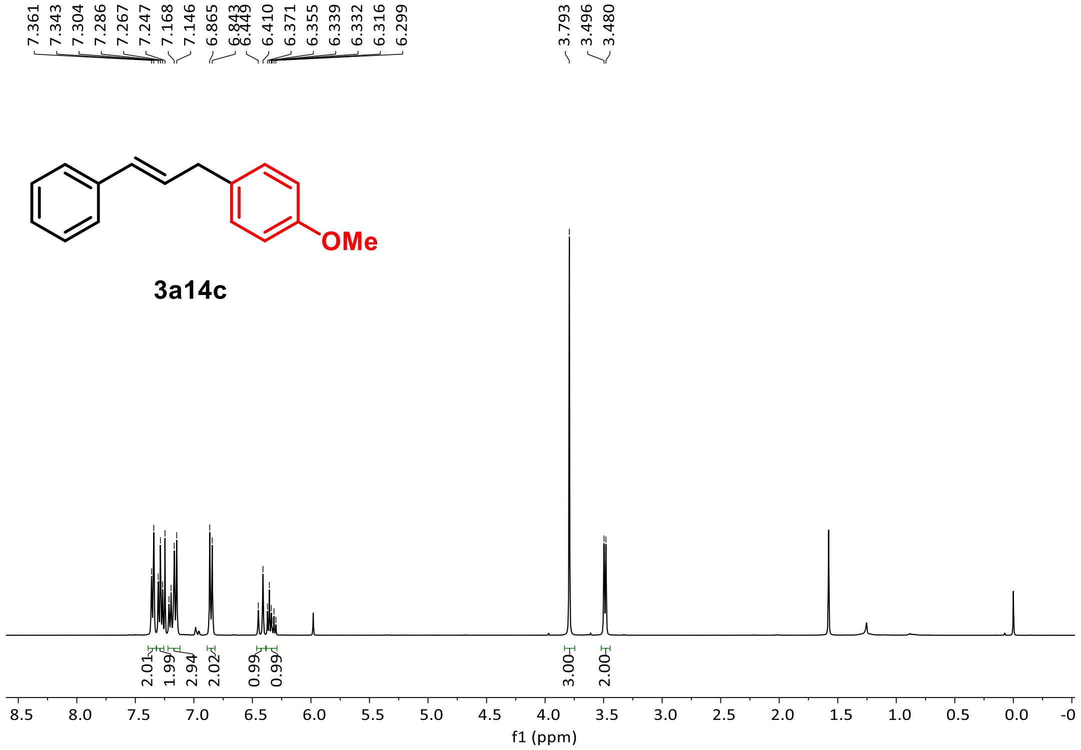

Supplementary Figure 115. ¹H NMR of compound **3a14c** (400 MHZ, r.t., $CDCl_3$)

^{13}C NMR

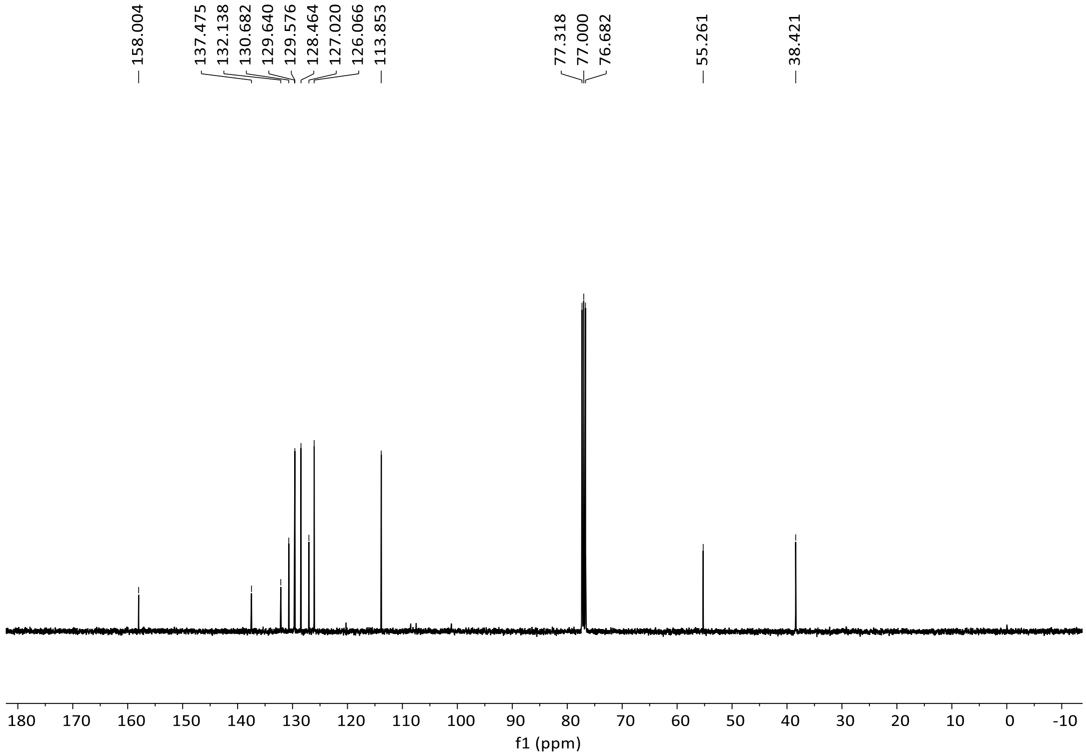

Supplementary Figure 116. ^{13}C NMR of compound **3a14c** (101 MHZ, r.t., $CDCl_3$)

^{1}H NMR

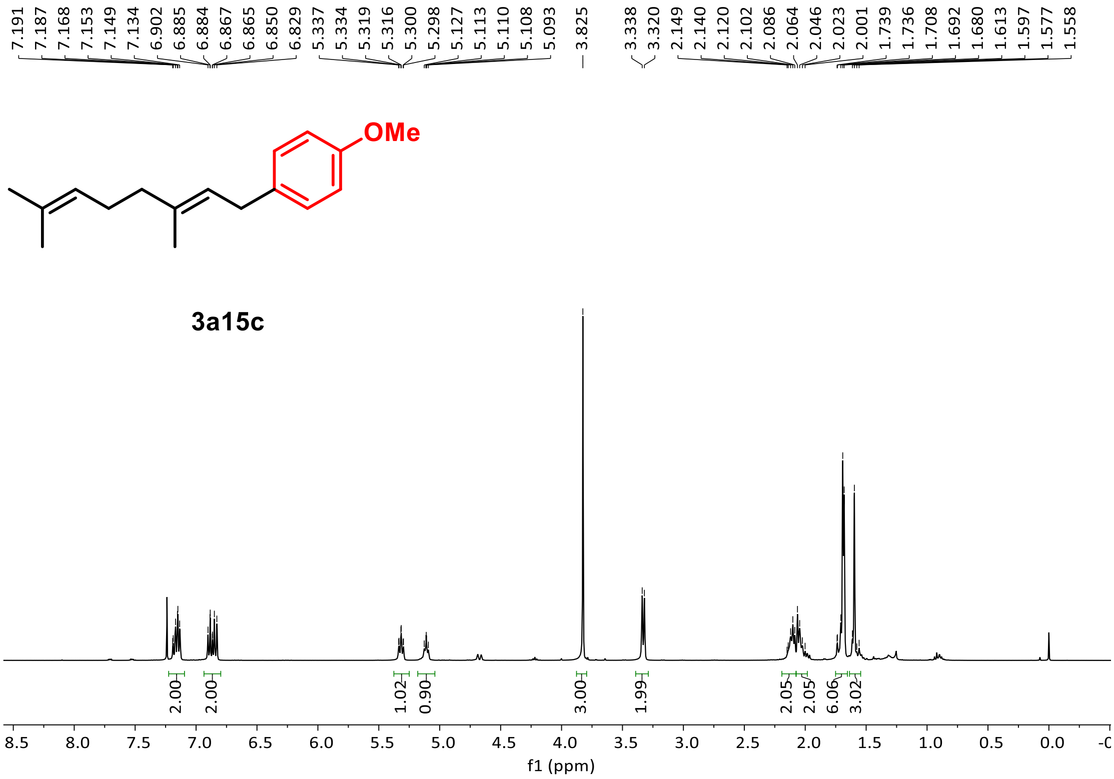

Supplementary Figure 117. ^{1}H NMR of compound **3a15c** (400 MHZ, r.t., $CDCl_3$)

^{13}C NMR

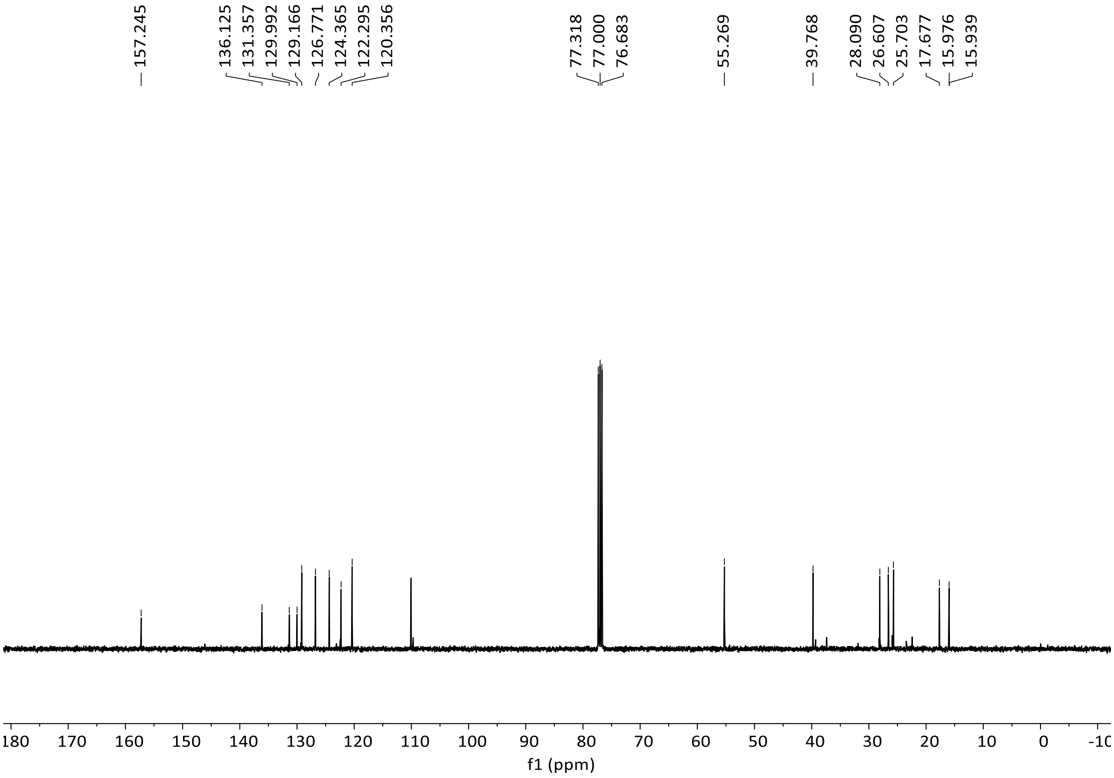

Supplementary Figure 118. ^{13}C NMR of compound **3a15c** (101 MHZ, r.t., $CDCl_3$)

^{1}H NMR

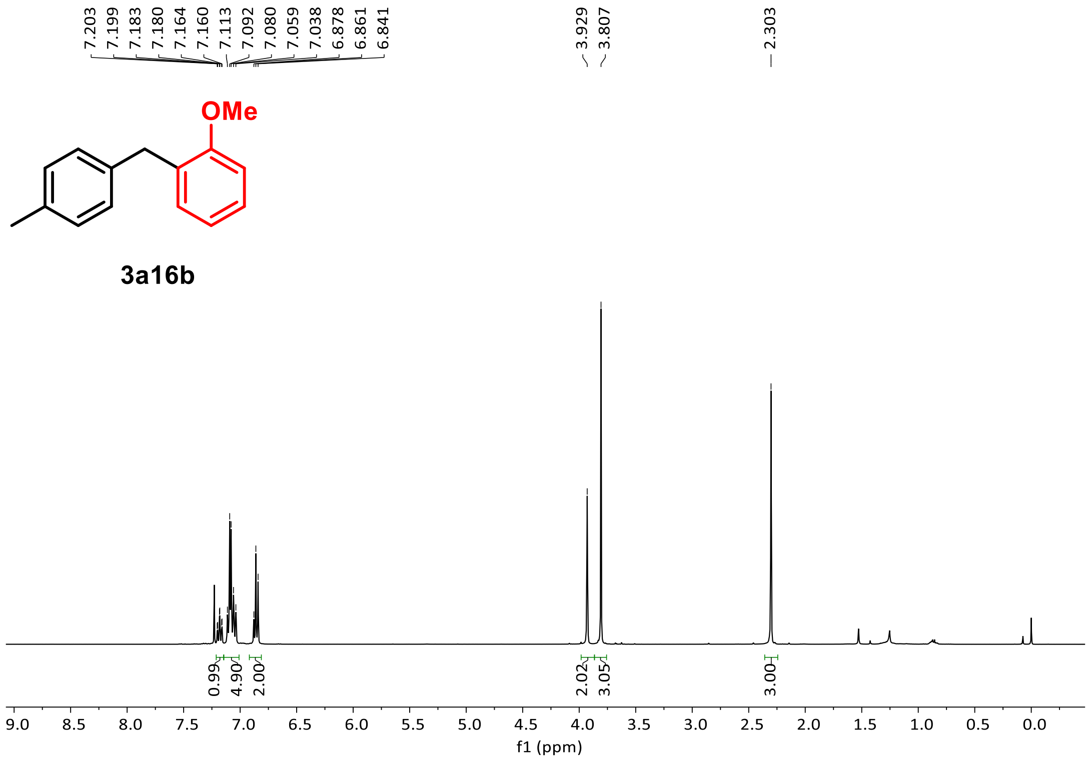

Supplementary Figure 119. ^{1}H NMR of compound **3a16b** (400 MHZ, r.t., $CDCl_3$)

^{13}C NMR

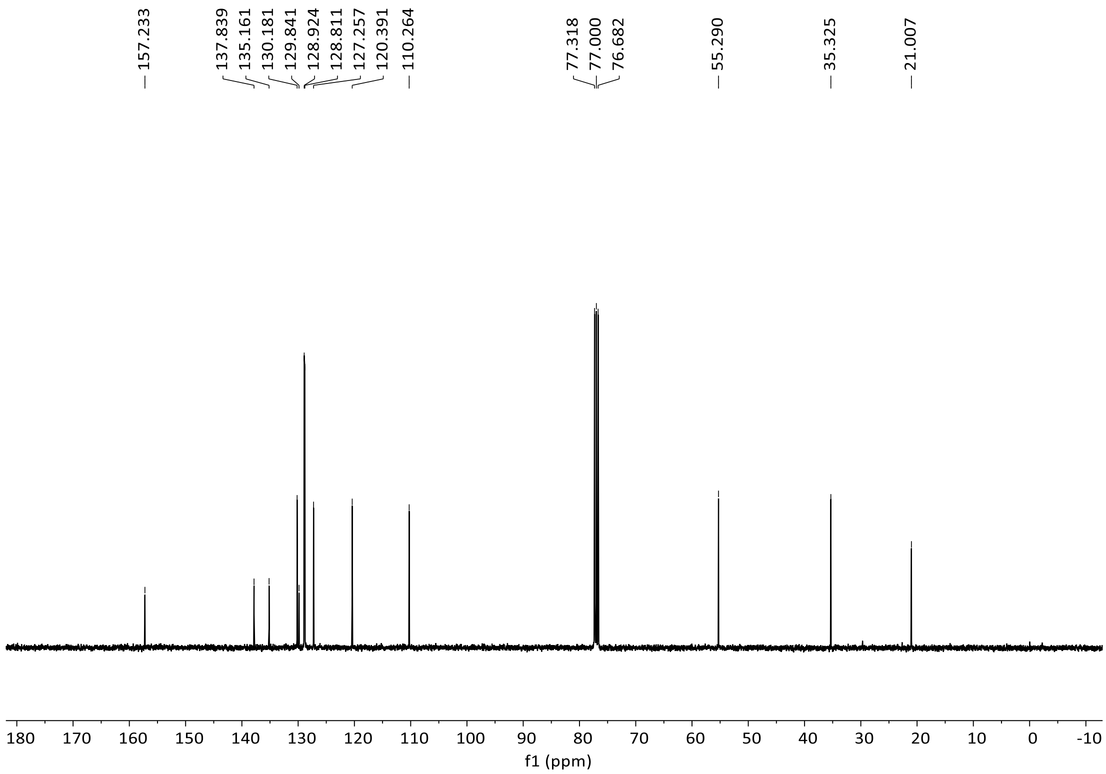

Supplementary Figure 120. ^{13}C NMR of compound **3a16b** (101 MHZ, r.t., $CDCl_3$)

4 Supplementary References

1. Krasovskiy, A.; Knochel, P. Convenient Titration Method for Organometallic Zinc, Magnesium,

and Lanthanide Reagents. *Synthesis* **2006**, *5*, 0890-0891.
2. Neese, F. ORCA: An ab initio, DFT and semiempirical SCF-MO package; Version 3.0.1 (University of Bonn, Germany, 2013).
3. Schaefer, A.; Horn H.; Ahlrichs, R. Fully optimized contracted Gaussian basis sets for atoms Li to Kr. *J. Chem. Phys.* **1992,** *97*, 2571–2577.
4. DeBeer George, S.; Petrenko, T.; Neese, F. Prediction of Iron K-Edge Absorption Spectra Using Time-Dependent Density Functional Theory. *J. Phys. Chem.* **2008,** *112*, 12936–12943.
5. Frisch, M. J.; Trucks, G. W.; Schlegel, H. B.; Scuseria, G. E.; Robb, M. A.; Cheeseman, J. R.; Scalmani, G.; Barone, V.; Mennucci, B.; Petersson, G. A.; Nakatsuji, H.; Caricato, M.; Li, X.; Hratchian, H. P.; Izmaylov, A. F.; Bloino, J.; Zheng, G.; Sonnenberg, J. L.; Hada, M.; Ehara, M.; Toyota, K.; Fukuda, R.; Hasegawa, J.; Ishida, M.; Nakajima, T.; Honda, Y.; Kitao, O.; Nakai, H.; Vreven, T.; Montgomery, Jr., J. A.; Peralta, J. E.; Ogliaro, F.; Bearpark, M.; Heyd, J. J.; Brothers, E.; Kudin, K. N.; Staroverov, V. N.; Keith, T.; Kobayashi, R.; Normand, J.; Raghavachari, K.; Rendell, A.; Burant, J. C.; Iyengar, S. S.; Tomasi, J.; Cossi, M.; Rega, N.; Millam, J. M.; Klene, M.; Knox, J. E.; Cross, J. B.; Bakken, V.; Adamo, C.; Jaramillo, J.; Gomperts, R.; Stratmann, R. E.; Yazyev, O.; Austin, A. J.; Cammi, R.; Pomelli, C.; Ochterski, J. W.; Martin, R. L.; Morokuma, K.; Zakrzewski, V. G.; Voth, G. A.; Salvador, P.; Dannenberg, J. J.; Dapprich, S.; Daniels, A. D.; Farkas, O.; Foresman, J. B.; Ortiz, J. V.; Cioslowski, J.; and Fox, D. J. Gaussian 09, revision D.01; Gaussian, Inc.: Wallingford, CT, **2013**.
6. Becke, A. D. Density-functional thermochemistry. III. The role of exact exchange. *J. Chem. Phys.* **1993**, *98*, 5648−5652.
7. Lee, C.; Yang, W.; Parr, R. G. Development of the Colle-Salvetti correlation-energy formula into a functional of the electron density. *Phys. Rev. B*. **1988**, *37*, 785−789.
8. Hellweg, A.; Hättig, C.; Höfener, S.; Klopper, W. Optimized accurate auxiliary basis sets for RI-MP2 and RI-CC2 calculations for the atoms Rb to Rn. *Theoritical Chemistry Accounts*. **2007**, *117*, 587–597.
9. Zhao, Y.; Truhlar, D. G. The M06 suite of density functionals for main group thermochemistry, thermochemical kinetics, noncovalent interactions, excited states, and transition elements: two new functionals and systematic testing of four M06-class functionals and 12 other functionals. *Theor. Chem. Acc.* **2008**, *120*, 215−241.
10. Marenich, A. V.; Cramer, C. J.; Truhlar, D. G. Universal Solvation Model Based on Solute Electron Density and on a Continuum Model of the Solvent Defined by the Bulk Dielectric Constant and Atomic Surface Tensions. *J. Phys. Chem. B.* **2009**, *113*, 6378−6396.
11. CYLview, 1.0b; C. Y. Legault, Université de Sherbrooke, **2009**.
12. Khan, R. I.; Pitchumani, K. A. pyridinium modified β-cyclodextrin: an ionic supramolecular ligand for palladium acetate in C–C coupling reactions in water. *Green Chem*. **2016**, *18*, 5518.
13. Bolliger, J. L.; Frech, C. M. The 1,3-Diaminobenzene-Derived Aminophosphine Palladium Pincer Complex {C_6H_3[NHP(piperidinyl)$_2$]$_2$Pd(Cl)} – A Highly Active Suzuki–Miyaura Catalyst with Excellent Functional Group Tolerance. *Adv. Synth. Catal.* **2010**, *352*, 1075–1080.
14. Manolikakes, G.; Knochel, P. Radical Catalysis of Kumada Cross-Coupling Reactions Using Functionalized Grignard Reagents. *Angew. Chem. Int. Ed*. **2009**, *48*, 205 –209.
15. Jia, R.; Wang, J.; Jiang, Y.; Ni, B.; Niu, T. Photocatalyzed oxidative cleavage of C≡C bond to carbonyl compounds by a recyclable homogeneous carbon nitride semiconductor/aqueous system. *Org. Biomol. Chem.* **2022**, *20*, 8305–8312.

16. Wu, D.; Wang, Z. P,N,N-Pincer nickel-catalyzed cross-coupling of aryl fluorides and chlorides. *Org. Biomol. Chem*. **2014**, 12, 6414.
17. Diebold, C.; Becht, J.; Lu, J.; Toy P. H.; Le Drian, C. An Efficient and Reusable Palladium Catalyst Supported on a Rasta Resin for Suzuki–Miyaura Cross-Couplings. *Eur. J. Org. Chem*. **2012**, 893–896.
18. Vila, C.; Cembellin, S.; Hornillos, V.; Giannerini, M.; Fananas-Mastral, M.Feringa, B. L. ^{t}BuLi-Mediated One-Pot Direct Highly Selective Cross-Coupling of Two Distinct Aryl Bromides. *Chemistry* **2015**, *21*, 15520-15524.
19. Wu, T. F.; Zhang, Y. J.; Fu, Y.; Liu, F. J.; Tang, J. T.; Liu, P.; Toste, F. D.Ye, B. Zirconium-Redox-Shuttled Cross-Electrophile Coupling of Aromatic and Heteroaromatic Halides. *Chem* **2021**, *7*, 1963-1974.
20. Chen, X. Y.; Nie, X. X.; Wu, Y.; Wang, P. para-Selective arylation and alkenylation of monosubstituted arenes using thianthrene S-oxide as a transient mediator. *Chem. Commun*. **2020**, *56*, 5058-5061.
21. Martinez, E. E.; Larson, A. J. S.; Fuller, S. K.; Petersen, K. M.; Smith, S. J.; Michaelis, D. J. 2-Phosphinoimidazole Ligands: N–H NHC or P–N Coordination Complexes in Palladium-Catalyzed Suzuki–Miyaura Reactions of Aryl Chlorides. *Organometallics* **2021**, *40*, 1560-1564.
22, Luo, W.; Mu, Q.; Qiu, W.; Liu, T.; Yang, F.; Liu, X.; Tang, J. A novel Friedlander-type synthesis of 3-aryl quinolines from 3-oxo-2,3-diaryl-propionaldehydes. *Tetrahedron* **2011**, *67*, 7090-7095.
23. Rossi, S.; Herbrik, F.; Resta, S.; Puglisi, A. Supported Eosin Y as a Photocatalyst for C-H Arylation of Furan in Batch and Flow. *Molecules* **2022**, *27*, 5096.
24. Peng, H.; Chen, Y. Q.; Mao, S. L.; Pi, Y. X.; Chen, Y.; Lian, Z. Y.; Meng, T.; Liu, S. H.; Yu, G. A. A general catalyst for Suzuki-Miyaura and Sonogashira reactions of aryl and heteroaryl chlorides in water. *Org. Biomol. Chem*. **2014**, *12*, 6944-6952.
25. Canivet, J.; Yamaguchi, J.; Ban, I.; Itami, K. Nickel-catalyzed biaryl coupling of heteroarenes and aryl halides/triflates. *Org. Lett.* **2019**, *11*, 1733-1736.
26. Xing, R.; Li, Y.; Liu, Q.; Han, Y.; Wei, X.; Li, J.; Zhou, B. Selective reduction of nitroarenes by a Hantzsch 1,4-dihydropyridine: A facile and efficient approach to substituted quinolines. *Synthesis* **2011**, *13*, 2066-2072.
27. Thakur, K. G.; Sekar, G. Copper(I)-catalyzed caryl-calkynyl bond formation of aryl iodides with terminal alkynes. *Synthesis* **2009**, *16*, 2785-2789.
28. Gooβen, L. J.; Rudolphi, F.; Oppel, C; Rodriguez, N. Synthesis of ketones from alpha-oxocarboxylates and aryl bromides by Cu/Pd-catalyzed decarboxylative cross-coupling. *Angew. Chem. Int. Ed.* **2008**, *47*, 3043-3045.
29. Huang, H.; Jing, X.; Deng, J.; Meng, C.; Duan, C. Enzyme-Inspired Coordination Polymers for Selective Oxidization of C(sp(3))-H Bonds via Multiphoton Excitation. *J. Am. Chem. Soc*. **2023**, *145*, 2170-2182.
30. Rao, M. L. N.; Venkatesh, V.; Banerjee, D. Atom-efficient cross-coupling reactions of triarylbismuths with acyl chlorides under Pd(0) catalysis. *Tetrahedron* **2007**, *63*, 12917-12926.
31. Tao, J.-L.; Yang, B.; Wang, Z.-X. Pincer-Nickel-Catalyzed Allyl-Aryl Coupling between Allyl Methyl Ethers and Arylzinc Chlorides. *J. Org. Chem.* **2015**, *80*, 12627-12634.
32. Rao, M. L. N.; Dhanorkar, R. J. Pd-catalyzed chemoselective threefold cross-coupling of triarylbismuths with benzylic bromides. *RSC Adv*. **2013**, *3*, 6794–6798.
